# Design and synthesis of anticancer 1-hydroxynaphthalene-2-carboxanilides with a p53 independent mechanism of action

**DOI:** 10.1038/s41598-019-42595-y

**Published:** 2019-04-23

**Authors:** Ewelina Spaczyńska, Anna Mrozek-Wilczkiewicz, Katarzyna Malarz, Jiri Kos, Tomas Gonec, Michal Oravec, Robert Gawecki, Andrzej Bak, Jana Dohanosova, Iva Kapustikova, Tibor Liptaj, Josef Jampilek, Robert Musiol

**Affiliations:** 1 Institute of Chemistry, University of Silesia, 75 Pułku Piechoty 1a, 41-500 Chorzów, Poland; 2 A. Chełkowski Institute of Physics and Silesian Center for Education and Interdisciplinary Research, University of Silesia, 75 Pułku Piechoty 1a, 41-500 Chorzów, Poland; 3 Department of Pharmaceutical Chemistry, Faculty of Pharmacy, Comenius University, Odbojarov 10, 832 32 Bratislava, Slovakia; 4 Department of Chemical Drugs, Faculty of Pharmacy, University of Veterinary and Pharmaceutical Sciences, Palackeho 1, Brno, 612 42 Czech Republic; 5 Global Change Research Institute CAS, Belidla 986/4a, Brno, 603 00 Czech Republic; 6 Central Laboratories, Faculty of Chemical and Food Technology, Slovak University of Technology in Bratislava, Radlinskeho 9, Bratislava, 81237 Slovakia; 7 Department of Analytical Chemistry, Faculty of Natural Sciences, Comenius University, Ilkovicova 6, 842 15 Bratislava, Slovakia; 8 Regional Centre of Advanced Technologies and Materials, Faculty of Science, Palacky University, Slechtitelu 27, 783 71 Olomouc, Czech Republic

**Keywords:** Colon cancer, Cheminformatics, Microwave chemistry

## Abstract

A series of 116 small-molecule 1-hydroxynaphthalene-2-carboxanilides was designed based on the fragment-based approach and was synthesized according to the microwave-assisted protocol. The biological activity of all of the compounds was tested on human colon carcinoma cell lines including a deleted TP53 tumor suppressor gene. The mechanism of activity was studied according to the p53 status in the cell. Several compounds revealed a good to excellent activity that was similar to or better than the standard anticancer drugs. Some of these appeared to be more active against the p53 null cells than their wild-type counterparts. Intercalating the properties of these compounds could be responsible for their mechanism of action.

## Introduction

Small-molecule drugs are still most commonly used in the treatment of cancer. However, the cytostatic agents and DNA poisons that were initially developed for clinical use are losing their position today. The particularly offensive, and often even life-threatening and difficult to restrain side effects, significantly degrade the quality of life of patients and are often responsible for the cessation of therapy. This is the result of a non-specific mechanism of action and the low selectivity of these drugs. In fact, some alkylating agents are more toxic on normal cells than on malignant cells. Another problem that is often encountered is the resistance that may emerge after a brief period of a positive reaction to the therapy or may even occur in drug-naïve patients^1^. This phenomenon is also an obstacle in so-called targeted therapies that use novel agents such as kinase inhibitors as well as in non-small therapeutics such as monoclonal antibodies^2^. Therefore, although novel small-molecule agents are still in demand, newly designed compounds are required to have a specific even multitargeted mechanism of action and a good selectivity over normal cells.

**Figure 1:**
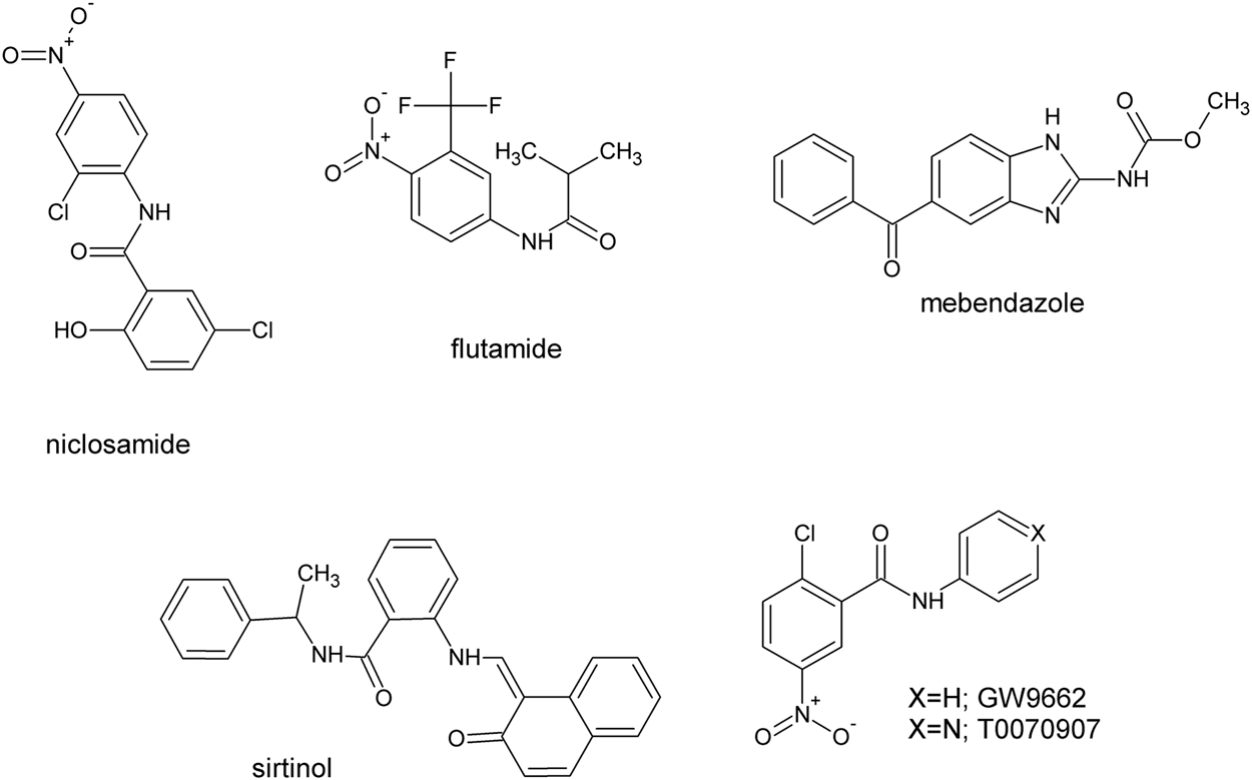
Small molecule anilides revisited as anticancer agents.

Because of their resemblance to biological structures and their great synthetic availability, simple aromatic amides are interesting scaffolds for designing new drugs. However, the majority of the literature data deals with their antimicrobial or anti-infectious activity and relatively few reports focus on their antiproliferative and anticancer potency (Fig. 1)^3,4^. Even though salicylanilide has been claimed as privileged structure^5^, these compounds are only being developed as antibacterial^4,6–8^, antimycobacterial^9–14^, antifungal^15,16^ and antiprotozoic/antihelmintic^16,17^ agents or as photosynthesis inhibitors^18–20^. Recently, some appealing reports on simple antihelmintic drugs that have been used for quite some time have been published. For example, mebendazole was found to be effective in selectively inducing apoptosis in cancer cells both *in vitro* and *in vivo*^21^. Later, the antiproliferative activity of mebendazole and its analog albendazole was connected with the inhibition of the hedgehog signaling pathway^22^. In addition, niclosamide, which has been used for decades as an antiparasitic drug, was rediscovered as an effective m-TOR inhibitor in a large library screening in 2009^23,24^.

This resulted in a more thorough investigation that revealed its wide spectrum of activity against various cancer types. However, niclosamide’s multitargeted mechanism of action, which includes the inhibition of Wnt/β-catenin, STAT3, NF-κB, among others, is even more appealing^25^. It was effective in hampering cell migration and metastasis, thereby reducing the invasive potential of cancer cells^26,27^. This complex mechanism of action corresponds with its ability to overcome resistance in cancer cells^28,29^. Liu *et al*. recently reported that niclosamide is also capable of inhibiting the isoforms of the spliced androgen receptor (AR) and in overcoming drug-resistance in prostate cancers^30^. Enzalutamide is an androgen receptor antagonist that is used in the treatment of prostate cancer. However, in the most severe castration-resistant metastatic cancers, spliced variants of AR have been recognized. These forms are crucial in developing a resistance to antiandrogens including enzalutamide and flutamide^31^. A series of niclosamide derivatives with a strong anticancer potency have been identified in recent literature, e.g. the GW9662 and T0070907, which are potent inhibitors of PPAR-γ^32,33^. Moreover, in addition to its antiandrogen activity, flutamide may have some anticancer effects *via* another mechanism, as was recently reported for hepatocellular carcinoma^34^. These facts inspired us to perform a more in-depth investigation of the antiproliferative activity of a series of salicylanilides that had been designed based on those active structures. We decided to exploit the naphthalene skeleton to increase its lipophilicity and affinity to DNA and the replicative enzymes^35^. The naphthalene-bearing structures also peaked our interest to investigate their antitumor activities. In fact, the non-steroidal anti-inflammatory drug naproxen has also been found to have an antiproliferative effect against various cancer cells^36,37^. It has also been exploited as a leading structure in the search for new anticancer agents. Moreover, as was reported by Husain *et al*., even such a small and simple molecule may intercalate to DNA and cause photo-induced damage by reactive oxygen species^38^. Recently, some ring-substituted hydroxynaphthanilides have been synthesized and their anticancer activity has been reported^39^. In our continuing search for potential drug candidates, the present study describes the synthesis and antiproliferative evaluation of *N-*substituted 1-hydroxynaphthalene-2-carboxanilides. In this paper, we further report on the naphthalene compounds that exhibited strong antitumor activities against the human cancer cell lines.

## Results

**Figure 2:**
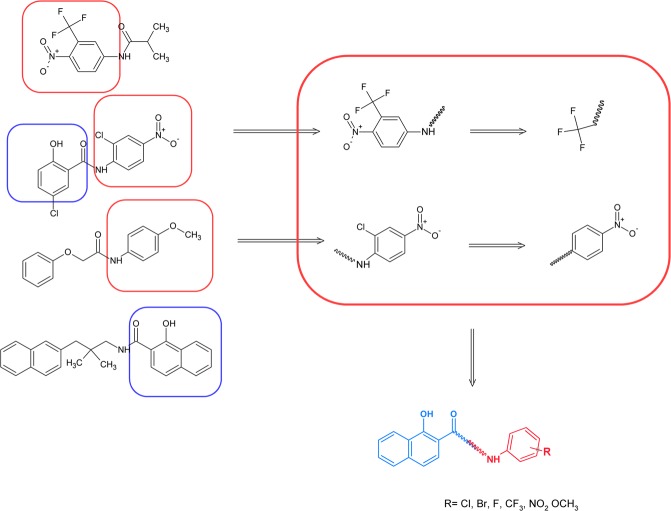
Design of the target amides. Compounds with an anticancer activity provided amine-derived fragments, which were further divided into substituents. The final structure was a combination of all of the fragments.

Naphthanilides are conjugates of naphthanilic acid and aromatic amines, which also results in their synthetic availability. On the other hand, no specific target has yet been proposed for similar compounds and their mechanism of action appears to be multitargeted as was described for niclosamide or its analogs. With this in mind, we used the method of the decremental isolation of the synthons from known anticancer agents such as niclosamide and flutamide and similar small-molecule drugs/agents that are available by database mining^5,40–43^. An overview of this approach is presented in Fig. 2.

The substitution pattern in the amine moiety that was selected in this approach can easily be covered by a commercial library of building blocks. This method seems to be particularly suitable for designing easily obtainable compounds that have a common structural motif as we reported for anticancer thiosemicarbazones^44,45^ or antiretroviral amides^46^. 1-Hydroxy-2-naphthoic acid was selected for the final scaffold as was mentioned earlier. The discriminative fragments that were harvested from the database search were used as the substituents in an aniline moiety. Due to the lack of leading structures or target specific properties, there were no clues as to a substitution pattern. For this reason, we decided to obtain all of the isomeric structures that were available (i.e. *ortho, meta* and *para* for each substituent), which permitted a deeper and more precise investigation of the structure-activity relationships.

### Chemistry

There are many methods that can be used to prepare carboxanilides. The compounds that were used were synthesized using a one-pot microwave-assisted synthesis^47^. Microwave-assisted organic chemistry is well known for its high efficiency and good yield and purity and can be used in synthesis of a wide assortment of compounds including nanomaterials^48,49^, multicomponent reactions^50,51^ and heterocyclic compounds^52,53^ among others. This approach can be also used for homo-and hetero-catalyzed reactions^54,55^. It is also very useful in the synthesis of fragile compounds that decompose easily, for example, in the synthesis ofcarbohydrates^56^ or for the introduction of nitro groups^57^. In the synthesis of larger libraries of compounds such an approach has significant advantages^58,59^.

**Figure 3:**
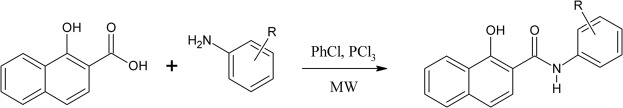
Synthesis of ring-substituted 1-hydroxynaphthalene-2-carboxanilides **1–8d**. Microwave irradiation (MW) conditions: 120–130 °C; 500 W; 50 minutes R = H, OCH_3_, F, Cl, Br, CF_3_, NO_2_.

The condensation of 1-hydroxy-2-naphthoic acid with ring-substituted anilines using phosphorus trichloride in chlorobenzene under microwave conditions yielded a series of *N*-substituted 1-hydroxynaphthalene-2-carboxanilides **1–8d**. The carboxyl group was activated with phosphorus trichloride first. The final amide was immediately formed *via* the aminolysis of acyl chloride by ring-substituted aniline in dry chlorobenzene. The tentative mechanism of this synthesis seems to be identical to conventional conditions without any special microwave effects. The compounds that were studied were prepared according to Fig. 3. All of the compounds were recrystallized from ethyl acetate or a mixture of solvent ethanol/water. Their HPLC purity exceeded 98%, and the yields were more than 70% in most cases.

### Biological activity tests

#### Antiproliferative assay

All of the synthesized compounds were tested for their antiproliferative activity against human colon cancer (Table 1). We used both the wild-type and p53-negative cell lines (HCT116^+/+^ and HCT116^−/−^, respectively). Mutations in the TP53 gene are present in more than 50% of all cancers and often specifically correspond to a difficult therapy, resistance or a bad prognosis^60–63^. The active compounds were additionally tested for their cytotoxicity against normal human fibroblasts. We used 5-fluorouracil (5-FLU), doxorubicin (DOX) and CP-31398 as the standards. 5-FLU is one of the first antimetabolites that was dedicated for cancer treatment and is still used for a wide range of cancers including colon carcinoma^64^. Doxorubicin is a DNA topoisomerase II poison with a high intercalating potency, which is able to induce apoptosis through the p53 pathway^65^. Although DOX has been described as being ineffective in some p53 mutants, its level of activity against the p53 null lines is similar^66^. CP-31398, on the other hand, is an experimental drug in the clinical phase and its mechanism of action is through the reactivation some of p53 mutants. Its activity against the p53 null cell lines is greatly diminished and is actually lower than its cytotoxicity^67^.

**Table 1:** The antiproliferative activity of ring-substituted 1-hydroxynaphthalene-2-carboxanilides.

No.		Activity IC_50_ [μM]		
		HCT116^+/+^	HCT116^−/−^	NHDF
**1**	H*	>25	>25	—
**2a**	2-OCH_3_*	>25	>25	—
**2b**	3-OCH_3_*	8.51 ± 1.69	24.05 ± 3.54	>25
**2c**	4-OCH_3_*	>25	>25	—
**2d**	2,5-OCH_3_	>25	>25	—
**2e**	3,5-OCH_3_	>25	>25	>25
**2f**	3,4,5-OCH_3_	6.54 ± 2.42	8.78 ± 2.13	>25
**2g**	2-OCH_3_-4-NO_2_	2.62 ± 0.85	3.73 ± 1.09	7.53 ± 2.97
**2h**	2-OCH_3_-5-NO_2_	7.05 ± 1.75	7.42 ± 2.05	>25
**2i**	2-OCH_3_-5-CF_3_	>25	>25	—
**2j**	2-OCH_3_-5-CH_3_	>25	>25	—
**2k**	2-OCH_3_-6-CH_3_	>25	>25	—
**3a**	2-CH_3_*	>25	>25	—
**3b**	3-CH_3_*	>25	>25	—
**3c**	4-CH_3_*	>25	>25	—
**3d**	2,5-CH_3_	>25	>25	—
**3e**	2,6-CH_3_	>25	>25	—
**3f**	3,5-CH_3_	19.31 ± 1.68	>25	>25
**3g**	2,4,6-CH_3_	>25	>25	—
**3h**	2-CH_3_-5-OCH_3_	11.65 ± 3.81	>25	>25
**3i**	2-CH_3_-5-CF_3_	8.41 ± 1.07	5.08 ± 1.23	>25
**4a**	2-F*	>25	>25	—
**4b**	3-F*	1.88 ± 0.33	9.02 ± 1.91	>25
**4c**	4-F*	2.49 ± 0.61	5.01 ± 1.12	>25
**4d**	2,4-F	6.55 ± 0.83	11.41 ± 1.92	>25
**4e**	2,5-F	4.76 ± 0.76	15.05 ± 6.75	>25
**4f**	2,6-F	>25	>25	—
**4g**	3,4-F	3.04 ± 0.06	5.88 ± 0.50	22.21 ± ± 1.61
**4h**	3,5-F	1.79 ± 0.44	2.24 ± 0.30	9.87 ± 0.66
**4i**	2,3,4-F	4.28 ± 0.66	4.43 ± 0.66	>25
**4j**	2,4,5-F	1.87 ± 0.35	4.75 ± 0.05	24.52 ± 0.03
**4k**	2,4,6-F	>25	>25	—
**4l**	3,4,5-F	1.19 ± 0.31	1.42 ± 0.25	10.04 ± 0.54
**4m**	2,3,5,6-F	11.95 ± 0.92	>25	>25
**4n**	2,3,4,5,6-F	11.59 ± 0.74	17.54 ± 0.76	>25
**4o**	2-F-3-Cl	8.78 ± 2.18	6.76 ± 2.37	>25
**4p**	2-F-3-CF_3_	6.50 ± 1.16	5.45 ± 1.72	24.44 ± 2.51
**4q**	2-F-4-Cl	9.89 ± 3.26	9.69 ± 3.13	>25
**4r**	2-F-4-Br	14.99 ± 4.82	3.43 ± 0.69	>25
**4s**	2-F-5-Cl	8.66 ± 1.61	10.66 ± 1.73	>25
**4t**	2-F-5-Br	11.94 ± 1.28	14.01 ± 2.2	>25
**4u**	2-F-5-CF_3_	10.05 ± 2.61	7.45 ± 1.74	>25
**4v**	3-F-4-Br	10.86 ± 2.69	12.43 ± 1.11	>25
**4w**	3-F-4-CF_3_	3.25 ± 0.94	4.82 ± 1.46	22.82 ± 2.69
**4×**	3-F-5-CF_3_	9.01 ± 0.45	>25	24.12 ± 2.02
**4y**	2,3,5,6-F-4-Br	2.41 ± 0.69	5.68 ± 1.37	7.87 ± 1.37
**4z**	2,5-F-4-CF_3_	4.22 ± 0.87	2.82 ± 0.77	18.35 ± 0.91
**5a**	2-Cl*	16.49 ± 2.25	22.44 ± 3.58	>25
**5b**	3-Cl*	4.70 ± 0.40	1.78 ± 0.32	>25
**5c**	4-Cl*	9.62 ± 0.75	9.41 ± 0.78	>25
**5d**	2,3-Cl^‡^	4.66 ± 0.58	8.25 ± 0.64	>25
**5e**	2,4-Cl^‡^	1.95 ± 0.50	6.06 ± 1.13	>25
**5f**	2,5-Cl^‡^	7.45 ± 0.85	7.19 ± 1.15	>25
**5g**	2,6-Cl^‡^	>25	>25	—
**5h**	3,4-Cl^‡^	5.15 ± 0.50	4.74 ± 0.82	>25
**5i**	3,5-Cl^‡^	2.32 ± 0.26	2.89 ± 0.38	11.47 ± 0.88
**5j**	2,4,5-Cl^‡^	3.00 ± 0.24	6.21 ± 0.66	20.19 ± 0.79
**5k**	2,4,6-Cl^‡^	19.37 ± 1.27	>25	>25
**5l**	3,4,5-Cl^‡^	1.31 ± 0.15	2.86 ± 0.33	7.01 ± 0.42
**5m**	2-Cl-4-F	7.57 ± 2.16	8.29 ± 2.92	>25
**5n**	2-Cl-4-Br^‡^	2.33 ± 0.95	9.79 ± 1.95	23.45 ± 0.86
**5o**	2-Cl-4-CF_3_	4.92 ± 1.32	5.41 ± 1.11	13.18 ± 1.97
**5p**	2-Cl-5-OCH_3_	24.10 ± 2.58	19.31 ± 2.09	>25
**5q**	2-Cl-5-Br^‡^	4.18 ± 1.49	5.40 ± 2.04	>25
**5r**	2-Cl-5-CF_3_	2.48 ± 0.31	1.46 ± 0.21	12.40 ± 1.14
**5s**	3-Cl-4-F	6.87 ± 2.48	0.83 ± 0.31	>25
**5t**	3-Cl-4-Br^‡^	12.35 ± 0.79	12.58 ± 0.69	>25
**5u**	2-Cl-3,5-CF_3_	0.72 ± 0.28	2.72 ± 0.63	16.30 ± 1.28
**5v**	2,6-Cl-4-CF_3_	>25	>25	—
**6a**	2-Br*	22.51 ± 3.62	>25	>25
**6b**	3-Br*	9.19 ± 0.98	8.73 ± 0.89	15.21 ± 2.48
**6c**	4-Br*	5.25 ± 0.67	11.01 ± 0.97	>25
**6d**	2,4-Br^‡^	4.56 ± 0.43	4.61 ± 0.72	>25
**6e**	2,5-Br^‡^	5.24 ± 0.28	5.85 ± 0.61	>25
**6f**	2,6-Br^‡^	>25	>25	—
**6g**	2,4,6-Br^‡^	>25	>25	—
**6h**	2-Br-4-Cl^‡^	9.54 ± 2.71	13.23 ± 2.3	>25
**6i**	2-Br-4-CF_3_	3.58 ± 1.25	2.19 ± 0.67	18.42 ± 1.23
**6j**	2-Br-5-F	7.78 ± 1.37	5.83 ± 1.06	>25
**6k**	2-Br-5-CF_3_	8.18 ± 1.72	7.15 ± 1.96	22.23 ± 0.83
**6l**	2,6-Br-4-CF_3_	9.37 ± 0.47	6.81 ± 2.66	20.37 ± 1.16
**6m**	2,6-Br-3-Cl-4-F	>25	>25	—
**7a**	2-CF_3_*	>25	21.36 ± 1.24	—
**7b**	3-CF_3_*	6.25 ± 0.47	1.39 ± 0.29	>25
**7c**	4-CF_3_*	4.31 ± 0.51	1.07 ± 0.26	16.57 ± 2.17
**7d**	2,4-CF_3_	11.31 ± 1.31	8.69 ± 1.02	>25
**7e**	2,5-CF_3_	4.44 ± 1.16	8.53 ± 3.37	22.93 ± 2.10
**7f**	3,5-CF_3_	0.46 ± 0.05	0.35 ± 0.08	3.55 ± 0.76
**7g**	2-CF_3_-4-F	>25	>25	—
**7h**	2-CF_3_-4-Cl	>25	>25	—
**7i**	2-CF_3_-4-Br	>25	>25	—
**7j**	2-CF_3_-4-NO_2_	2.04 ± 0.71	4.74 ± 1.68	12.55 ± 1.32
**7k**	3-CF_3_-4-OCH_3_	>25	>25	—
**7l**	3-CF_3_-4-CH_3_	>25	>25	—
**7m**	3-CF_3_-4-F	11.96 ± 3.68	14.49 ± 4.53	>25
**7n**	3-CF_3_-4-Cl	9.66 ± 3.17	18.72 ± 2.78	>25
**7o**	3-CF_3_-4-Br	>25	13.90 ± 3.75	>25
**7p**	3-CF_3_-4-NO_2_	1.64 ± 0.47	1.39 ± 0.44	9.68 ± 1.49
**8a**	2-NO_2_*	23.32 ± 3.77	>25	22.71 ± 2.71
**8b**	3-NO_2_*	6.82 ± 1.17	3.10 ± 0.63	>25
**8c**	4-NO_2_*	0.41 ± 0.05	0.69 ± 0.09	12.30 ± 1.49
**8d**	2-NO_2_-4-CF_3_	>25	>25	—
—	5-FLU	4.42 ± 0.70	4.69 ± 0.31	>25
—	DOX	0.34 ± 0.04	0.38 ± 0.03	3.38 ± 1.29
—	CP-31398	18.63 ± 0.92	26.28 ± 1.41	12.26 ± 0.54

*Compounds described in^88^, ^‡^compounds described in^18^.

### Modeling the drug activity using the CoMFA/CoMSA and SMV procedure

The main objective of the ligand-based modeling was to perform a systematic study of the performance of CoMFA/CoMSA in modeling the *in vitro* activity that was observed for the set of 1-hydroxynaphthalene-2-carboxanilides derivatives, which showed a high antiproliferative activity against human colon cancer. Hence, the pharmacophore properties of a target series using a coupled neural network and the PLS method with the variable elimination IVE procedure were scrutinized. We compared the findings of the activity modeling using the standard 3D methodology (CoMFA) and its neural counterparts (CoMSA) regarding multiple training/test subsets and the (in)dependent variables being used. Unfortunately, the ${q}_{cv}^{2}$ performance of the toxic profile for the entire hydroxynaphthanilide-based dataset **1**–**8d** in the training dataset was not satisfactory for the CoMFA (${q}_{cv}^{2}$ ≈ 0.5, SDEP ≈ 0.30) and CoMSA models (${q}_{cv}^{2}$ ≈ 0.5, SDEP ≈ 0.25), regardless of the probe atom type (CH_3_^+^, H^+^, CH_3_^0^) or the map size (10 × 10 ÷ 30 × 30) and the template molecules that were applied (**1**, **2f**, **4l**, **4n**, **7f**, **8c**). However, the quality of the HCT116^+/+^ models that were generated in terms of ${q}_{cv}^{2}$ was slightly better compared to HCT116^−/−^ and NHDF. It seems that the following rank of ${q}_{cv}^{2}$(HCT116^+/+^) > ${q}_{cv}^{2}$(HCT116^−/−^) > ${q}_{cv}^{2}$(NHDF) values is partially determined by the number of constants or missing activity data, e.g. ≈ 30% of HCT116^+/+^, ≈ 35% of HCT116^−/−^ and ≈72% of NHDF, respectively. It is worth mentioning, that the molecular lipophilicity that was evaluated by the calculated logP value (clogP) as an extension of the descriptor pools had only a minor effect on the modeling outcomes. It is obvious that the predictive power of a model cannot be evaluated only by the goodness of fit of the data with the cross-validated leave-one-out procedure (CV-LOO); therefore, the exclusive reliance on a training set is insufficient to specify the robustness of models^68^. Consequently, an external validation in which the molecule subset was divided into a training/test collections was applied to evaluate the predictive ability of the model with SDEP and ${q}_{test}^{2}$ statistics. The CoMFA/CoMSA performance for the models, which were arbitrarily divided into the training/test subsets at a 2:1 ratio (68/34) and ranked according to the antiproliferative activity (in HCT116) was also examined. Additionally, the Kennard-Stone algorithm was used on the dependent variables to divide the data collection into training/test subgroups representatively^68^. In all of the cases, the best CoMFA/CoMSA ${q}_{cv}^{2}$/${q}_{test}^{2}$ outcomes performed comparably and indicated pretty poor model abilities, which were accompanied by a poor predictive power of the model. The obtained findings confirmed that *a priori* dividing objects into training/test subgroups with a restricted ensemble of molecules that is assigned with activity data is not a trivial issue; therefore, an additional assessment, namely the Stochastic Model Validation (SMV), was conducted as a ‘perturbation’ procedure to examine the data structure^69^.

Consequently, the repetitive sampling of the original compound ensemble (102 molecules) into training/test subseries each containing 68/34 molecules (fraction 2/3 to 1/3) was conducted iteratively in order to determine any variations in the statistical estimators. Due to resource and time constraints, the examination of all of the possible combinations ${C}_{102}^{34}$ ≈ 1.31 × 10^27^ was not technically feasible, and therefore the overall number of samplings was restricted to a relatively small fraction of 10^6^ populations, which were systematically generated and subsequently used in the PLS modeling. The observed distribution of the ${q}_{cv}^{2}$ vs. ${q}_{test}^{2}$ pattern confirmed the intuitive interpretation of the ${q}_{cv}^{2}$/${q}_{test}^{2}$ fluctuation pattern, where a higher modeling power within the training set can be specified for the HCT116 potency (${q}_{cv}^{2}$ ≥0.70). Conversely, the preferential selection of the objects into the training sets that fit into the model, resulted in a decrease in the predictive ability for the residual objects, which confirmed the dual nature of the ${q}_{cv}^{2}$/${q}_{test}^{2}$ parameters, in which a high value of ${q}_{cv}^{2}$ does not imply a good model predictability^69^.

**Figure 4:**
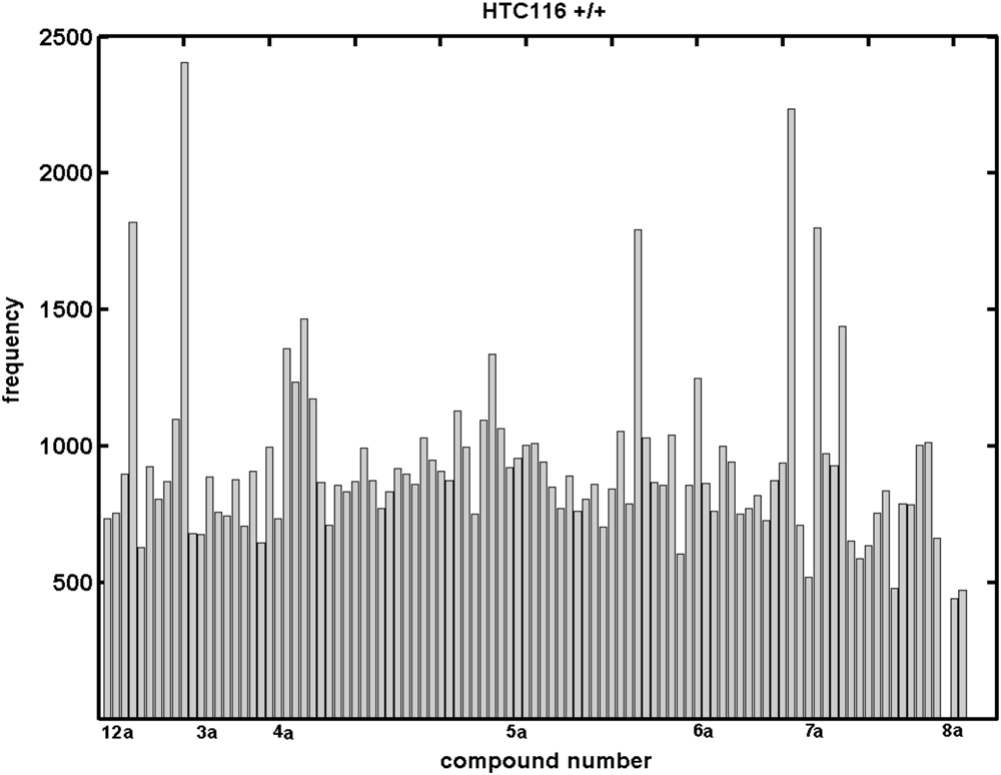
Number of individual compounds that appeared in the test set within >0.6 and >0 for the HCT116^+/+^ potency of the hydroxynaphthanilide derivatives using the CoMSA method.

On the other hand, it should be stressed that the *great advantage of the QSAR/QSPR paradigm does not lie in the extrapolation*, and therefore we focused more on the *descriptive aspects* of the molecular modeling. Interestingly, the answers to the inspected training/test perturbations (${q}_{cv}^{2}$> 0.5 & ${q}_{test}^{2}$>0) in the form of population maps revealed that the HCT116^+/+^ models formed more densely populated clusters compared to HCT116^−/−^. Figure 4 illustrates the molecule selection frequency into the test sets as a function of the compound number when sampling the best models (${q}_{cv}^{2}$ ≥0.60 & ${q}_{test}^{2}$>0). Noticeably, the relatively smooth compound distribution within the training/test subpopulations was disturbed by the outnumbering of 25 molecules with a count frequency ≥1,000 (**2c**, **2h**, **2i**, **4a**-**4d**, **4q**, **4u**, **4y**, **4z**, **5c**, **5d**, **5n**, **5o**, **5p**, **5q**, **5t**, **6a**, **6d**, **6l**, **7b**, **7e**, **7m**, **7o**). Generally speaking, the specified molecules were mainly *ortho-* and/or *para-*substituted isomers with electron-withdrawing substituents. Interestingly, the anticancer activity for the mono-positioned isomers can be ranked according to a rough relation in which *ortho* <*meta/para*, which partly explains the preferential selection of the *meta-*positioned molecules into a training subset. Following the SMV CoMSA findings, the indicated molecules were also eliminated from the training sets in the CoMFA modeling of the activity profile (test sets: **2c**, **2h**, **2i**, **4a**-**4d**, **4q**, **4u**, **4y**, **4z**, **5c**, **5d**, **5n**, **5o**, **5p**, **5q**, **5t**, **6a**, **6d**, **6l**, **7b**, **7e**, **7m**, **7o**). In this case, the best CoMFA/CoMSA models were comparable (CoMSA: ${q}_{cv}^{2}$ = 0.71 vs. CoMFA ${q}_{cv}^{2}$ = 0.73).

### Induction of apoptosis

**Figure 5:**
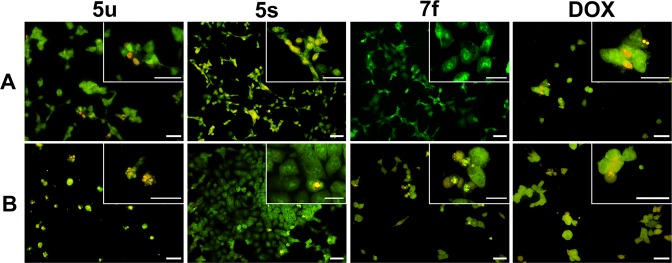
The morphological changes of the HCT116 p53^+/+^ (**A**) and HCT116 p53^−/−^ (**B**) cells after a 48-hour treatment with **5u**, **5s** and **7f**. The cells were stained with AO/EB to indicate apoptosis. The cells that had been treated with anilides showed early apoptotic features – green and yellow cells (green dots in the nuclei indicate chromatin condensation and nuclear fragmentation) and late apoptotic cells – orange cells with condensed and fragmented nuclei. Scale bars = 50 μm.

Three compounds with a distinct activity were selected for further tests. These were **7 f**, which was the most active with no selectivity; **5u**, which was roughly four times more active against the wild type and **5s** due to its preference for the p53 null cells. The apoptosis of the cells that were treated with the active compounds was confirmed using the Acridine Orange (AO)/Ethidium Bromide (EB) staining test^70^ (Fig. 5). For active compounds **5u**, **5s** and **7f**, we observed early and late apoptotic cells in the HCT116 colonies.

The cells were stained with AO/EB to indicate apoptosis. The cells that were treated with anilides showed early apoptotic features – green and yellow cells (green dots in the nuclei indicate chromatin condensation and nuclear fragmentation) and late apoptotic cells – orange cells with condensed and fragmented nuclei.

**Figure 6:**
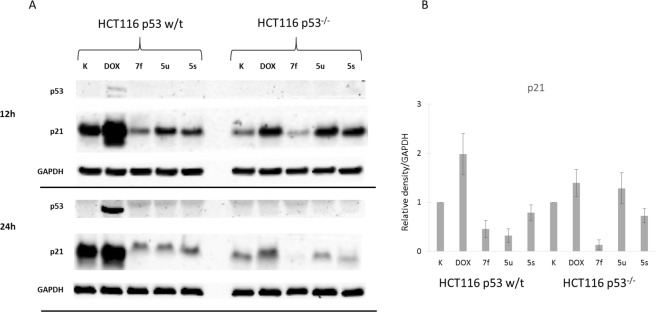
Influence of the active anilides and DOX on the activation of the p53/p21 system in the HCT116 cells (**A**). A densitometric analysis of the expression of the p21 protein normalized to GAPDH. The results are the mean ± SD of three independent experiments (**B**). Uncropped expositions are presented in Figure S5 in the Supplementary Information.

**Figure 7:**
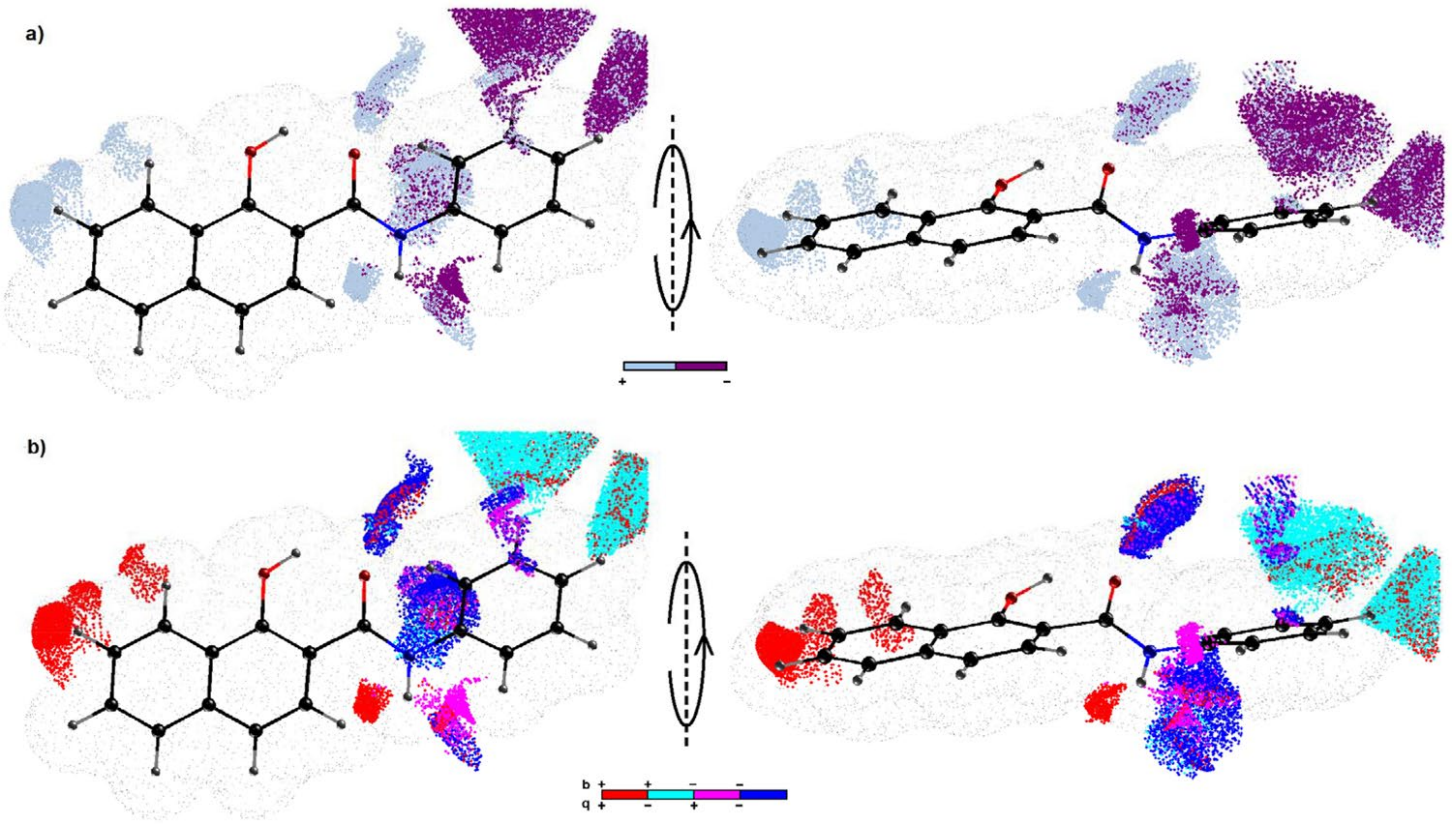
CoMSA IVE-PLS monitored for the 68/34 training/test set samplings. The plots show the spatial areas with the strongest influence to the HCT116^+/+^ activity. Colors coding the sign of this impact **(a)**. Four possible combinations of the mean charge and correlation coefficient are color coded **(b)**. Compound 1 as a reference molecule was plotted in two different orientations.

Further exploration of the cell death model was performed by a western blot analysis of protein activation in response to incubation with the tested compound (Figs 6, 7). As was expected, incubation with DOX led to a dramatic increase in the amounts of the p21 and p53 proteins in the wild-type colon carcinoma cells. The proapoptotic activation of the p53/p21 system was noticeable after the first 12 hours and then became stronger after 24 h. This effect was not observed in the case of the p53 null cells, which were unable to synthesize this protein. In contrast, the three tested anilides did not cause the activation of p53. However, a small, insignificant increase of p21 was observed for **5u** and **5s** in the p53 null cells after the first 12 hours. It was also noticeable that the non-selective compound **7f** did not increase p21 in either of the lines that were tested.

### DNA intercalation

The fused aromatic rings of the naphthalene moiety and halogen substituents in aniline may guarantee good intercalating properties^71,72^. On the other hand, the intercalation of DNA may explain the high activity against both the wild-type and p53 mutants as was reported for a series of styrylquinolines^67^. Binding to DNA causes the bases to mismatch or cleave, which results in the arrest of the cell cycle and apoptosis. The effectiveness of the p53 system is one of the most important factors in the response to therapy^73^. We tested the intercalating properties of the selected compounds in a spectrophotometric assay on calf-thymus DNA. The absorption spectra of the tested compounds in the absence and presence of CT-DNA are presented in Figure S1 Supplementary Information and the spectral properties with doxorubicine and CP-31398 are summarized in Table 2.

**Table 2:** Absorption spectral properties of the tested compounds bound to CT-DNA.

Comp	Absorption λ_max_ [nm]	Changes in absorbance	% hypochromism	Δε [M^−1^cm^−1^]	red shift* [nm]
**5s**	264; 380	hypochromism	6.5	255.6	2
**5u**	282; 380	hypochromism	14.0	922.2	2
**7f**	282; 382	hypochromism	5.7	244.4	0
**DOX**	480	hypochromism	34.2	3235.6	10
**CP-31398**	312; 350	hypochromism	41.4, 37.4	1515.6; 1693.3	0

*for the wavelengths of the maximum absorption for the individual and DNA-bound compounds.

## Discussion

Generally, most of the compounds that were tested in this study appeared to be active and more than 50 reached a micromolar level of activity (IC_50_ <10 μM), which is comparable with the standard drugs. The most active compounds were **8c** and **7f**, which had a submicromolar activity level and IC_50_ 0.415 and 0.464, respectively, for the HCT116 wild type. However, **8c** also had a preferable low cytotoxicity against the normal cell line and a selectivity index of 30. Noticeably, the p53 status had little effect on the majority of the compounds that appeared to be active. In many cases, the wild-type colon carcinoma was more sensitive to those anilides. However, some of the agents that were tested were particularly interesting because of their intriguing selectivity towards the p53 null cells. Among them, **5s** was more than eight times more toxic against HCT116 p53^−/−^ than its wild-type counterpart. Its low cytotoxicity in normal cells (selectivity index >30) make it even more valuable. Surprisingly, other compounds with a substitution pattern that was similar to **5h**, **5t** and **4v** were not selective for the p53 mutant with the exception of **6j** and **4r**. Notably, **4r**, which is roughly 4.5-fold more effective against p53 null cells, shares a similar 2-fluoro substitution with **5s**. Generally, mono- and di-halogen are required as substituents for anticancer activity. This is clearly visible compared to the methyl or methoxy substituents (compare **2c** and **4c**). On the other hand, tetra- or penta-substituted naphthanilides are less active.

Moreover, Principal Component Analysis (PCA) for the set of descriptors that were derived from the Dragon 6.0 software was applied to the analyzed compounds. From the initial number of selected parameters (4,885), all of the columns with constant or nearly constant values (standard deviation <10^−4^) and with missing values were excluded at the preprocessing stage, thus resulting in a final set of 2,916 descriptors. Finally the dataset was organized in an *X*_102 × 2916_ matrix with the objects (molecules) gathered in rows and their numerical parameters in columns, for further analysis. The examination of any (dis)similarities between the objects that were studied required the simultaneous consideration of all of the calculated parameters. Thus, PCA was used to visualize any major variations in the performance of the investigated molecules according to their structure and profile of anticancer activity. The analysis was conducted for centered and standardized data, since the studied data library included parameters with various orders of magnitude. The percentage of modeled variance was applied to determine the number of significant principal components (PCs). The PCA model with the first four PCs described 70.72% of the total data variance, while the first three PCs accounted for 66.15%. Not surprisingly, the efficiency of the data compression when PCA was used implied a pretty strong correlation between the variables in the data set that was analyzed. An analysis of the score plots for PC1 vs. PC2, which are presented in Figures S2, S3 Supporting Information, indicated that the hydroxynaphthanilide analogs can be classified into groups according to their structural data – the positional isomers were generally grouped together. The mono-substituted halogen isomers (**4**–**6**) were located together as well as the mono-/di-/tri-substituted methyl and methoxy derivatives **2a**-**f**, **2j**, **2k**, **3a**-**h**. Compounds with the CF_3_ group and the halogen atom created a separate cluster (**4w**, **4×**, **5o**, **5r**, **6k**, **7g**, **7h**, **7i**, **7m**, **7n**, **7o**), which was similar to the di-halogenated positional isomers (**4d**-**h**, **5d**-**I**, **5q**-**t**, **6d**-**f**, **6h**). Additionally, 13 of the descriptors (see Table S1 Supplementary Information) that were produced by the Sybyl software were selected, including the count, volume, surface, Ro5 and lipophilicity parameters in order to investigate the variations within the set of hydroxynaphthanilide derivatives. The compression of the data increased slowly along with the number of PCs, that were considered. The first two PCs accounted for 79.47% of the total data variance and this increased to ca. 93.16% for the next four PCs. The projection of the objects onto the plane, which was defined by the PC1 vs. PC2 component (Figures S2, S3 Supporting Information), confirmed the dissimilarity of compound **5u** from the other molecules that were previously observed. Interestingly, a dense cluster of objects was observed along the first principal component (PC1 >0), in which the remaining ones were basically methoxy-based analogs. Compounds that are have similar structures, (chemotypes) should have similar property features – this is the working tenet of QSPR. The lipophilicity of the tested compounds (color-coded according to the values of clogP that were calculated) projected on the plane that was specified by the two components (PC1 vs. PC2) confirmed the tendency of the compounds to separate along PC1 as is illustrated in Figure S4 supporting information. Based on the loading plots, it can be concluded that the uniqueness of the above-mentioned molecules was caused by the positively correlated variables that described the molecular properties. However typically in mD-QSAR when enormous number of topologic/topographic-based descriptors are generated this increa the probability of variable overfitting and co-linearity. Thus elimination of variables is not a pre-processing procedure used to prune the input assembly/ensemble of the descriptors but it can be successfully used to simplify the interpretability of a model since fewer descriptor terms are examined. The automated IVE-PLS method was employed as a filter to identify the structural descriptors that had the highest individual weightings to the biological activity^69^. A simplified visual inspection of the pharmacophore pattern that was generated gave a clear 3D landscape of the areas that should be altered in order to improve the biological activity. Hence, all 68/34 training/test samples (specified for the regions with pretty high model abilities ${q}_{cv}^{2}$ ≥0.6 & ${q}_{test}^{2}$>0) were used to generate a combined pharmacophore. The columns with the highest stability for each of models that were randomly selected, were identified by the IVE-PLS methodology. The moment that ${q}_{cv}^{2}$ deteriorates indicate the number of columns that appeared relevant. Thus backward elimination of the columns war recurrently repeated until the optimal number of variables to be included within the model was achieved. The cumulative sum of the common columns for all of the investigated HCT116^+/+^ models was calculated and normalized to the range of [0 ÷ 1]. Originally, the group of columns with a value above the pre-selected cut-off of 0.8 was selected and the spatial pattern that is illustrated in Fig. 7 was generated by further filtering 80% of the CoMSA descriptors that had a relatively small statistical significance for the HCT116^+/+^ activity profile, respectively. The relative contribution of each variable was weighted by the magnitude and the corresponding regression coefficient, and color code was used to sign of the impact of a descriptor on the compound potency. The sign of the influence is color coded and not only depicts the regions with a positive or negative activity, but also for possible combinations of the mean charge or correlation coefficient.

The displayed arrangement of the 3D maps presented in Fig. 7 indicates the spatial areas that had a relatively huge impact (positive or negative) on the antiproliferative activities of the investigated molecules. The dark circles in Fig. 7a designate the fragments that are less favourable for the HCT116^+/+^ potency (due to steric hindrance or electrostatic factors). The bright polyhedral areas specifies the 3D patterns where an atom or substituent is foreseen to be positively influential for the antiproliferative activity.

Noticeably some regions in which a positive influence was indicated appeared similar to the amide bond which can be regarded as peptide-bond-like motif. The carbonyl moiety can contribute to the energy of the guest-host interaction (enthalpic factor) *via* the possible generation of hydrogen bond(s) with the target counterpart. Moreover, the hydrogen in N-H, can be important for the bonding affinity as hydrogen bond donor, which was designated by the positive regression coefficient area denoted in Fig. 7b. This illustrate the significance of the substituent R, attached directly to the phenyl ring (according to Fig. 3), and position *meta-* and *para-* seem to be critical factors for the activity of the compounds that were. Indeed, a mixed electrostatic influence to the activity can be noticed for the *meta* and *para* substitution (see Fig. 7b). Regarding the spatially allowed regions with the negative regression coefficient of CoMSA models, the molecules with higher electron density in the *meta* position positively contributed to the activity profile proportionally to the C-X bond length, e.g. C-F < C-Cl < C-Br. Conversely, the positive regression coefficients in this particular region probably mean that some polar (electropositive) atom(s) might enhance the potency of the HCT116^+/+^ compounds as was observed for the CF_3_- and NO_2_-substituted compounds within the population of *meta*/*para* structures. Moreover, the bulky substituents at position 4′ of the phenyl ring seems to be an unfavorable and explain the lower activity of the *para* isomers against HCT116^+/+^ cancer cells.

Deeper insight into the antiproliferative activity of the hydroxynaphthanilides included the cell death mode and the determination of the key proapoptotic proteins for selected compounds. Programmed cell death – apoptosis – can be triggered *via* several different pathways. The most typical one in response to cytotoxic agents such as the majority of anticancer drugs is the disruption of homeostasis, which leads to the activation of the p53 protein^74^. This tumor-suppressor protein is responsible for detecting cellular stress and initiating either DNA repair or cell suicide. In order to achieve this, p53 acts as a gene regulator and an activator of downward factors such as the p21 protein, thereby leading to the extrinsic and intrinsic pathways^74,75^. This protein is also activated in response to various anticancer drugs from antimetabolites to topoisomerase inhibitors and radiation^76,77^. The most common mechanism consists of cytochrome C leakage for the mitochondria, the activation of caspases and the formation of apoptosome^74^. Thus, a mutation in the TP53 gene often causes an increased drug resistance and a decreased treatment response. The p53/p21 system has been established to be the main trigger of apoptosis in colon carcinoma that is treated with doxorubicin^65^.

These results suggest that the tested anilides activate the p53-independent pathway of apoptosis, which can be supported by the observation of the proapoptotic proteins that were assayed. These are shown in Figure S5 supplementary information. Specifically, we did not observe the cleavage of caspases-8 and −9 to the tested compounds. An exception was **5s**, which caused some cleavage in the case of the p53 null cells but not in the wild-type cells. This observation corresponds with the high selectivity of **5s** towards the p53^−/−^ cell line. Moreover, no activation of caspase-8 or AIF was detected after incubation with the tested anilides. Similarly, the cytochrome c level was unaffected. Leakage of cytochrome c from the mitochondria is the first step of caspase-dependent apoptosis, which is often initiated by p53 activation^78^. With this in mind, these results confirm a p53-independent pathway as was suggested. On the other hand, cleavage of PARP as well as a small but evident increase in the PARP level in response to the active compounds was observed. PolyADP-ribose polymerase is the nuclear factor that is responsible for detecting and repairing single strand breaks in DNA^79^. Its inhibition by the cleavage that is induced with caspase-3 is also a classic sign of apoptosis. However, the complex role of this protein is still vague especially in terms of any alternative activation and deactivation factors^80,81^. Special attention is being paid to alternative ways of cell death for their potential usefulness in designing selectively targeted drugs^82–84^.

In general, the drug-DNA interactions may induce the hypo- or hyperchromism of the testing mixture. The latter reflects the electrostatic binding to the grooves or the partial uncoiling of the helix. Hypochromism, on the other hand, is attributed with the interaction of the electronic states of the ligand and DNA bases, tight complex and π-stacking and is typically regarded as a sign of intercalation. Usually, this effect is connected with a red or blue shift of the spectra. The strength of a wavelength shift and hyperchromism reveal the interaction strength. Although the tested compounds can be regarded as intercalators, their binding properties are lower than strong ligands such as DOX. Moreover, some interesting regularities can be observed. Compound **7f** revealed a rather low hypochromic effect and no wavelength shift, which may suggest that its activity is exerted by another mechanism. Two selective compounds showed signs of stronger interactions especially in the case of **5u** where a 14% lower intensity was connected with a small red-shift of the spectra. Moreover, **5s** revealed some intercalating properties, which may explain its activity and selectivity. Interaction with DNA results in a small amount of damage that may induce the repair system, arrest the cell cycle and ensure the survival of a cell with a fully functional p53. In mutants, however, incubation with even a small concentration of the compound may result in accumulating damage and cell death according to an alternative pathway. As was previously reported, the survival of a cell depends on the p53 activation from a stimulus that is triggered by DNA damage^85^. In p53-defective cells, the activation of PARP may not guarantee the successful escape from the death pathway, which is observed as a higher activity of the tested compounds, e.g. a lower IC_50_^67,86,87^.

To sum up, a series of aromatic 1-hydroxynaphthalene-2-carboxanilides were designed based on selecting decremental fragments from the literature data. One hundred sixteen compounds were synthesized according to the simple microwave-assisted method, which affords high yields and purity. All of the compounds were tested for their antiproliferative activities against the human colon cancer cell lines HCT116 wild type and with a deletion of the TP53 suppressor gene. The influence of the substitution pattern on the activity was revealed using COMSA and COMFA analyses. Several of those compounds showed a promising activity against the p53 mutants. Interactions with DNA seem to be involved in the mechanism of action that triggered apoptosis apparently on an s caspase-independent pathway. To summarize, our work shows that simple, small molecular aromatic amides may be valuable leading structures in the development of anticancer drugs.

## Materials and Methods

### Chemistry

All of the reagents were purchased from Sigma-Aldrich and Merck. Reactions were carried out in a StartSYNTH microwave lab station (Milestone, Sorisole BG, Italy). Thin layer chromatography was performed on alumina-backed silica gel 40 F_254_ plates (Merck, Darmstadt, Germany). The plates were illuminated under UV (254 nm) and evaluated in an iodine vapor. The melting points were determined on a Kofler hot-plate apparatus (HMK Franz Kustner Nacht BG, Dresden, Germany) and are uncorrected. The purity of the final compounds was checked by the HPLC separation module Waters Alliance 2695 XE (Waters Corp., Milford, MA, USA). A detection wavelength of 210 nm was used. The peaks in the chromatogram of the solvent (blank) were deducted from the peaks in the chromatogram of the sample solution. The purity of individual compounds was determined from the area peaks in the chromatogram of the sample solution. Infrared (IR) spectra were recorded on a Smart MIRacle™ ATR ZnSe for Nicolet™ Impact 410 FT-IR spectrometer (Thermo Scientific, West Palm Beach, FL, USA). The spectra were obtained by the accumulation of 64 scans with a 2 cm^−1^ resolution in the region of 4,000-650 cm^−1^. All ^1^H and ^13^C NMR spectra were recorded on a Bruker Avance III 400 MHz FT-NMR spectrometer (400 MHz for ^1^H and 101 MHz for ^13^C (Bruker Comp., Karlsruhe, Germany) or an Agilent VNMRS 600 MHz system (600 MHz for ^1^H, 151 MHz for ^13^C and 565 MHz for ^19^F (Agilent Technologies, Santa Clara, CA, USA) in DMSO-*d*_6_. Chemical shifts are reported in ppm (δ) using the signal of the solvent (DMSO-*d*_6_) as the reference (2.500, resp. 39.50) against the internal standard, Si(CH_3_)_4_. Trifluoroacetic acid was used as the standard for ^19^F NMR (δ = −76.55). High-resolution mass spectra were measured using a Dionex UltiMate^®^ 3000 high-performance liquid chromatograph (Thermo-Fisher Scientific, Waltham, MA, USA) coupled with a LTQ Orbitrap XL^TM^ Hybrid Ion Trap-Orbitrap Fourier Transform Mass Spectrometer (Thermo-Fisher Scientific) with an injection into HESI II in the positive or negative mode.

### General procedure for the synthesis of the *N*-(substituted-phenyl)-1-hydroxynaphthalene-2-carboxamides

1-Hydroxynaphthalene-2-carboxylic acid (2.66 mmol) and the corresponding substituted aniline (2.66 mmol) were suspended in 25 mL of dry chlorobenzene with phosphorous trichloride (1.33 mmol). The reacting mixture was heated in a microwave reactor at the maximal allowed power of 500 W and 130 °C using the infrared flask-surface control of the temperature for 15 min. The solvent was evaporated under reduced pressure. After evaporation to dryness, the product was washed with 2 M HCl and H_2_O. The crude product was purified by recrystallization from EtOAc or a mixture of EtOH/H_2_O.

Compounds **1**, **2a**–**c**, **3a**–**c**, **4a**–**c**, **5a**–**c**, **5d**–**l**, **5n**, **5q**, **5t**, **6d**-**h**, **6a**–**c**, **7a**–**c**, **8a**–**c** were described recently by Gonec *et al*.^88^.

*N*-(2,5-dimethoxyphenyl)−1-hydroxynaphthalene-2-carboxamide (**2d**). Yield 75%; Mp. 116–119 °C; HPLC purity 97.60%; IR (cm^−1^): 3433, 1634, 1604, 1595, 1538, 1489, 1453, 1413, 1387, 1325, 1276, 1248, 1208, 1200, 1172, 1150, 1125, 1045, 1021, 951, 866, 835, 806, 792, 762, 723, 711; ^1^H-NMR (DMSO-*d*_6_), δ: 13.66 (s, 1 H), 10.34 (s, 1 H), 8.33 (dd, 1 H, *J* = 7.7, *J* = 1.3 Hz), 8.07 (d, 1 H, *J* = 8.6 Hz), 7.91 (d, 1 H, *J* = 8.1 Hz), 7.67 (ddd, 1 H, *J* = 8.2, *J* = 6.9, *J* = 1.3 Hz), 7.59 (ddd, 1 H, *J* = 8.3, *J* = 7.0, *J* = 1.2 Hz), 7.53 (d, 1 H, *J* = 8.8 Hz), 7.43 (d, 1 H, *J* = 2.9 Hz), 7.06 (d, 1 H, *J* = 8.8 Hz), 6.81 (dd, 1 H, *J* = 8.8, *J* = 2.9 Hz), 3.82 (s, 3 H), 3.74 (s, 3 H); ^13^C-NMR (DMSO-*d*_6_), δ: 168.20, 158.43, 152.92, 146.22, 135.97, 128.90, 127.58, 126.54, 125.90, 124.84, 123.62, 123.09, 118.42, 112.35, 111.51, 110.69, 108.95, 56.24, 55.48; HR-MS: [M-H]^+^ calculated 322.10739 m/z, found 322.10892 m/z.

*N*-(3,5-dimethoxyphenyl)−1-hydroxynaphthalene-2-carboxamide (**2e**). Yield 83%; Mp. 118–121 °C; HPLC purity 96.95%; IR (cm^−1^): 3266, 2999, 2936, 2833, 2540, 1614, 1595, 1549, 1514, 1470, 1453, 1423, 1332, 1296, 1257, 1227, 1194, 1154, 1064, 985, 846, 813, 799, 711; ^1^H-NMR (DMSO-*d*_6_), δ: 13.93 (s, 1 H), 10.35 (s, 1 H), 8.31 (d, 1 H, *J* = 8.2 Hz), 8.11 (d, 1 H, *J* = 9.2 Hz), 7.91 (d, 1 H, *J* = 8.2 Hz), 7.67 (ddd, 1 H, *J* = 8.0, *J* = 6.8, *J* = 1.3 Hz), 7.58 (ddd, 1 H, *J* = 8.3, *J* = 7.0, *J* = 1.2 Hz), 7.47 (d, 1 H, *J* = 8.9 Hz), 7.05 (d, 2 H, *J* = 2.3 Hz), 6.36 (t, 1 H, *J* = 2.3 Hz), 3.77 (s, 6 H); ^13^C-NMR (DMSO-*d*_6_), δ: 169.49, 160.37, 159.91, 139.34, 135.99, 129.14, 127.47, 125.93, 124.65, 123.07, 123.01, 117.79, 107.60, 100.13, 96.69, 55.22; HR-MS: [M-H]^+^ calculated 322.10738 m/z, found 322.10788 m/z.

Other experimental data for the compounds that were synthesized in this study can be found in the supplementary information.

### Cell Culture

The human colon cancer cell line HCT116 wild type was obtained from ATCC and the normal human fibroblast cell lines NHDF were obtained from PromoCell. The human colon cancer cell line HCT116 with a p53 deletion (p53^−/−^) was kindly provided by Prof. M. Rusin from the Maria Sklodowska-Curie Memorial Cancer Centre and Institute of Oncology in Gliwice, Poland^89^. The cells were grown as monolayer cultures in Dulbecco’s modified Eagle’s medium with the antibiotic gentamicin (200 μL/100 mL medium) in 75 cm^2^ flasks (Nunc). The DMEM for the HCT116 were supplemented with 12% heat-inactivated fetal bovine serum (Sigma-Aldrich, St. Louis, MO, USA) and for NHDF with 15% non-inactivated fetal bovine serum (Sigma Aldrich, St. Louis, MO, USA). The cells were cultured under standard conditions at 37 °C in a humidified atmosphere at 5% CO_2_.

### Cytotoxicity studies

The cells were seeded in 96-well plates (Nunc) at a density of 5,000 cells/well (HCT116) and 4,000 cells/well (NHDF) and incubated at 37 °C for 24 h. The assay was performed following a 72 h incubation with varying concentrations of the compounds that were being tested. Then, 20 µL of CellTiter 96^®^ AQ_ueous_ One Solution-MTS (Promega) was added to each well (with 100 µL DMEM without phenol red) and incubated for 1 h at 37 °C. The optical densities of the samples were analyzed at 490 nm using a Synergy 4 multi-plate reader (BioTek, Winooski, VT, USA). The results are expressed as the percentage of the control and were calculated as the inhibitory concentration (IC_50_) values (using a GraphPad Prism 7). The IC_50_ parameter was defined as the compound concentration that was necessary to reduce the proliferation of cells to 50% of the untreated control. Each individual compound was tested in triplicate in a single experiment with each experiment being repeated three or four times.

### Apoptosis assay

The dual acridine orange/ethidium bromide (AO/EB) staining method was used for the morphological analysis of apoptosis. The cells were seeded on coverslips at a density of 8·10^5^ cells/slide and incubated at 37 °C for 48 h. Then, the medium was removed and solutions of **5 s**, **5 u**, **7 f** and **DOX** at the IC_50_ concentration were added. After 24 h, the cells were washed with PBS, and then the cells were incubated in a staining solution (100 μg/mL AO and 100 μg/mL EB in PBS) (Sigma-Aldrich, St. Louis, MO, USA) for 3–4 min. After staining, the coverslips were washed with PBS and fixed with 3.7% paraformaldehyde for 15 min. Observations and photography were performed with a Nikon Eclipse Ni-U fluorescent microscope equipped with the sets of filters for AO and EB (excitation filter 450–490, barrier filter BA520).

### Immunoblotting

The HCT116 cells were seeded in 3-cm Petri dishes (Nunc) at a density of 0.5·10^6^ cells/well and incubated overnight. The next day, solutions of anilides (ten-fold IC_50_ concentration) and **DOX** (2.5 μM) were added and the cells were incubated for 12 or 24 h. The cells were harvested by trypsinization and washed with cold PBS. Next, the cells were centrifuged and suspended in an RIPA buffer (Thermo-Fisher Scientific) containing Halt Protease Inhibitor Cocktail (Thermo-Fisher Scientific), Halt Phosphatase Inhibitor Cocktail (Thermo-Fisher Scientific) along with 0.5 M EDTA and lysed for 20 min on ice. Then, the lysates were sonicated, centrifuged at 10,000 rpm for 10 min at 4 °C and the supernatants were collected for further analysis. The protein concentration was determined using a Micro BCA™ Protein Assay Kit (Thermo-Fisher Scientific, Waltham, MA, USA c) according to the manufacturer’s instructions. Equal amounts of the proteins (20 μg) were electrophoresed on SDS-Page gels and transferred onto nitrocellulose membranes. The membranes were blocked in 5% non-fat milk prepared in PBS containing 0.1% Tween-20 (TPBS) for 1 h. After blocking, the membranes were incubated with specific primary antibodies: p21^Waf1/Cip1^, p53, cytochrome c, caspase-8, caspase-9, AIF, PARP, GAPDH and β-Actin overnight at 4 °C, and then washed and incubated with horseradish peroxidase (HRP)-conjugated secondary antibodies for 1 h at room temperature. All of the antibodies were purchased from CellSignaling (Danvers, MA, USA) and were diluted 1:1000 in 5% milk in TPBS. Finally, the membranes were washed and incubated with a SuperSignal™ West Pico Chemiluminescent Substrate (Thermo-Fisher Scientific). The chemiluminescence signals were captured using a ChemiDoc™ XRS+ System (Bio-Rad Laboratories, Hercules, CA, USA). The experiments were performed at least three times. Densitometric analysis was performed using ImageJ 1.41 software (Wayne Rasband, National Institutes of Health, USA).

### Intercalation

For the DNA binding studies, Calf-thymus DNA (CT-DNA) was purchased from Sigma- Aldrich. The lyophilized CT-DNA was dissolved in 10 mM Tris-HCl, pH 7.9, mixed gently and left overnight at 4 °C. The purity of the CT-DNA solution was determined by measuring the ratio of UV absorbance at 260 and 280 nm. A ratio of more than 1.8 indicated that the DNA was sufficiently free of proteins. Then, the concentration of CT-DNA was determined from the absorbance at 260 nm using an extinction coefficient of 6600 M^−1^cm^−1^. The tested compounds including **DOX** and **CP-31398** were dissolved to a concentration of 8.35 mM in DMSO, which was then used as the stock solution for preparing the various concentrations (25, 12.5, 6 and 3 µM) in 1 mL in 10 mM of Tris-HCl (pH 7.9). Afterwards, 18 µM CT-DNA was added to the prepared solutions, which were incubated for 1.5 h at 37 °C with occasional vortexing. The absorption spectra were measured using a Hitachi U-2900 spectrophotometer in the range of 200–500 nm. All absorption spectra were imported into OriginPro 8.0 and compared.

### CoMFA and CoMSA approaches

Comparative molecular field analysis (CoMFA) is a widely used procedure for simulating the influence of the molecular shape when modeling steric (Lennard-Jones) and electrostatic (Coulomb) fields as being important intermolecular interactions that are involved in non-covalent ligand-receptor binding^90^. Generally, the CoMFA approach is based on the assumption that a comparative analysis of the 3D patterns that are produced within the cubic mesh of points that encompasses aligned molecules using suitable probes can account for the differences in the binding affinities or the biological activity profiles for a congeneric series of compounds. In fact, the modeling efficiency of the electronic and steric potentials in the molecular environment is strictly dependent on the selection of a suitable atomic probe (usually z positively charged sp^3^ carbon atom) and the superimposition that is applied following the putative pharmacophore hypothesis. As a result, the distribution of the potential values on the mesh points relies on the molecular coordinates, which are valid factors that control the value of the atomic partial charges. The resulting potential energies at each lattice point can be illustrated graphically as a three-dimensional color contour pattern that indicates the spatial areas, where a steric hindrance and/or charged substituents enhances or diminishes the binding affinity^91^.

A self-organizing neural network (SOM) data processing was performed as described earlier^92^.

### PCA and PLS procedures

The partial least squares (PLS) method produces a relationship between variable Y and an ensemble of descriptors **X**, which are expressed in the form of the equation:

1 $${\rm{Y}}={\bf{X}}\,b+{\rm{e}}$$

where *b* = *a* vector of the regression coefficients and *e* = *a* vector of the errors.

The generated data are centered and processed by the PLS analysis by constructing models the complexity of which is estimated using the leave-one-out cross-validation procedure (CV). In the leave-one-out CV, one repeats the calibration *m* times, each time treating the *i*-th left-out object as the prediction object^93^.

A cross-validated leave-one-out q^2^_CV_ value to estimate the performance of a model is computed using the following formula:

2 $${q}_{CV}^{2}=1-\frac{{\sum }_{i}^{m}{(ob{s}_{i}-pre{d}_{i})}^{2}}{{\sum }_{i}^{m}{(ob{s}_{i}-mean(ob{s}_{i}))}^{2}}$$

where *obs* is the assayed value; *pred* - predicted value; *mean* - mean value of *obs* and *i* refers to the object index.

The quality of external predictions is measured by the standard deviation of error of prediction (*SDEP*) and q^2^_test_ defined as:

3 $$SDEP=\sqrt{\frac{{\sum }_{i}^{n}{(pre{d}_{i}-ob{s}_{i})}^{2}}{n}}$$

4 $${q}_{test}^{2}=1-\frac{{\sum }_{i}^{n}{(ob{s}_{i}-pre{d}_{i})}^{2}}{{\sum }_{i}^{n}{(ob{s}_{i}-mean(ob{s}_{i}))}^{2}}$$

The uninformative variable elimination (UVE-PLS) as well as its modifications, namely, modified UVE (m-UVE) and iterative variable elimination (IVE-PLS) have been applied in both QSAR and QSPR schemes, respectively^94^. The original UVE algorithm, which was developed by Centner *et al*., analyzes the reliability of the *mean(b)/s(b)* ratio, where *s(b)* is the standard deviation of regression coefficient *b*, which is calculated using the PLS method^95^. Then, only variables with relatively high stability values are included in the final PLS model. In the current calculations, the iterative IVE-PLS procedure was used as a modification of the single-step UVE algorithm in order to identify the variables to be eliminated. Basically, the entire algorithm is composed of the following stages:

*Step 1*. Standard PLS analysis with LOO-CV to evaluate the performance of the PLS model (q^2^_CV_).

*Step 2*. Elimination of the matrix column with the lowest *abs(mean(b)/std(b))* value.

*Step 3*. Standard PLS analysis of the new matrix without the column that was eliminated in step 2.

*Step 4*. Iterative repetition of the steps 1 to 3 to maximize the LOO q^2^_CV_ parameter.

### Model builder

All of the pharmacological data were specified by the same laboratory in order to eradicate any potential data noise introduced by pooling the data sets from various sources. The *in vitro* efficiency of the anticancer hydroxynaphthanilides is presented in Table 1. The constitution of the molecular models was generated using the CACTVS/csed molecular editor. The spatial geometry of the molecules was formed with the CORINA 3D. The (inter)change file format converter OpenBabel was used in file preparation.

### Molecular modeling

The modeling were performed using the Sybyl-X 2.0/Certara software package running on an HP workstation with a Debian 6.0 operating system. The initial geometry for all compounds was generated using the MAXMIN2 module in which the standard Tripos force field (POWELL conjugate gradient algorithm) with 0.01 kcal/mol energy gradient convergence criterion and a distant dependent dielectric constant were applied. The Gasteiger-Hückel procedure, which was implemented in Sybyl to calculate the electrostatic potential, was initially used to calculate the partial atomic charges. The one 14-ordered atom trial alignment on molecule **1** was selected to cover the entire bonding topology in the maximal common structure (1-hydroxynaphthalene and peptide-like motif) using the atom FIT method as is presented in Table 1. The sp^3^ hybridized carbon probe atom with a charge of +1 and 0 and hydrogen as the probe atom with a charge of +1 were used to calculate the electrostatic and steric fields, respectively. The CoMFA grid spacing was 2.0 Å for all of the Cartesian dimensions within the defined region of the 3D lattice, which extended beyond the van der Waals envelopes of all of the molecules by at least 4.0 Å. The non-covalent interaction fields were specified at each intersection on a regularly spaced grid of points.

For each molecule, the energies with a total of 864 grid points were calculated with 2 Å spacing in a 12 × 9 × 8 lattice. All of the columns with an energy variance of less than 2.0 kcal/mol were discarded by setting the sigma parameter to 2.0 kcal/mol in order to reduce any data noise. Both the steric/electrostatic energies with a value greater than 30.0 kcal/mol were truncated to a tentative value of 30.0 (default cut-off). The SAMPLS method using the standard internal and external validation techniques was used to determine the statistical index of the predictive power of a model describing the variations in the CoMFA interaction fields (independent variables) and changes in activity (dependent variable).

The SONNIA software was used in the CoMSA analysis to simulate 20 × 20 or 30 × 30 SOMs with a winning distance (md) that varied within the range of 0.2 to 2.0. The Cartesian coordinates of the molecular surfaces for the superimposed molecules were preceded by the SOM network to form a two-dimensional map of the electrostatic potential – the most active analog (**8c**) was also used to form the template molecule.

The output maps were subsequently transformed into a 400- or 900-element vector, which was processed using the PLS method implemented in the MATLAB programming environment corresponding to the CoMFA analysis.

## Supplementary information

Supplementray information

## Data Availability

The datasets that were generated and/or analyzed during the current study are available from the corresponding author on reasonable request.
