# Supplementary material for: Design and synthesis of anticancer 1-hydroxynaphthalene-2-carboxanilides with a p53 independent mechanism of action
Source: Sci Rep. 2019 Apr 23;9:6387. doi: 10.1038/s41598-019-42595-y (PMC6476888; doi:10.1038/s41598-019-42595-y)
Supplement: Supplementary file 1 — Supplementray information [file 41598_2019_42595_MOESM1_ESM.pdf]

Supplementary information materials

Design and synthesis of anticancer 1-hydroxynaphthalene-2-carboxanilides with a p53 independent mechanism of action

Ewelina Spaczyńska[1], Anna Mrozek-Wilczkiewicz[2], Katarzyna Malarz[2], Jiri Kos[3], Tomas Gonec[4], Michal Oravec[5], Robert Gawecki[2], Andrzej Bak[1], Jana Dohanosova[6], Iva Kapustikova[3], Tibor Liptaj[6], Josef Jampilek[3]*, Robert Musiol[1]*

[1]Institute of Chemistry, University of Silesia, 75 Pułku Piechoty 1a, 41-500 Chorzów, Poland
[2]A. Chełkowski Institute of Physics and Silesian Center for Education and Interdisciplinary Research, University of Silesia, 75 Pułku Piechoty 1a, 41-500 Chorzów, Poland
[3]Department of Pharmaceutical Chemistry, Faculty of Pharmacy, Comenius University, Odbojarov 10, 832 32 Bratislava, Slovakia;
[4]Department of Chemical Drugs, Faculty of Pharmacy, University of Veterinary and Pharmaceutical Sciences, Palackeho 1, Brno 612 42, Czech Republic;
[5]Global Change Research Institute CAS, Belidla 986/4a, Brno 603 00, Czech Republic;
[6]Central Laboratories, Faculty of Chemical and Food Technology, Slovak University of Technology in Bratislava, Radlinskeho 9, Bratislava 81237, Slovakia

1-hydroxy-*N*-(3,4,5-trimethoxyphenyl)naphthalene-2-carboxamide (**2f**). Yield 66%; Mp. 177-180°C; HPLC purity 98.47%; IR (cm^{-1}): 3375, 1627, 1594, 1549, 1504, 1452, 1410, 1393, 1328, 1293, 1278, 1230, 1210, 1186, 1127, 1001, 989, 950, 836, 825, 814, 793, 760, 725; ^{1}H-NMR (DMSO-d_6), δ: 14.01 (s, 1H), 10.36 (s, 1H), 8.31 (d, *J* = 8.1 Hz, 1H), 8.11 (d, *J* = 8.8 Hz, 1H), 7.92 (d, *J* = 8.1 Hz, 1H), 7.68 (t, *J* = 7.2 Hz, 1H), 7.56-7.61 (m, 1H), 7.47 (d, *J* = 8.8 Hz, 1H), 7.17 (s, 2H), 3.81 (s, 6H), 3.67 (s, 3H); ^{13}C-NMR (DMSO-d_6), δ: 169.29, 159.93, 152.63, 135.97, 134.56, 133.64, 129.13, 127.49, 125.94, 124.68, 123.08, 122.92, 117.78, 107.53, 99.84, 60.12, 55.86; HR-MS: for $C_{20}H_{19}NO_5$ [M+H]$^+$ calculated 354.133599 *m/z* found 354.13376 *m/z*.

1-hydroxy-*N*-(2-methoxy-4-nitrophenyl)naphthalene-2-carboxamide (**2g**). Yield 69%; Mp. 250-253°C; HPLC purity 99.04%; IR (cm^{-1}): 3422, 1629, 1589, 1550, 1513, 1485, 1457, 1414, 1391, 1362, 1333, 1303, 1267, 13241, 1209, 1174, 1152, 1123, 1091, 1024, 941, 860, 799, 788, 764, 740, 713; ^{1}H-NMR (DMSO-d_6), δ: 12.80 (br. s, 1H), 11.00 (s, 1H), 8.42 (d, *J* = 8.8 Hz, 1H), 8.37 (d, *J* = 8.1 Hz, 1H), 8.08 (d, *J* = 8.8 Hz, 1H), 7.97 (dd, *J* = 9.0, 2.1 Hz, 1H), 7.94 (d, *J* = 8.3 Hz, 1H), 7.90-7.92 (m, 1H), 7.65-7.70 (m, 1H), 7.59-7.64 (m, 1H), 7.54 (d, *J* = 8.8 Hz, 1H), 4.07 (s, 3H); ^{13}C-NMR (DMSO-d_6), δ: 166.72, 156.60, 149.95, 143.69, 136.23, 133.53, 128.99, 127.75, 126.05, 125.01, 124.58, 123.23, 121.53, 119.42, 116.82, 111.06, 106.16, 56.78; HR-MS: for $C_{18}H_{14}N_2O_5$ [M-H]$^+$ calculated 337.081898 *m/z* found 337.08331 *m/z*.

1-hydroxy-*N*-(2-methoxy-5-nitrophenyl)naphthalene-2-carboxamide (**2h**). Yield 68%; Mp. 215-218°C; HPLC purity 99.24%; IR (cm^{-1}): 3425, 1634, 1616, 1593, 1541, 1511, 1485, 1428, 1391, 1357, 1335, 1266, 1241, 1205, 1178, 1151, 1136, 1091, 1075, 1014, 895, 819, 800, 786, 765,

744, 719; ^{1}H-NMR (DMSO-d_6), δ: 13.14 (br. s, 1H), 10.72 (s, 1H), 8.87 (d, *J* = 2.8 Hz, 1H), 8.35 (d, *J* = 8.3 Hz, 1H), 8.17 (dd, *J* = 9.1, 2.8 Hz, 1H), 8.08 (d, *J* = 8.8 Hz, 1H), 7.93 (d, *J* = 8.1 Hz, 1H), 7.65-7.70 (m, 1H), 7.58-7.63 (m, 1H), 7.52 (d, *J* = 8.8 Hz, 1H), 7.37 (d, *J* = 9.3 Hz, 1H), 4.05 (s, 3H); ^{13}C-NMR (DMSO-d_6), δ: 167.67, 157.58, 156.41, 140.36, 136.14, 129.00, 127.68, 126.77, 126.00, 124.88, 124.06, 123.17, 122.01, 118.94, 118.75, 111.49, 109.77, 57.01; HR-MS: for $C_{18}H_{14}N_2O_5$ [M-H]$^+$ calculated 337.081898 *m/z* found 337.08313 *m/z*.

1-hydroxy-*N*-[2-methoxy-5-(trifluoromethyl)phenyl]naphthalene-2-carboxamide. (**2i**). Yield 72%; Mp. 158-160°C; HPLC purity 93.67%; IR (cm^{-1}): 3429, 1621, 1594, 1548, 1489, 1459, 1439, 1412, 1390, 1350, 1313, 1276, 1264, 1241, 1207, 1160, 1108, 1074, 1024, 943, 921, 891, 849, 799, 785, 759, 720; ^{1}H-NMR (DMSO-d_6), δ: 13.35 (br. s, 1H), 10.58 (s, 1H), 8.34 (d, *J* = 8.3 Hz, 1H), 8.22 (s, 1H), 8.08 (d, *J* = 8.8 Hz, 1H), 7.93 (d, *J* = 8.1 Hz, 1H), 7.68 (t, *J* = 7.5 Hz, 1H), 7.57-7.64 (m, 2H), 7.50 (d, *J* = 8.8 Hz, 1H), 7.34 (d, *J* = 8.8 Hz, 1H), 3.97 (s, 3H); ^{13}C-NMR (DMSO-d_6), δ: 168.09, 158.05, 154.47, 136.06, 128.94, 127.60, 126.65, 125.93, 124.84, 124.33 (q, *J* = 271.5 Hz), 123.79, 123.42 (q, *J* = 4.2 Hz), 123.12, 121.28 (q, *J* = 4.1 Hz), 120.88 (q, *J* = 32.2 Hz), 118.67, 111.92, 109.24, 56.36; HR-MS: for $C_{19}H_{14}F_3NO_3$ [M-H]$^+$ calculated 360.084204 *m/z* found 360.08572 *m/z*.

1-hydroxy-*N*-(2-methoxy-5-methylphenyl)naphthalene-2-carboxamide (**2j**). Yield 74%; Mp. 106-108°C; HPLC purity 99.62%; IR (cm^{-1}): 3433, 1627, 1592, 1539, 1488, 1436, 1411, 1387, 1361, 1322, 1285, 1275, 1249, 1207, 1172, 1149, 1120, 1033, 1022, 938, 874, 800, 790, 759, 726 ; ^{1}H-NMR (DMSO-d_6), δ: 13.92 (s, 1H), 10.23 (s, 1H), 8.31 (d, *J* = 8.1 Hz, 1H), 8.07 (d, *J* = 8.8 Hz, 1H), 7.91 (d, *J* = 8.3 Hz, 1H), 7.66 (ddd, *J* = 8.2, 6.9, 1.4 Hz, 1H), 7.56-7.60 (m, 1H), 7.49 (d, *J* = 1.8 Hz, 1H), 7.46 (d, *J* = 8.8 Hz, 1H), 7.01-7.08 (m, 2H), 3.82 (s, 3H), 2.29 (s, 3H); ^{13}C-NMR (DMSO-d_6), δ: 168.74, 159.06, 150.54, 135.99, 129.16, 128.98, 127.61, 127.15, 126.59, 125.93, 125.23, 124.84, 123.42, 123.12, 118.22, 111.54, 108.34, 55.79, 20.24; HR-MS: for $C_{19}H_{17}NO_3$ [M+H]$^+$ calculated 308.12812 *m/z* found 308.12842 *m/z*.

1-hydroxy-*N*-(2-methoxy-6-methylphenyl]naphthalene-2-carboxamide (**2k**). Yield 58%; Mp. 177-179°C; HPLC purity 99.96%; IR (cm^{-1}): 3400, 1616, 1595, 1575, 1511, 1498, 1472, 1412, 1389, 1328, 1309, 1267, 1252, 1208, 1147, 1080, 1033, 1023, 946, 846, 802, 791, 771, 759, 730, 720; ^{1}H-NMR (DMSO-d_6), δ: 14.40 (s, 1H), 10.07 (s, 1H), 8.29 (d, *J* = 8.2 Hz, 1H), 8.12 (d, *J* = 8.8 Hz, 1H), 7.91 (d, *J* = 8.3 Hz, 1H), 7.65-7.69 (m, 1H), 7.55-7.60 (m, 1H), 7.45 (d, *J* = 8.8 Hz, 1H), 7.25 (t, *J* = 8.0 Hz, 1H), 6.97 (d, *J* = 8.1 Hz, 1H), 6.92 (d, *J* = 7.6 Hz, 1H), 3.77 (s, 3H), 2.20 (s,

3H); ^{13}C-NMR (DMSO-d_6), δ: 169.80, 160.06, 155.24, 137.03, 135.93, 129.00, 128.01, 127.50, 125.87, 124.71, 123.73, 123.01, 122.98, 121.98, 117.77, 109.31, 106.96, 55.60, 17.68; HR-MS: for $C_{19}H_{17}NO_3$ $[M+H]^+$ calculated 308.12812 *m/z* found 308.12842 *m/z*.

N-(2,5-dimethylphenyl)-1-hydroxynaphthalene-2-carboxamide (**3d**). Yield 70%; Mp. 92-95°C; HPLC purity 95.71%; IR (cm^{-1}): 3445, 1627, 1575, 1538, 1447, 1410, 1387, 1358, 1330, 1286, 1246, 1209, 1025, 882, 840, 805, 791, 757; ^{1}H-NMR (DMSO-d_6), δ: 14.28 (br. s, 1H), 10.34 (s, 1H), 8.30 (dd, 1H, *J*=7.7, *J*=1.3, Hz), 8.10 (d, 1H, *J*=8.6 Hz), 7.91 (d, 1H, *J*=8.1 Hz), 7.67 (ddd, 1H, *J*=8.2, *J*=6.9, *J*=1.3 Hz), 7.58 (ddd, 1H, *J*=8.3, *J*=7.0, *J*=1.2 Hz), 7.46 (d, 1H, *J*=8.9 Hz), 7.20 (d, 1H, *J*=8.1 Hz), 7.17 (s, 1H), 7.06 (dd, 1H, *J*=7.8, *J*=1.3, Hz), 2.31 (s, 3H), 2.21 (s, 3H); ^{13}C-NMR (DMSO-d_6), δ: 169.57, 159.96, 135.96, 135.35, 134.93, 131.22, 130.22, 129.02, 127.76, 127.51, 127.50, 125.88, 124.70, 123.03, 122.94, 117.87, 107.16, 20.41, 17.35; HR-MS: $[M-H]^+$ calculated 290.11756 m/z, found 290.11859 m/z.

N-(2,6-dimethylphenyl)-1-hydroxynaphthalene-2-carboxamide (**3e**). Yield 80%; Mp. 102-105°C; HPLC purity 98.10%; IR (cm^{-1}): 3413, 1615, 1587, 1577, 1508, 1493, 1408, 1379, 1325, 1274, 1247, 1203, 1147, 950, 877, 817, 795, 773, 765, 727, 714; ^{1}H-NMR (DMSO-d_6), δ: 14.36 (s, 1H), 10.26 (s, 1H), 8.29 (dd, 1H, *J*=8.3, *J*=1.0 Hz), 8.13 (d, 1H, *J*=8.9 Hz), 7.92 (d, 1H, *J*=8.2 Hz), 7.67 (ddd, 1H, *J*=8.2, *J*=6.9, *J*=1.3 Hz), 7.57 (ddd, 1H, *J*=8.2, *J*=6.9, *J*=1.3 Hz), 7.46 (d, 1H, *J*=8.8 Hz), 7.18 (s, 3H), 2.22 (s, 6H); ^{13}C-NMR (DMSO-d_6), δ: 169.57, 160.10, 135.96, 135.69, 134.09, 129.04, 127.87, 127.51, 127.25, 125.91, 124.71, 123.00, 122.76, 117.90, 106.78, 17.92; HR-MS: $[M-H]^+$ calculated 290.11756 m/z, found 290.11847 m/z.

N-(3,5-dimethylphenyl)-1-hydroxynaphthalene-2-carboxamide (**3f**). Yield 69%; Mp. 88-90°C; HPLC purity 94.30%; IR (cm^{-1}): 3420, 1614, 1579, 1543, 1465, 1412, 1388, 1355, 1327, 1280, 1243, 1206, 1183, 864, 840, 803, 755, 726, 685; ^{1}H-NMR (DMSO-d_6), δ: 14.10 (br. s, 1H), 10.33 (s, 1H), 8.31 (d, 1H, *J*=8.2 Hz), 8.12 (d, 1H, *J*=8.9 Hz), 7.91 (d, 1H, *J*=8.2 Hz), 7.67 (ddd, 1H, *J*=8.2, *J*=6.9, *J*=1.3 Hz), 7.58 (ddd, 1H, *J*=8.2, *J*=6.9, *J*=1.3 Hz), 7.45 (d, 1H, *J*=8.9 Hz), 7.37 (s, 2H), 6.84 (s, 1H), 2.30 (s, 6H); ^{13}C-NMR (DMSO-d_6), δ: 169.37, 159.96, 137.66, 137.37, 135.94, 129.07, 127.44, 126.26, 125.87, 124.65, 123.04, 123.01, 119.84, 117.75, 107.51, 21.00; HR-MS: $[M-H]^+$ calculated 290.11756 m/z, found 290.11917 m/z.

1-hydroxy-*N*-(2,4,6-trimethylphenyl)naphthalene-2-carboxamide (**3g**). Yield 52%; Mp. 160-163°C; HPLC purity 100%; IR (cm^{-1}): 3306, 1616, 1590, 1520, 1498, 1456, 1409, 1383, 1320, 1277, 1255, 1210, 1141, 1081, 1033, 1021, 946, 933, 844, 799, 765, 716, 708, 667; ^{1}H-NMR (DMSO-d_6), δ: 14.40 (s, 1H), 10.15 (s, 1H), 8.29 (d, *J* = 8.1 Hz, 1H), 8.12 (d, *J* = 8.8 Hz, 1H), 7.91 (d, *J* = 8.3 Hz, 1H), 7.67 (t, *J* = 7.5 Hz, 1H), 7.55-7.60 (m, 1H), 7.45 (d, *J* = 8.8 Hz, 1H), 6.98 (s, 2H), 2.28 (s, 3H), 2.17 (s, 6H); ^{13}C-NMR (DMSO-d_6), δ: 169.66, 160.09, 136.29, 135.94, 135.30, 131.44, 129.00, 128.47, 127.50, 125.88, 124.74, 123.00, 122.78, 117.85, 106.83, 20.54, 17.86; HR-MS: for $C_{20}H_{19}NO_2$ $[M-H]^+$ calculated 304.133205 *m/z* found 304.13416 *m/z.*

1-hydroxy-*N*-(5-methoxy-2-methylphenyl)naphthalene-2-carboxamide (**3h**). Yield 60%; Mp. 128-130°C; HPLC purity 98.88%; IR (cm^{-1}): 3317, 1608, 1593, 1575, 1521, 1498, 1464, 1409, 1377, 1320, 1279, 1251, 1208, 1196, 1176, 1156, 1110, 1033, 1021, 962, 954, 868, 841, 807, 791, 757, 733, 712, 897; ^{1}H NMR (DMSO-d_6) δ 14.19 (s, 1H), 10.37 (s, 1H), 8.31 (d, *J* = 8.3 Hz, 1H), 8.10 (d, *J* = 8.9 Hz, 1H), 7.92 (d, *J* = 8.1 Hz, 1H), 7.68 (t, *J* = 7.4 Hz, 1H), 7.59 (t, *J* = 7.5 Hz, 1H), 7.47 (d, *J* = 8.8 Hz, 1H), 7.23 (d, *J* = 8.4 Hz, 1H), 6.98 (d, *J* = 2.2 Hz, 1H), 6.85 (dd, *J* = 8.4, 2.4 Hz, 1H), 3.76 (s, 3H), 2.19 (s, 3H); ^{13}C-NMR (DMSO-d_6), δ: 169.47, 159.88, 157.57, 135.97, 135.92, 130.93, 129.04, 127.52, 126.06, 125.90, 124.70, 123.05, 123.02, 117.90, 112.67, 112.53, 107.27, 55.23, 16.94; HR-MS: for $C_{19}H_{17}NO_3$ $[M+H]^+$ calculated 308.12812 *m/z* found 308.12842 *m/z.*

1-hydroxy-*N*-[2-methyl-5-(trifluoromethyl)phenyl]naphthalene-2-carboxamide (**3i**). Yield 24%; Mp. 140-143°C; HPLC purity 98.35%; IR (cm^{-1}): 3462, 1634, 1589, 1554, 1451, 1425, 1408, 1389, 1375, 1330, 1304, 1269, 1231, 1205, 1173, 1161, 1153, 1107, 1073, 1033, 1024, 996, 940, 918, 893, 819, 797, 786, 760, 743, 719; ^{1}H-NMR (DMSO-d_6), δ: 13.87 (s, 1H), 10.54 (s, 1H), 8.31 (d, *J* = 8.3 Hz, 1H), 8.09 (d, *J* = 8.8 Hz, 1H), 7.93 (d, *J* = 8.1 Hz, 1H), 7.80 (s, 1H), 7.68 (t, *J* = 7.5 Hz, 1H), 7.56-7.62 (m, 3H), 7.49 (d, *J* = 8.8 Hz, 1H), 2.36 (s, 3H); ^{13}C-NMR (DMSO-d_6), δ: 169.58, 159.75, 139.24, 136.10, 136.07, 131.51, 129.17, 127.56, 127.11 (q, *J* = 31.8 Hz), 126.00, 124.68, 124.09 (q, *J* = 272.5 Hz), 123.67 (q, *J* = 3.6 Hz), 123.13 (q, *J* = 3.6 Hz), 123.11, 123.09, 118.12, 107.34, 17.89; HR-MS: for $C_{19}H_{14}F_3NO_2$ $[M-H]^+$ calculated 344.08929 *m/z* found 344.08994 *m/z.*

N-(2,4-difluorophenyl)-1-hydroxynaphthalene-2-carboxamide (**4d**). Yield 70%; Mp. 185-187°C; HPLC purity 96.34%; IR (cm^{-1}): 3444, 1634, 1622, 1596, 1538, 1498, 1470, 1429, 1411, 1393, 1342, 1309, 1271, 1244, 1192, 1147, 1095, 965, 935, 843, 803, 784, 756, 724, 716; ^{1}H-NMR (DMSO-d_6), δ: 13.86 (s, 1H), 10.57 (s, 1H), 8.31 (d, 1H, *J*=8.6 Hz), 8.06 (d, 1H, *J*=8.9 Hz), 7.92 (d, 1H, *J*=8.2 Hz), 7.68 (ddd, 1H, *J*=8.1, *J*=7.0, *J*=1.0 Hz), 7.57-7.64 (m, 2H), 7.48 (d, 1H, *J*=8.9 Hz), 7.43 (ddd, 1H, *J*=10.4, *J*=9.1, *J*=2.8 Hz), 7.16-7.20 (m, 1H); ^{13}C-NMR (DMSO-d_6), δ: 169.74, 160.37 (dd, *J*=245.7, 11.6 Hz), 159.86, 156.66 (dd, *J*=250.1, 12.8 Hz), 136.09, 129.41 (dd, *J*=9.9, 2.5 Hz), 129.25, 127.56, 126.03, 124.60, 123.11, 122.97, 120.95 (dd, *J*=12.8, 3.7 Hz), 118.15, 111.49 (dd, *J*=22.2, 3.6 Hz), 106.96, 104.58 (dd, *J*=26.8, 24.5 Hz); ^{19}F-NMR (DMSO-d_6), δ: -111.91 (m), -115.56 (m); HR-MS: $[M-H]^+$ calculated 298.06741 m/z, found 298.06833 m/z.

N-(2,5-difluorophenyl)-1-hydroxynaphthalene-2-carboxamide (**4e**). Yield 72%; Mp. 153-155°C; HPLC purity 98.26%; IR (cm^{-1}): 3443, 1647, 1625, 1538, 1480, 1441, 1412, 1392, 1318, 1272, 1241, 1208, 1172, 1157, 1092, 973, 866, 841, 798, 785, 758, 730; ^{1}H-NMR (DMSO-d_6), δ: 13.58 (br. s, 1H), 10.70 (s, 1H), 8.33 (d, 1H, *J*=8.6 Hz), 8.07 (d, 1H, *J*=8.9 Hz), 7.93 (d, 1H, *J*=8.2 Hz), 7.68 (ddd, 1H, *J*=8.2, *J*=6.9, *J*=1.3 Hz), 7.58-7.64 (m, 2H), 7.49 (d, 1H, *J*=8.9 Hz), 7.42 (td, 1H, *J*=9.5, *J*=4.9 Hz), 7.18-7.22 (m, 1H); ^{13}C-NMR (DMSO-d_6), δ: 169.10, 159.30, 157.63 (dd, *J*=240.0, 2.1 Hz), 152.22 (dd, *J*=243.3, 2.8 Hz), 136.15, 129.25, 127.60, 126.06, 125.83 (dd, *J*=14.6, 11.4 Hz), 124.64, 123.30, 123.14, 118.40, 116.91 (dd, *J*=22.9, 9.8 Hz), 113.95 (dd, *J*=26.6, 1.2 Hz), 113.71 (dd, *J*=24.0, 8.1 Hz), 107.69; ^{19}F-NMR (DMSO-d_6), δ: -117.66 (m), -126.56 (m); HR-MS: $[M-H]^+$ calculated 298.06741 m/z, found 298.06854 m/z.

N-(2,6-difluorophenyl)-1-hydroxynaphthalene-2-carboxamide (**4f**). Yield 76%; Mp. 197-200°C; HPLC purity 97.07%; IR (cm^{-1}): 3392, 1633, 1617, 1597, 1514, 1470, 1413, 1326, 1290, 1253, 1238, 1207, 1144, 1004, 944, 799, 788, 768, 730, 708; ^{1}H-NMR (DMSO-d_6), δ: 13.46 (br. s, 1H), 10.58 (s, 1H), 8.31 (d, 1H, *J*=8.1 Hz), 8.09 (d, 1H, *J*=8.8 Hz), 7.94 (d, 1H, *J*=8.1 Hz), 7.71 (ddd, 1H, *J*=8.2, *J*=6.9, *J*=1.3 Hz), 7.60 (ddd, 1H, *J*=8.3, *J*=7.0, *J*=1.2 Hz), 7.52-7.76 (m, 1H), 7.44 (d, 1H, *J*=6.6 Hz), 7.31 (d, 1H, *J*=8.1 Hz), 7.29 (t, 1H, *J*=8.1 Hz); ^{13}C-NMR (DMSO-d_6), δ: 170.08, 160.16, 158.08 (dd, *J*=249.1, 5.0 Hz), 136.18, 129.43, 129.20 (t, *J*=9.9 Hz), 127.60, 126.14, 124.55, 123.15, 122.74, 118.29, 113.50 (t, *J*=18.0 Hz), 112.33-111.86 (m), 106.38; HR-MS: $[M-H]^+$ calculated 298.06741 m/z, found 298.06821 m/z.

N-(3,4-difluorophenyl)-1-hydroxynaphthalene-2-carboxamide (**4g**). Yield 65%; Mp. 153-156°C; HPLC purity 95.00%; IR (cm^{-1}): 3433, 1624, 1596, 1577, 1538, 1514, 1504, 1469, 1441, 1412, 1388, 1329, 1277, 1243, 1202, 1149, 1120, 1083, 969, 872, 830, 818, 799, 786, 750, 716; ^{1}H-NMR (DMSO-d_6), δ: 13.73 (s, 1H), 10.59 (s, 1H), 8.31 (d, 1H, *J*=8.2 Hz), 8.07 (d, 1H, *J*=8.9 Hz), 7.87-7.92 (m, 2H), 7.67 (ddd, 1H, *J*=8.1, *J*=7.0, *J*=1.2 Hz), 7.55-7.60 (m, 2H), 7.45-7.50 (m, 2H); ^{13}C-NMR (DMSO-d_6), δ: 169.50, 159.91, 148.81 (dd, *J*=243.8, 13.3 Hz), 146.25 (dd, *J*=243.3, 12.6 Hz), 136.05, 134.67 (dd, *J*=8.9, 3.1 Hz), 129.26, 127.50, 126.01, 124.61, 123.09, 122.93, 118.42 (dd, *J*=6.2, 3.4 Hz), 117.96, 117.33 (d, *J*=17.8 Hz), 111.14 (d, *J*=21.3 Hz), 107.37; ^{19}F-NMR (DMSO-d_6), δ: -137.31 (ddd, *J*=23.2, 12.9, 8.9 Hz), -143,10 (m); HR-MS: $[M-H]^+$ calculated 298.06741 m/z, found 298.06851 m/z.

N-(3,5-difluorophenyl)-1-hydroxynaphthalene-2-carboxamide (**4h**). Yield 85%; Mp. 199-200°C; HPLC purity 96.14%; IR (cm^{-1}): 3438, 1643, 1623, 1607, 1576, 1543, 1466, 1416, 1386, 1338, 1305, 1277, 1247, 1176, 1157, 1118, 998, 924, 856, 827, 795, 783, 754, 726; ^{1}H-NMR (DMSO-d_6), δ: 13.51 (s, 1H), 10.66 (s, 1H), 8.30-8.33 (m, 1H), 8.05 (d, 1H, *J*=8.9 Hz), 7.92 (d, 1H, *J*=8.2 Hz), 7.68 (ddd, 1H, *J*=8.2, *J*=6.9, *J*=1.3 Hz), 7.59 (ddd, 1H, *J*=8.3, *J*=7.0, *J*=1.2 Hz), 7.56 (dd, 2H, *J*=9.4, *J*=2.1 Hz), 7.49 (d, 1H, *J*=8.9 Hz), 7.05 (tt, 1H, *J*=9.2, *J*=2.3 Hz); ^{13}C-NMR (DMSO-d_6), δ: 169.73, 162.24 (dd, *J*=243.4, 15.26 Hz), 159.87, 140.42 (t, *J*=13.7 Hz), 136.12, 129.39, 127.53, 126.09, 124.59, 123.13, 122.97, 118.08, 107.54, 104.46 (m), 100.67 (t, *J*=26.3 Hz); HR-MS: $[M-H]^+$ calculated 298.06741 m/z, found 298.06860 m/z.

1-hydroxy-*N*-(2,3,4-trifluorophenyl)naphthalene-2-carboxamide (**4i**). Yield 75%; Mp. 173-175°C; HPLC purity 95.58%; IR (cm^{-1}): 3447, 1626, 1557, 1511, 1472, 1394, 1307, 1286, 1251, 1204, 1150, 1092, 1039, 1023, 988, 919, 803, 788, 720, 687; ^{1}H-NMR (DMSO-d_6), δ: 13.69 (br. s, 1H), 10.74 (s, 1H), 8.31 (d, 1H, *J*=8.2 Hz), 8.05 (d, 1H, *J*=9.2 Hz), 7.93 (d, 1H, *J*=8.2 Hz), 7.69 (ddd, 1H, *J*=8.2, *J*=6.9, *J*=1.3 Hz), 7.60 (ddd, 1H, *J*=8.4, *J*=7.0, *J*=1.1 Hz), 7.49 (d, 1H, *J*=8.7 Hz), 7.38-7.46 (m, 2H); ^{13}C-NMR (DMSO-d_6), δ: 169.69, 159.87, 148.59 (ddd, *J*=246.8, 9.9, 2.3 Hz), 145.75 (ddd, *J*=251.2, 10.7, 3.6 Hz), 139.45 (ddd, *J*=248.4, 16.1, 14.5 Hz), 136.16, 129.37, 127.59, 126.11, 124.57, 123.14, 122.99, 122.48 (dd, *J*=7.9, 3.3 Hz), 122.39 (dd, *J*=9.9, 3.5 Hz), 118.28, 112.07 (dd, *J*=17.9, 3.7 Hz), 106.86; ^{19}F-NMR (DMSO-d_6), δ: -136.94-(-137.03) (m), -139.05 (dt, *J*=20.3, 5.9 Hz), -160.17-(-160.28) (m); HR-MS: $[M-H]^+$ calculated 316.05799 m/z, found 316.05899 m/z.

1-hydroxy-*N*-(2,4,5-trifluorophenyl)naphthalene-2-carboxamide (**4j**). Yield 73%; Mp. 201-204°C; HPLC purity 98.31%; IR (cm^{-1}): 3433, 1626, 1544, 1501, 1432, 1396, 1321, 1283, 1267, 1251, 1209, 1194, 1175, 1159, 1150, 1139, 1102, 1074, 898, 867, 805, 789, 757, 724, 700; ^{1}H-NMR (DMSO-d_6), δ: 13.64 (br. s, 1H), 10.67 (s, 1H), 8.31 (d, 1H, *J*=8.2 Hz), 8.05 (d, 1H, *J*=9.2 Hz), 7.92 (d, 1H, *J*=8.2 Hz), 7.79-7.87 (m, 1H), 7.75 (td, 1H, *J*=10.5, *J*=7.3 Hz), 7.66-7.71 (m, 1H), 7.57-7.62 (m, 1H), 7.49 (d, 1H, *J*=8.7 Hz); ^{13}C-NMR (DMSO-d_6), δ: 169.45, 159.61, 151.84 (ddd, *J*=246.7, 10.0, 2.5 Hz), 147.24 (ddd, *J*=247.5, 14.2, 12.1 Hz), 145.45 (ddd, *J*=242.4, 13.3, 3.4 Hz), 136.16, 129.32, 127.60, 126.09, 124.60, 123.14, 123.08, 121.22 (ddd, *J*=14.6, 8.8, 3.8 Hz), 118.34, 116.09 (dd, *J*=21.2, 1.7 Hz), 107.22, 106.26 (dd, *J*=26.8, 22.0 Hz); ^{19}F-NMR (DMSO-d_6), δ: -121.35 (dddd, *J*=14.1, 10.1, 7.3, 3.2 Hz), -136.64 (dddd, *J*=23.4, 11.0, 8.9, 3.1 Hz), -142.11 (dddd, *J*=23.3, 14.1, 11.5, 7.4 Hz); HR-MS: $[M-H]^+$ calculated 316.05799 m/z, found 316.05902 m/z.

1-hydroxy-*N*-(2,4,6-trifluorophenyl)naphthalene-2-carboxamide (**4k**). Yield 74%; Mp. 180-184°C; HPLC purity 95.51%; IR (cm^{-1}): 3396, 3073, 1615, 1597, 1524, 1515, 1489, 1444, 1409, 1314, 1266, 1250, 1209, 1178, 1156, 1122, 1038, 1000, 939, 867, 831, 794, 765, 718; ^{1}H-NMR (DMSO-d_6), δ: 13.70 (s, 1H), 10.54 (s, 1H), 8.31 (d, 1H, *J*=8.2 Hz), 8.06 (d, 1H, *J*=9.2 Hz), 7.93 (d, 1H, *J*=8.2 Hz), 7.68-7.72 (m, 1H), 7.58-7.62 (m, 1H), 7.50 (d, 1H, *J*=8.7 Hz), 7.38-7.45 (m, 2H); ^{13}C-NMR (DMSO-d_6), δ: 170.19, 160.68 (dt, *J*=247.3, 15.3 Hz), 160.15, 158.37 (ddd, *J*=249.8, 16.0, 7.3 Hz), 136.22, 129.51, 127.63, 126.21, 124.54, 123.17, 122.70, 118.39, 110.53 (dt, *J*=17.3, 5.1 Hz), 106.31, 101.23 (t, *J*=27.2 Hz); ^{19}F-NMR (DMSO-d_6), δ: -108.45 (m), -114.75 (m); HR-MS: $[M-H]^+$ calculated 316.0580 m/z, found 316.05896 m/z.

1-hydroxy-*N*-(3,4,5-trifluorophenyl)naphthalene-2-carboxamide (**4l**). Yield 76%; Mp. 242-247°C; HPLC purity 95.24%; IR (cm^{-1}): 3429, 1625, 1598, 1550, 1526, 1504, 1463, 1437, 1408, 1388, 1358, 1325, 1289, 1274, 1252, 1235, 1211, 1181, 1159, 1046, 932, 861, 803, 791, 764, 723, 694; ^{1}H-NMR (DMSO-d_6), δ: 13.49 (s, 1H), 10.62 (s, 1H), 8.29 (d, 1H, *J*=8.2 Hz), 8.01 (d, 1H, *J*=9.2 Hz), 7.90 (d, 1H, *J*=8.2 Hz), 7.65-7.75 (m, 3H), 7.58 (ddd, 1H, *J*=8.2, *J*=6.9, *J*=1.4 Hz), 7.47 (d, 1H, *J*=9.2 Hz); ^{13}C-NMR (DMSO-d_6), δ: 169.65, 159.88, 149.90 (ddd, *J*=244.5, 10.0, 5.3 Hz), 136.14, 134.14 (dt, *J*=11.8, 3.9 Hz), 129.41, 127.55, 126.12, 124.59, 123.30 (dt, *J*=246.3, 15.5 Hz), 123.14, 122.86, 118.14, 107.40, 106.14 (m); ^{19}F-NMR (DMSO-d_6), δ: -134.92 (dd, *J*=22.4, 10.3 Hz), -166.47 (tt, *J*=22.4, 6.6 Hz); HR-MS: $[M-H]^+$ calculated 316.05799 m/z, found 316.05902 m/z.

1-hydroxy-*N*-(2,3,5,6-tetrafluorophenyl)naphthalene-2-carboxamide (**4m)**. Yield 69%; Mp. 160-163°C; HPLC purity 98.03%; IR (cm^{-1}): 3418, 1627, 1598, 1524, 1495, 1470, 1404, 1393, 1248, 1205, 1172, 1141, 1092, 964, 913, 823, 793, 764, 715; ^{1}H-NMR (DMSO-*d*$_6$), δ: 13.42 (br. s, 1H), 10.91 (s, 1H), 8.32 (d, 1H, *J*=8.2 Hz), 8.07 (d, 1H, *J*=8.7 Hz), 7.92-8.03 (m, 2H), 7.68-7.73 (m, 1H), 7.58-7.63 (m, 1H), 7.52 (d, 1H, *J*=9.2 Hz); ^{13}C-NMR (DMSO-*d*$_6$), δ: 169.85, 160.17, 145.51 (ddt, *J*=245.0, 13.0, 3.8 Hz), 142.58 (ddd, *J*=248.0, 14.7, 3.4 Hz), 136.32, 129.66, 127.65, 126.30, 124.50, 123.22, 122.81, 118.56, 116.72 (tt, *J*=14.8, 3.0 Hz), 106.30, 105.54 (t, *J*=23.6 Hz); ^{19}F-NMR (DMSO-*d*$_6$), δ: -139.62 (m), -145.09 (m); HR-MS: $[M-H]^+$ calculated 334.04857 m/z, found 334.04919 m/z.

1-hydroxy-*N*-(2,3,4,5,6-pentafluorophenyl)naphthalene-2-carboxamide (**4n**). Yield 66%; Mp. 230-234°C; HPLC purity 97.96%; IR (cm^{-1}): 3360, 1621, 1598, 1522, 1510, 1502, 1496, 1410, 1389, 1328, 1312, 1274, 1250, 1209, 1132, 1088, 994, 940, 804, 791, 761, 725; ^{1}H-NMR (DMSO-*d*$_6$), δ: 13.38 (br. s, 1H), 10.88 (s, 1H), 8.31 (d, 1H, *J*=8.2 Hz), 8.06 (d, 1H, *J*=8.8 Hz), 7.94 (d, 1H, *J*=8.2 Hz), 7.70-7.73 (m, 1H), 7.60-7.63 (m, 1H), 7.52 (d, 1H, *J*=8.8 Hz); ^{13}C-NMR (DMSO-*d*$_6$), δ: 169.91, 160.22, 143.12 (dm, *J*=248.8 Hz), 139.73 (dm, *J*=251.1 Hz), 137.37 (dm, *J*=247.7 Hz), 136.31, 129.65, 127.62, 126.27, 124.45, 123.19, 122.76, 118.55, 112.02 (t, *J*=15.2 Hz), 106.22; ^{19}F-NMR (DMSO-*d*$_6$), δ: -145.01 (d, *J*=24.5 Hz), -156.10 (t, *J*=22.9 Hz), -162.92 (t, *J*=24.5 Hz); HR-MS: $[M-H]^+$ calculated 352.03915 m/z, found 352.04007 m/z.

N-(3-chloro-2-fluorophenyl)-1-hydroxynaphthalene-2-carboxamide (**4o**). Yield 59%; Mp. 172-174°C; HPLC purity 98.87%; IR (cm^{-1}): 3458, 1629, 1609, 1598, 1576, 1544, 1505, 1452, 1412, 1389, 1333, 1305, 1272, 1243, 1220, 1208, 1150, 1141, 1090, 1064, 1033, 1024, 872, 828, 784, 767, 753, 725, 705, 667; ^{1}H-NMR (DMSO-*d*$_6$), δ: 13.70 (s, 1H), 10.73 (s, 1H), 8.32 (d, *J* = 8.1 Hz, 1H), 8.06 (d, *J* = 8.8 Hz, 1H), 7.93 (d, *J* = 7.8 Hz, 1H), 7.69 (t, *J* = 7.0 Hz, 1H), 7.53 – 7.62 (m, 3H), 7.49 (d, *J* = 8.8 Hz, 1H), 7.31 (t, *J* = 7.6 Hz, 1H); ^{13}C-NMR (DMSO-*d*$_6$), δ: 169.52, 159.78, 152.02 (d, *J* = 249.8 Hz), 136.15, 129.33, 128.21, 127.59, 126.79, 126.10 (d, *J* = 11.8 Hz), 126.09, 125.08 (d, *J* = 4.5 Hz), 124.59, 123.14, 123.08, 120.22 (d, *J* = 16.3 Hz), 118.28, 107.09; HR-MS: for $C_{17}H_{11}ClFNO_2$ $[M+H]^+$ calculated 316.053511 *m/z* found 316.05408 *m/z*.

N-[2-fluoro-3-(trifluoromethyl)phenyl]-1-hydroxynaphthalene-2-carboxamide (**4p**). Yield 45%; Mp. 174-175°C; HPLC purity 97.87%; IR (cm^{-1}): 3462, 1625, 1598, 1576, 1552, 1505, 1489, 1470, 1456, 1412, 1392, 1327, 1273, 1245, 1221, 1191, 1175, 1156, 1125, 1111, 1088, 1069,

1033, 975, 906, 875, 817, 784, 760, 720; ^{1}H-NMR (DMSO-d_6), δ: 13.61 (br. s, 1H), 10.79 (s, 1H), 8.32 (d, J = 8.3 Hz, 1H), 8.07 (d, J = 8.8 Hz, 1H), 7.97 (t, J = 7.6 Hz, 1H), 7.94 (d, J = 8.6 Hz, 1H), 7.74 (t, J = 7.2 Hz, 1H), 7.67-7.72 (m, 1H), 7.58-7.63 (m, 1H), 7.48-7.53 (m, 2H); ^{13}C-NMR (DMSO-d_6), δ: 169.54, 159.76, 153.21 (dq, J = 257.0, 1.8 Hz), 136.18, 132.64, 129.36, 127.59, 126.10, 126.05 (d, J = 11.8 Hz), 124.99 (d, J = 4.5 Hz), 124.62 (q, J = 4.5 Hz), 124.59, 123.15, 123.08, 122.57 (q, J = 272.5 Hz), 118.35, 117.41 (qd, J = 31.8, 10.9 Hz), 107.12; HR-MS: for $C_{18}H_{11}F_4NO_2$ $[M+H]^+$ calculated 350.079868 *m/z* found 350.08038 *m/z*.

N-(4-chloro-2-fluorophenyl)-1-hydroxynaphthalene-2-carboxamide (**4q**). Yield 63%; Mp. 163-165°C; HPLC purity 98.87%; IR (cm^{-1}): 3440, 1643, 1601, 1524, 1504, 1486, 1468, 1409, 1393, 1358, 1336, 1302, 1264, 1247, 1237, 1207, 1185, 1154, 1114, 1088, 1074, 943, 891, 856, 836, 810, 802, 784, 755, 721; ^{1}H-NMR (DMSO-d_6), δ: 13.73 (s, 1H), 10.64 (s, 1H), 8.31 (d, J = 8.3 Hz, 1H), 8.06 (d, J = 8.8 Hz, 1H), 7.92 (d, J = 8.1 Hz, 1H), 7.57 – 7.71 (m, 4H), 7.48 (d, J = 8.6 Hz, 1H), 7.38 (d, J = 8.3 Hz, 1H); ^{13}C-NMR (DMSO-d_6), δ: 169.45, 129.68, 156.11 (d, J = 251.6 Hz), 136.10, 131.07 (d, J = 10.0 Hz), 129.27, 128.95 (d, J = 1.8 Hz), 127.57, 126.04, 124.76 (d, J = 3.6 Hz), 124.59, 123.75 (d, J = 12.7 Hz), 123.11, 123.08, 118.24, 116.66 (d, J = 23.6 Hz), 107.18; HR-MS: for $C_{17}H_{11}ClFNO_2$ $[M+H]^+$ calculated 316.053511 *m/z* found 316.05411 *m/z*.

N-(4-bromo-2-fluorophenyl)-1-hydroxynaphthalene-2-carboxamide (**4r**). Yield 74%; Mp. 172-174°C; HPLC purity 98.80%; IR (cm^{-1}): 3447, 1643, 1630, 1612, 1600, 1575, 1530, 1504, 1485, 1468, 1407, 1394, 1357, 1336, 1307, 1274, 1262, 1247, 1237, 1208, 1184, 1155, 1113, 1088, 1066, 943, 866, 856, 803, 786, 780, 755, 721; ^{1}H-NMR (DMSO-d_6), δ: 13.72 (s, 1H), 10.63 (s, 1H), 8.31 (d, J = 8.1 Hz, 1H), 8.06 (d, J = 8.8 Hz, 1H), 7.92 (d, J = 8.1 Hz, 1H), 7.66-7.74 (m, 2H), 7.57-7.62 (m, 2H), 7.47-7.52 (m, 2H); ^{13}C-NMR (DMSO-d_6), δ: 169.37, 159.66, 156.12 (d, J = 252.5 Hz), 136.11, 129.27, 129.23 (d, J = 1.8 Hz), 127.68 (d, J = 3.6 Hz), 127.57, 126.05, 124.59, 124.17 (d, J = 12.7 Hz), 123.11, 123.10, 119.40 (d, J = 22.7 Hz), 118.77 (d, J = 9.1 Hz), 118.24, 107.22; HR-MS: for $C_{17}H_{11}BrFNO_2$ $[M+H]^+$ calculated 360.002988 *m/z* found 360.00363 *m/z*.

N-(5-chloro-2-fluorophenyl)-1-hydroxynaphthalene-2-carboxamide (**4s**). Yield 67%; Mp. 169-171°C; HPLC purity 98.06%; IR (cm^{-1}): 3433, 1644, 1613, 1600, 1569, 1531, 1473, 1406, 1389, 1359, 1325, 1296, 1268, 1245, 1208, 1179, 1144, 1102, 1082, 1021, 940, 911, 871, 846, 802, 784, 770, 752, 720; ^{1}H-NMR (DMSO-d_6), δ: 13.58 (br. s, 1H), 10.69 (s, 1H), 8.32 (d, J = 8.3 Hz, 1H), 8.05 (d, J = 8.8 Hz, 1H), 7.93 (d, J = 8.1

Hz, 1H), 7.78 – 7.81 (m, 1H), 7.66-7.71 (m, 1H), 7.57-7.62 (m, 1H), 7.49 (t, *J* = 8.8 Hz, 1H), 7.40-7.44 (m, 2H); ^{13}C-NMR (DMSO-*d*$_6$), δ: 169.19, 159.41, 154.77 (d, *J* = 247.0 Hz), 136.14, 129.27, 127.91 (d, *J* = 2.7 Hz), 127.59, 127.27 (d, *J* = 8.2 Hz), 127.00 (d, *J* = 1.8 Hz), 126.07, 126.05 (d, *J* = 13.6 Hz), 124.61, 123.23, 123.13, 118.37, 117.56 (d, *J* = 21.8 Hz), 107.51; HR-MS: for $C_{17}H_{11}ClFNO_2$ $[M+H]^+$ calculated 316.053511 *m/z* found 316.05380 *m/z.*

N-(5-bromo-2-fluorophenyl)-1-hydroxynaphthalene-2-carboxamide (**4t**). Yield 60%; Mp. 174-177°C; HPLC purity 99.80%; IR (cm^{-1}): 3443, 1634, 1612, 1600, 1530, 1503, 1479, 1410, 1389, 1361, 1326, 1303, 1271, 1242, 1209, 1181, 1166, 1151, 1102, 1086, 865, 832, 799, 787, 771, 758, 720; ^{1}H-NMR (DMSO-*d*$_6$), δ: 13.59 (s, 1H), 10.69 (s, 1H), 8.32 (d, *J* = 8.3 Hz, 1H), 8.05 (d, *J* = 8.8 Hz, 1H), 7.90-7.94 (m, 2H), 7.69 (t, *J* = 7.5 Hz, 1H), 7.57-7.62 (m, 1H), 7.52-7.56 (m, 1H), 7.49 (d, *J* = 8.8 Hz, 1H), 7.37 (t, *J* = 9.5 Hz, 1H); ^{13}C-NMR (DMSO-*d*$_6$), δ: 169.21, 159.44, 155.28 (d, *J* = 248.0 Hz), 136.13, 130.24 (d, *J* = 8.2 Hz), 129.89 (d, *J* = 1.8 Hz), 129.28, 127.59, 126.36 (d, *J* = 13.6 Hz), 126.07, 124.61, 123.21, 123.13, 118.35, 118.01 (d, *J* = 21.8 Hz), 115.58 (d, *J* = 2.7 Hz), 107.48; HR-MS: for $C_{17}H_{11}BrFNO_2$ $[M+H]^+$ calculated 360.002988 *m/z* found 360.00348 *m/z.*

N-[2-fluoro-5-(trifluoromethyl)phenyl]-1-hydroxynaphthalene-2-carboxamide (**4u**). Yield 65%; Mp. 190-192°C; HPLC purity 98.15%; IR (cm^{-1}): 3440, 1645, 1617, 1595, 1552, 1543, 1485, 1436, 1412, 1394, 1334, 1307, 1276, 1261, 1240, 1208, 1185, 1172, 1165, 1153, 1104, 1066, 944, 920, 895, 872, 852, 824, 803, 787, 763, 720; ^{1}H-NMR (DMSO-*d*$_6$), δ: 13.52 (s, 1H), 10.81 (s, 1H), 8.33 (d, *J* = 8.3 Hz, 1H), 8.12 (dd, *J* = 6.8, 1.5 Hz, 1H), 8.06 (d, *J* = 8.8 Hz, 1H), 7.93 (d, *J* = 8.1 Hz, 1H), 7.73-7.78 (m, 1H), 7.69 (ddd, *J* = 8.0, 7.2, 1.0 Hz, 1H), 7.58-7.66 (m, 2H), 7.51 (d, *J* = 8.8 Hz, 1H); ^{13}C-NMR (DMSO-*d*$_6$), δ: 169.29, 159.41, 157.97 (d, *J* = 254.3 Hz), 136.18, 129.31, 127.61, 126.09, 125.76 (d, *J* = 13.0 Hz), 125.43 (qd, *J* = 32.7, 3.6 Hz), 124.89 (dq, *J* = 9.1, 4.5 Hz), 124.63, 124.61 (quin, *J* = 3.6 Hz), 123.64 (q, *J* = 272.5 Hz), 123.27, 123.16, 118.44, 117.29 (d, *J* = 20.9 Hz), 107.55; HR-MS: for $C_{18}H_{11}F_4NO_2$ $[M+H]^+$ calculated 350.079868 *m/z* found 350.08011 *m/z.*

N-(4-bromo-3-fluorophenyl)-1-hydroxynaphthalene-2-carboxamide (**4v**). Yield 58%; Mp. 199-202°C; HPLC purity 99.68%; IR (cm^{-1}): 3451, 1628, 1598, 1575, 1524, 1502, 1487, 1464, 1408, 1387, 1357, 1317, 1270, 1243, 1205, 1184, 1146, 1086, 1038, 1024, 973, 939, 863, 822, 811, 798, 783, 749, 716; ^{1}H-NMR (DMSO-*d*$_6$), δ: 13.62 (s, 1H), 10.66 (s, 1H), 8.32 (d, *J* = 8.3 Hz, 1H), 8.08 (d, *J* = 8.8 Hz, 1H), 7.92 (d, *J* = 8.3 Hz,

1H), 7.88 (dd, *J* = 11.2, 2.1 Hz, 1H), 7.74 (t, *J* = 8.3 Hz, 1H), 7.69 (t, *J* = 7.5 Hz, 1H), 7.56-7.62 (m, 2H), 7.49 (d, *J* = 8.8 Hz, 1H); ^{13}C-NMR (DMSO-d_6), δ: 169.60, 159.89, 157.88 (d, *J* = 242.5 Hz), 139.00 (d, *J* = 10.0 Hz), 136.08, 133.23 (d, *J* = 1.8 Hz), 129.32, 127.51, 126.04, 124.58, 123.10, 122.97, 118.96 (d, *J* = 2.7 Hz), 118.01, 109.72 (d, *J* = 27.2 Hz), 107.49, 102.48 (d, *J* = 20.9 Hz); HR-MS: for $C_{17}H_{11}BrFNO_2$ $[M+H]^+$ calculated 360.002988 *m/z* found 360.00333 *m/z.*

N-[3-fluoro-4-(trifluoromethyl)phenyl]-1-hydroxynaphthalene-2-carboxamide (**4w**). Yield 40%; Mp. 185-187°C; HPLC purity 95.53%; IR (cm^{-1}): 3425, 1635, 1618, 1601, 1574, 1532, 1504, 1471, 1428, 1411, 1393, 1316, 1269, 1246, 1209, 1188, 1158, 1134, 1109, 1046, 1022, 975, 951, 940, 872, 815, 803, 788, 762, 754, 729; ^{1}H-NMR (DMSO-d_6), δ: 13.41 (br. s, 1H), 10.83 (s, 1H), 8.32 (d, *J* = 8.3 Hz, 1H), 8.09 (d, *J* = 8.8 Hz, 1H), 7.99 (d, *J* = 13.4 Hz, 1H), 7.93 (d, *J* = 8.1 Hz, 1H), 7.78-7.85 (m, 2H), 7.69 (t, *J* = 7.5 Hz, 1H), 7.57-7.62 (m, 1H), 7.50 (d, *J* = 8.8 Hz, 1H); ^{13}C-NMR (DMSO-d_6), δ: 169.91, 159.95, 158.88 (dq, *J* = 250.7, 1.8 Hz), 143.78 (d, *J* = 10.9 Hz), 136.18, 129.42, 127.61 (qd, *J* = 4.5, 2.7 Hz), 127.52, 126.08, 124.56, 123.15, 123.05, 122.74 (q, *J* = 271.6 Hz), 118.11, 116.87 (d, *J* = 2.7 Hz), 111.69 (qd, *J* = 32.7, 12.7 Hz), 108.99 (d, *J* = 25.4 Hz), 107.58; HR-MS: for $C_{18}H_{11}F_4NO_2$ $[M+H]^+$ calculated 350.079868 *m/z* found 350.08026 *m/z.*

N-[3-fluoro-5-(trifluoromethyl)phenyl]-1-hydroxynaphthalene-2-carboxamide (**4x**). Yield 48%; Mp. 165-167°C; HPLC purity 99.32%; IR (cm^{-1}): 3443, 1623, 1596, 1576, 1544, 1505, 1482, 1467, 1433, 1413, 1392, 1354, 1330, 1317, 1277, 1234, 1208, 1170, 1152, 1119, 1085, 994, 878, 856, 821, 787, 761, 728, 712, 688; ^{1}H-NMR (DMSO-d_6), δ: 13.43 (s, 1H), 10.81 (s, 1H), 8.32 (d, *J* = 8.3 Hz, 1H), 8.07 (d, *J* = 9.1 Hz, 1H), 8.01-8.06 (m, 2H), 7.93 (d, *J* = 8.1 Hz, 1H), 7.70 (t, *J* = 7.5 Hz, 1H), 7.58-7.63 (m, 1H), 7.48-7.53 (m, 2H); ^{13}C-NMR (DMSO-d_6), δ: 169.81, 161.90 (d, *J* = 244.3 Hz), 159.94, 140.62 (d, *J* = 11.8 Hz), 136.15, 130.89 (qd, *J* = 32.7, 9.1 Hz), 129.40, 127.52, 126.07, 124.57, 123.29 (qd, *J* = 272.5, 3.6 Hz), 123.14, 122.92, 118.11, 113.89 (qd, *J* = 3.6, 3.6 Hz), 111.94 (d, *J* = 26.3 Hz), 108.16 (dq, *J* = 25.4, 3.6 Hz), 107.43; HR-MS: for $C_{18}H_{11}F_4NO_2$ $[M-H]^+$ calculated 348.064218 *m/z* found 348.06512 *m/z.*

N-(4-bromo-2,3,5,6-tetrafluorophenyl)-1-hydroxynaphthalene-2-carboxamide (**4y**). Yield 24%; Mp. 220-223°C; HPLC purity 100%; IR (cm^{-1}): 3284, 1615, 1597, 1520, 1495, 1475, 1407, 1390, 1320, 1250, 1207, 1170, 1149, 1095, 1065, 977, 939, 878, 851, 815, 790, 765, 726, 714, 658; ^{1}H-NMR (DMSO-d_6), δ: 13.35 (br. s, 1H), 10.96 (s, 1H), 8.32 (d, *J* = 8.3 Hz, 1H), 8.07 (d, *J* = 8.8 Hz, 1H), 7.94 (d, *J* = 8.1 Hz, 1H), 7.71 (t, *J* =

7.5 Hz, 1H), 7.59-7.64 (m, 1H), 7.52 (d, *J* = 9.1 Hz, 1H); ^{13}C-NMR (DMSO-*d*$_6$), δ: 169.71, 160.14, 144.52 (dm, *J* = 244.3 Hz), 142.90 (ddt, *J* = 250.7, 15.4, 3.6 Hz), 136.32, 129.66, 127.62, 126.27, 124.45, 123.20, 122.82, 118.55, 115.97 (tt, *J* = 15.4, 2.3 Hz), 106.27, 98.32 (t, *J* = 23.3 Hz); HR-MS: for $C_{17}H_8BrF_4NO_2$ [M-H]$^+$ calculated 411.959073 *m/z* found 411.96066 *m/z.*

N-[2,5-difluoro-4-(trifluoromethyl)phenyl]-1-hydroxynaphthalene-2-carboxamide (**4z**). Yield 38%; Mp. 229-231°C; HPLC purity 100%; IR (cm^{-1}): 3443, 1651, 1627, 1615, 1532, 1495, 1442, 1413, 1394, 1365, 1326, 1287, 1271, 1248, 1210, 1176, 1151, 1125, 1023, 885, 861, 840, 801, 786, 759, 734, 723, 715; ^{1}H-NMR (DMSO-*d*$_6$), δ: 13.14 (br. s, 1H), 11.05 (s, 1H), 8.35 (d, *J* = 8.3 Hz, 1H), 8.10 (dd, *J* = 11.8, 6.0 Hz, 1H), 8.06 (d, *J* = 9.1 Hz, 1H), 7.90-7.96 (m, 2H), 7.70 (t, *J* = 7.3 Hz, 1H), 7.58-7.64 (m, 1H), 7.52 (d, *J* = 8.8 Hz, 1H); ^{13}C-NMR (DMSO-*d*$_6$), δ: 168.31, 158.45, 154.68 (dq, *J* = 249.8, 1.8 Hz), 150.31 (dd, *J* = 244.3, 1.8 Hz), 136.29, 130.97 (t, *J* = 12.7 Hz), 129.31, 127.67, 126.12, 124.69, 123.78, 123.21, 121.92 (qd, *J* = 272.5, 1.8 Hz), 118.87, 114.63-114.76 (m), 114.42-114.57 (m), 113.82 (dd, *J* = 27.2, 1.8 Hz), 108.72; HR-MS: for $C_{18}H_{10}F_5NO_2$ [M-H]$^+$ calculated 366.054796 *m/z* found 366.05630 *m/z.*

N-(2-chloro-4-fluorophenyl)-1-hydroxynaphthalene-2-carboxamide (**5m**). Yield 84%; Mp. 162-166°C; HPLC purity 100%; IR (cm^{-1}): 3429, 1626, 1596, 1576, 1537, 1504, 1480, 1458, 1406, 1387, 1326, 1304, 1243, 1209, 1188, 1156, 1145, 1089, 1033, 1021, 902, 860, 829, 795, 760, 715; ^{1}H-NMR (DMSO-*d*$_6$), δ: 13.82 (s, 1H), 10.64 (s, 1H), 8.31 (d, *J* = 8.3 Hz, 1H), 8.08 (d, *J* = 8.8 Hz, 1H), 7.92 (d, *J* = 8.1 Hz, 1H), 7.66-7.71 (m, 2H), 7.57-7.65 (m, 2H), 7.48 (d, *J* = 8.8 Hz, 1H), 7.33 (t, *J* = 8.3 Hz, 1H); ^{13}C-NMR (DMSO-*d*$_6$), δ: 169.65, 160.24 (d, *J* = 247.0 Hz), 159.67, 136.09, 131.37 (d, *J* = 10.9 Hz), 130.73 (d, *J* = 2.7 Hz), 130.51 (d, *J* = 9.1 Hz), 129.21, 127.57, 126.02, 124.64, 123.09, 123.04, 118.22, 116.89 (d, *J* = 26.3 Hz), 114.84 (d, *J* = 22.7 Hz), 107.23; HR-MS: for $C_{17}H_{11}ClFNO_2$ [M+H]$^+$ calculated 316.053511 *m/z* found 316.05414 *m/z.*

N-[2-chloro-4-(trifluoromethyl)phenyl]-1-hydroxynaphthalene-2-carboxamide (**5o**). Yield 32%; Mp. 158-160°C; HPLC purity 100%; IR (cm^{-1}): 3418, 1634, 1598, 1586, 1574, 1533, 1505, 1486, 1393, 1318, 1294, 1265, 1241, 1205, 1146, 1113, 1076, 1055, 1033, 942, 887, 829, 758, 723, 704; ^{1}H-NMR (DMSO-*d*$_6$), δ: 13.33 (br. s, 1H), 11.02 (s, 1H), 8.35 (d, *J* = 8.3 Hz, 1H), 8.13 (d, *J* = 8.3 Hz, 1H), 8.09 (d, *J* = 8.8 Hz, 1H), 8.05 (s, 1H), 7.94 (d, *J* = 8.1 Hz, 1H), 7.83 (d, *J* = 8.3 Hz, 1H), 7.67-7.72 (m, 1H), 7.58-7.64 (m, 1H), 7.53 (d, *J* = 8.8 Hz, 1H); ^{13}C-NMR (DMSO-*d*$_6$), δ: 168.36, 158.43, 138.38, 136.22, 129.21, 128.79, 127.66, 127.58, 127.34 (q, *J* = 32.7 Hz), 126.68 (q, *J* = 3.8 Hz), 126.09, 124.82 (q, *J* = 3.8 Hz),

124.77, 123.73, 123.35 (q, J = 272.5 Hz), 123.18, 118.82, 108.75; HR-MS: for $C_{18}H_{11}ClF_3NO_2$ $[M-H]^+$ calculated 364.034667 *m/z* found 364.03598 *m/z.*

N-(2-chloro-5-methoxyphenyl)-1-hydroxynaphthalene-2-carboxamide (**5p**). Yield 71%; Mp. 159-161°C; HPLC purity 99.01%; IR (cm^{-1}): 3422, 1634, 1597, 1587, 1575, 1532, 1504, 1477, 1458, 1439, 1408, 1388, 1359, 1330, 1294, 1270, 1243, 1203, 1177, 1153, 1123, 1088, 1052, 1021, 965, 941, 853, 834, 796, 787, 758, 746, 725, 716; ^{1}H-NMR (DMSO-d_6), δ: 13.71 (br. s, 1H), 10.68 (s, 1H), 8.32 (d, J = 8.3 Hz, 1H), 8.09 (d, J = 8.6 Hz, 1H), 7.93 (d, J = 8.1 Hz, 1H), 7.68 (t, J = 7.2 Hz, 1H), 7.57-7.62 (m, 1H), 7.49 (d, J = 8.6 Hz, 2H), 7.35 (s, 1H), 6.95 (d, J = 8.8 Hz, 1H), 3.80 (s, 3H); ^{13}C-NMR (DMSO-d_6), δ: 168.98, 159.12, 158.37, 136.09, 134.93, 129.96, 129.12, 127.60, 126.00, 124.72, 123.37, 123.12, 120.66, 118.38, 113.70, 113.64, 108.01, 55.66; HR-MS: for $C_{18}H_{14}ClNO_3$ $[M+H]^+$ calculated 328.073497 *m/z* found 328.07404 *m/z.*

N-[2-chloro-5-(trifluoromethyl)phenyl]-1-hydroxynaphthalene-2-carboxamide (**5r**). Yield 67%; Mp. 182-183°C; HPLC purity 98.69%; IR (cm^{-1}): 3423, 1644, 1632, 1595, 1540, 1504, 1429, 1406, 1390, 1328, 1291, 1263, 1246, 1233, 1173, 1149, 1113, 1077, 895, 815, 784, 760, 720; ^{1}H-NMR (DMSO-d_6), δ: 13.59 (br. s, 1H), 10.96 (s, 1H), 8.34 (d, 1H, J=7.7 Hz), 8.25 (s, 1H), 8.09 (d, 1H, J=8.8 Hz), 7.93 (d, 1H, J=8.1 Hz), 7.87 (d, 1H, J=8.4 Hz), 7.70 (d, 1H, J=7.2 Hz), 7.68 (ddd, 1H, J=8.2, J=6.9, J=1.3 Hz), 7.57 (ddd, 1H, J=8.3, J=7.0, J=1.2 Hz), 7.51 (d, 1H, J=7.8 Hz); ^{13}C-NMR (DMSO-d_6), δ: 168.78, 158.76, 136.18, 135.38, 133.18, 130.86, 129.20, 128.35 (q, J=32.8 Hz), 127.64, 126.08, 124.73, 124.36 (q, J=3.8 Hz), 124.11 (q, J=3.8 Hz), 123.55 (q, J=272.4 Hz), 123.54, 123.15, 118.66, 108.33; HR-MS: $[M-H]^+$ calculated 364.03467 m/z, found 364.03635 m/z.

N-(3-chloro-4-fluorophenyl)-1-hydroxynaphthalene-2-carboxamide (**5s**). Yield 71%; Mp. 202-204°C; HPLC purity 98.98%; IR (cm^{-1}): 3429, 1626, 1601, 1539, 1498, 1467, 1401, 1388, 1360, 1313, 1270, 1243, 1205, 1150, 1052, 879, 819, 800, 787, 758, 721, 691; ^{1}H-NMR (DMSO-d_6), δ: 13.72 (s, 1H), 10.59 (s, 1H), 8.31 (d, J = 8.3 Hz, 1H), 8.07 (d, J = 8.8 Hz, 1H), 8.02 (d, J = 6.1 Hz, 1H), 7.92 (d, J = 7.8 Hz, 1H), 7.65-7.75 (m, 2H), 7.56-7.61 (m, 1H), 7.44-7.50 (m, 2H); ^{13}C-NMR (DMSO-d_6), δ: 169.46, 159.91, 154.03 (d, J = 244.4 Hz), 136.02, 134.87 (d, J = 2.9 Hz), 129.21, 127.46, 125.96, 124.58, 123.57, 123.07, 122.88, 122.35 (d, J = 6.6 Hz), 119.07 (d, J = 18.3 Hz), 117.92, 116.78 (d, J = 22.0 Hz), 107.31; HR-MS: for $C_{17}H_{11}ClFNO_2$ $[M+H]^+$ calculated 316.053511 *m/z* found 316.05411 *m/z.*

N-[2-chloro-3,5-bis(trifluoromethyl)phenyl]-1-hydroxynaphthalene-2-carboxamide (**5u**). Yield 37%; Mp. 192-193°C; HPLC purity 100%; IR (cm^{-1}): 3422, 3107, 1652, 1598, 1539, 1505, 1446, 1409, 1394, 1370, 1286, 1259, 1243, 1194, 1167, 1125, 1108, 1087, 962, 926, 912, 889, 852, 825, 789, 763, 720, 704; ^{1}H-NMR (DMSO-d_6), δ: 13.26 (br. s, 1H), 11.18 (s, 1H), 8.65 (s, 1H), 8.35 (d, J = 8.1 Hz, 1H), 8.11 (s, 1H), 8.08 (d, J = 9.1 Hz, 1H), 7.94 (d, J = 7.8 Hz, 1H), 7.70 (t, J = 7.1 Hz, 1H), 7.58-7.63 (m, 1H), 7.53 (d, J = 8.3 Hz, 1H); ^{13}C-NMR (DMSO-d_6), δ: 169.22, 159.18, 138.49, 136.76, 131.52, 129.80, 129.36 (q, J = 31.8 Hz), 128.92 (q, J = 33.7 Hz), 128.78, 128.16, 126.62, 125.20, 124.10, 123.69, 123.32 (q, J = 273.4 Hz), 122.54 (q, J = 273.4 Hz), 122.20-122.36 (m), 119.31, 108.87; HR-MS: for $C_{19}H_{10}ClF_6NO_2$ [M-H]$^+$ calculated 432.022052 *m/z* found 432.02371 *m/z.*

N-[2,6-dichloro-4-(trifluoromethyl)phenyl]-1-hydroxynaphthalene-2-carboxamide (**5v**). Yield 16%; Mp. 191-194°C; HPLC purity 98.45%; IR (cm^{-1}): 3263, 3082, 3053, 1620, 1596, 1567, 1524, 1501, 1477, 1388, 1312, 1269, 1246, 1208, 1176, 1130, 1109, 1090, 1024, 948, 896, 876, 843, 805, 789, 758, 707, 680; ^{1}H-NMR (DMSO-d_6), δ: 13.61 (s, 1H), 10.97 (s, 1H), 8.31 (d, J = 8.3 Hz, 1H), 8.15 (s, 2H), 8.10 (d, J = 9.1 Hz, 1H), 7.95 (d, J = 8.1 Hz, 1H), 7.71 (t, J = 7.3 Hz, 1H), 7.59-7.64 (m, 1H), 7.53 (d, J = 8.8 Hz, 1H); ^{13}C-NMR (DMSO-d_6), δ: 169.81, 160.25, 136.40, 136.25, 135.25, 130.16 (q, J = 33.6 Hz), 129.52, 127.62, 126.21, 125.82 (q, J = 3.6 Hz), 124.53, 123.11, 122.72, 122.50 (q, J = 273.4 Hz), 118.42, 106.26; HR-MS: for $C_{18}H_{10}Cl_2F_3NO_2$ [M-H]$^+$ calculated 397.995695 *m/z* found 397.99734 *m/z.*

N-[2-bromo-4-(trifluoromethyl)phenyl]-1-hydroxynaphthalene-2-carboxamide (**6i**). Yield 37%; Mp. 168-170°C; HPLC purity 99.32%; IR (cm^{-1}): 3404, 1634, 1612, 1585, 1534, 1506, 1486, 1469, 1403, 1391, 1307, 1272, 1242, 1178, 1170, 1152, 1131, 1110, 1077, 1043, 1022, 963, 941, 890, 831, 820, 800, 788, 761, 720, 687; ^{1}H-NMR (DMSO-d_6), δ: 13.43 (s, 1H), 10.93 (s, 1H), 8.34 (d, J = 8.1 Hz, 1H), 8.17 (s, 1H), 8.09 (d, J = 8.8 Hz, 1H), 8.05 (d, J = 8.3 Hz, 1H), 7.94 (d, J = 8.3 Hz, 1H), 7.87 (d, J = 8.3 Hz, 1H), 7.67-7.72 (m, 1H), 7.58-7.63 (m, 1H), 7.52 (d, J = 8.8 Hz, 1H); ^{13}C-NMR (DMSO-d_6), δ: 168.61, 158.76, 139.85 (q, J = 1.8 Hz), 136.21, 129.72 (q, J = 3.6 Hz), 129.23, 128.26, 127.86 (q, J = 33.6 Hz), 127.65, 126.09, 125.37 (q, J = 3.6 Hz), 124.74, 123.57, 123.20 (q, J = 272.5 Hz), 123.17, 119.83, 118.71, 108.41; HR-MS: for $C_{18}H_{11}BrF_3NO_2$ [M+H]$^+$ calculated 409.999795 *m/z* found 410.00098 *m/z.*

N-(2-bromo-5-fluorophenyl)-1-hydroxynaphthalene-2-carboxamide (**6j**). Yield 61%; Mp. 187-190°C; HPLC purity 98.12%; IR (cm^{-1}): 3418, 1635, 1596, 1530, 1502, 1471, 1425, 1411, 1389, 1358, 1326, 1293, 1271, 1246, 1208, 1174, 1147, 1021, 980, 868, 839, 799, 789, 762, 751, 724, 717; ^{1}H-NMR (DMSO-d_6), δ: 13.53 (br. s, 1H), 10.78 (s, 1H), 8.34 (d, *J* = 8.3 Hz, 1H), 8.08 (d, *J* = 8.8 Hz, 1H), 7.93 (d, *J* = 8.1 Hz, 1H), 7.81 (dd, *J* = 8.8, 5.8 Hz, 1H), 7.74 (dd, *J* = 10.0, 2.9 Hz, 1H), 7.69 (t, *J* = 7.5 Hz, 1H), 7.58-7.62 (m, 1H), 7.51 (d, *J* = 8.8 Hz, 1H), 7.19 (td, *J* = 8.5, 2.8 Hz, 1H); ^{13}C-NMR (DMSO-d_6), δ: 168.74, 161.17 (d, *J* = 244.3 Hz), 158.89, 137.25 (d, *J* = 11.0 Hz), 136.14, 133.86 (d, *J* = 10.0 Hz), 129.18, 127.62, 126.05, 124.71, 123.43, 123.13, 118.55, 115.36 (d, *J* = 22.9 Hz), 115.17 (d, *J* = 20.9 Hz), 114.58 (d, *J* = 3.6 Hz), 108.18; HR-MS: for $C_{17}H_{11}BrFNO_2$ $[M+H]^+$ calculated 360.002988 *m/z* found 360.00366 *m/z.*

N-[2-bromo-5-(trifluoromethyl)phenyl]-1-hydroxynaphthalene-2-carboxamide (**6k**). Yield 41%; Mp. 190-193°C; HPLC purity 100%; IR (cm^{-1}): 3400, 1642, 1631, 1585, 1538, 1503, 1468, 1424, 1407, 1389, 1327, 1290, 1262, 1246, 1233, 1204, 1173, 1152, 1112, 1076, 1027, 960, 941, 920, 894, 815, 780, 759, 720, 680; ^{1}H-NMR (DMSO-d_6), δ: 13.50 (br. s, 1H), 10.89 (s, 1H), 8.33 (d, *J* = 8.3 Hz, 1H), 8.17 (s, 1H), 8.08 (d, *J* = 8.8 Hz, 1H), 8.03 (d, *J* = 8.3 Hz, 1H), 7.94 (d, *J* = 8.1 Hz, 1H), 7.69 (t, *J* = 7.5 Hz, 1H), 7.58-7.66 (m, 2H), 7.52 (d, *J* = 8.8 Hz, 1H); ^{13}C-NMR (DMSO-d_6), δ: 169.00, 129.04, 136.89, 136.17, 134.09, 129.23, 128.95 (q, *J* = 31.8 Hz), 127.62, 126.07, 124.93 (q, *J* = 3.6 Hz), 124.79, 124.70, 124.56 (q, *J* = 3.6 Hz), 123.45 (q, *J* = 272.5 Hz), 123.40, 123.15, 118.55, 108.03; HR-MS: for $C_{18}H_{11}BrF_3NO_2$ $[M+H]^+$ calculated 409.999795 *m/z* found 410.00098 *m/z.*

N-[2,6-dibromo-4-(trifluoromethyl)phenyl]-1-hydroxynaphthalene-2-carboxamide (**6l**). Yield 21%; Mp. 183-186°C; HPLC purity 98.20%; IR (cm^{-1}): 3274, 3075, 1618, 1595, 1557, 1514, 1500, 1471, 1392, 1374, 1307, 1270, 1257, 1244, 1209, 1200, 1176, 1162, 1124, 1098, 1023, 942, 879, 792, 761, 744, 705, 671; ^{1}H-NMR (DMSO-d_6), δ: 13.68 (s, 1H), 11.00 (s, 1H), 8.31 (d, *J* = 8.3 Hz, 1H), 8.27 (s, 2H), 8.11 (d, *J* = 8.88 Hz, 1H), 7.94 (d, *J* = 8.3 Hz, 1H), 7.71 (t, *J* = 7.5 Hz, 1H), 7.58-7.63 (m, 1H), 7.52 (d, *J* = 8.8 Hz, 1H); ^{13}C-NMR (DMSO-d_6), δ: 169.65, 160.27, 139.32, 136.23, 130.77 (q, *J* = 33.6 Hz), 129.49, 129.29 (q, *J* = 3.6 Hz), 127.62, 126.20, 125.49, 124.53, 123.09, 122.71, 122.23 (q, *J* = 273.4 Hz), 118.37, 106.34; HR-MS: for $C_{18}H_{10}Br_2F_3NO_2$ $[M-H]^+$ calculated 485.89465 *m/z* found 485.89651 *m/z.*

N-(2,6-dibromo-3-chloro-4-fluorophenyl)-1-hydroxynaphthalene-2-carboxamide (**6m**). Yield 39%; Mp. 244-247°C; HPLC purity 99.23%; IR (cm^{-1}): 3306, 3078, 1613, 1596, 1575, 1519, 1498, 1467, 1441,1404, 1385, 1361, 1321, 1297, 1270, 1249, 1208,1152, 1113, 1090, 1022, 978, 941, 856, 792, 763, 717, 700; ^{1}H-NMR (DMSO-d_6), δ: 13.72 (s, 1H), 10.93 (s, 1H), 8.30 (d, J = 8.1 Hz, 1H), 8.16 (d, J = 8.6 Hz, 1H), 8.10 (d, J = 8.8 Hz, 1H), 7.94 (d, J = 8.1 Hz, 1H), 7.70 (t, J = 7.5 Hz, 1H), 7.58-7.63 (m, 1H), 7.52 (d, J = 8.8 Hz, 1H); ^{13}C-NMR (DMSO-d_6), δ: 169.77, 160.24, 156.56 (d, J = 254.3 Hz), 136.20, 133.35 (d, J = 4.5 Hz), 129.45, 127.60, 126.61, 126.17, 124.53, 123.15 (d, J = 10.0 Hz), 123.08, 122.66, 121.52 (d, J = 20.0 Hz), 119.98 (d, J = 25.4 Hz), 118.31, 106.32; HR-MS: for $C_{17}H_9Br_2ClFNO_2$ $[M+H]^+$ calculated 471.874521 *m/z* found 471.87589 *m/z*.

N-[2,4-bis(trifluoromethyl)phenyl]-1-hydroxynaphthalene-2-carboxamide (**7d**). Yield 42%; Mp. 166-169°C; HPLC purity 94.76%; IR (cm^{-1}): 3480, 1646, 1594, 1539, 1506, 1469, 1413, 1389, 1331, 1279, 1245, 1164, 1102, 1055, 1024, 911, 845, 792, 761, 742, 674; ^{1}H-NMR (DMSO-d_6), δ: 13.42 (br. s, 1H), 10.99 (s, 1H), 8.33 (d, J = 8.3 Hz, 1H), 8.20 (d, J = 8.6 Hz, 1H), 8.07 (s, 1H), 8.09 (d, J = 9.1 Hz, 1H), 8.06 (d, J = 9.1 Hz, 1H), 7.94 (d, J = 8.1 Hz, 1H), 7.70 (t, J = 7.5 Hz, 1H), 7.58-7.63 (m, 1H), 7.52 (d, J = 8.8 Hz, 1H); ^{13}C-NMR (DMSO-d_6), δ: 169.60, 158.92, 138.92 (q, J = 1.5 Hz), 136.21, 131.25, 130.37-130.47 (m), 129.25, 127.68 (q, J = 32.9 Hz), 127.62, 126.08, 126.06 (q, J = 30.8 Hz), 124.66, 123.82-124.05 (m), 123.43, 123.29 (q, J = 272.2 Hz), 123.15, 122.77 (q, J = 273.7 Hz), 118.65, 108.00; HR-MS: for $C_{19}H_{11}F_6NO_2$ $[M+H]^+$ calculated 400.076674 *m/z*, found 400.07739 *m/z*.

N-[2,5-bis(trifluoromethyl)phenyl]-1-hydroxynaphthalene-2-carboxamide (**7e**). Yield 41%; Mp. 139-142°C; HPLC purity 99.45%; IR (cm^{-1}): 3480, 1638, 1592, 1555, 1507, 1434, 1410, 1388, 1332, 1315, 1279, 1264, 1255, 1233, 1185, 1126, 1103, 1087, 1035, 960, 899, 841, 783, 760, 747, 721, 669; ^{1}H-NMR (DMSO-d_6), δ: 13.49 (br. s, 1H), 10.91 (s, 1H), 8.32 (d, J = 8.3 Hz, 1H), 8.25 (s, 1H), 8.11 (d, J = 8.3 Hz, 1H), 8.05 (d, J = 8.8 Hz, 1H), 7.97 (d, J = 8.3 Hz, 1H), 8.94 (d, J = 8.1 Hz, 1H), 7.69 (t, J = 7.5 Hz, 1H), 7.58-7.63 (m, 1H), 7.51 (d, J = 8.8 Hz, 1H); ^{13}C-NMR (DMSO-d_6), δ: 170.05, 159.19, 136.16, 136.08 (q, J = 1.5 Hz), 133.40 (qq, J = 39.5, 1.5 Hz), 129.03-129.67 (m), 129.25, 128.26 (q, J = 4.8 Hz), 127.59, 127.48 (q, J = 3.6 Hz), 126.05, 124.62, 124.48 (q, J = 3.6 Hz), 123.28, 123.12, 123.09 (q, J = 273.7 Hz), 122.80 (q, J = 273.7 Hz), 118.48, 107.48; HR-MS: for $C_{19}H_{11}F_6NO_2$ $[M+H]^+$ calculated 400.076674 *m/z*, found 400.07721 *m/z*.

N-[3,5-bis(trifluoromethyl)phenyl]-1-hydroxynaphthalene-2-carboxamide (**7f**). Yield 84%; Mp. 183-186°C; IR (cm^{-1}): 3447, 1636, 1595, 1542, 1505, 1467, 1441, 1379, 1321, 1278, 1246, 1232, 1170, 1118, 1087, 886, 790, 764, 717, 699, 681; ^{1}H-NMR (DMSO-d_6), δ: 13.36 (br. s, 1H), 10.89 (s, 1H), 8.50 (s, 2H), 8.31 (d, 1H, *J*=8.2 Hz), 8.07 (d, 1H, *J*=8.9 Hz), 7.91 (d, 1H, *J*=8.2 Hz), 7.88 (s, 1H), 7.68 (ddd, 1H, *J*=8.2, *J*=6.9, *J*=1.3 Hz), 7.59 (ddd, 1H, *J*=8.1, *J*=7.0, *J*=1.0 Hz), 7.50 (d, 1H, *J*=8.9 Hz); ^{13}C-NMR (DMSO-d_6), δ: 169.90, 159.91, 139.90, 136.15, 130.60 (q, *J*=33.2 Hz), 129.42, 127.50, 126.08, 124.55, 123.13, 123.01 (q, *J*=273.4 Hz), 122.89, 121.30 (m), 118.16, 117.23 (m), 107.40; HR-MS: $[M-H]^+$ calculated 398.06102 m/z, found 398.06256 m/z.

N-(4-fluoro-2-(trifluoromethyl)phenyl)-1-hydroxynaphthalene-2-carboxamide (**7g**). Yield 24%; Mp. 135-137°C; HPLC purity 98.29%; IR (cm^{-1}): 3310, 1624, 1596, 1531, 1494, 1462, 1427, 1403, 1316, 1275, 1241, 1193, 1154, 1117, 1087, 1047, 1033, 1022, 963, 943, 911, 876, 830, 791, 779, 763, 744, 708, 664; ^{1}H-NMR (DMSO-d_6), δ: 13.80 (s, 1H), 10.64 (s, 1H), 8.30 (d, *J* = 8.3 Hz, 1H), 8.05 (d, *J* = 8.8 Hz, 1H), 7.92 (d, *J* = 8.3 Hz, 1H), 7.78 (dd, *J* = 8.8, 2.5 Hz, 1H), 7.65-7.76 (m, 3H), 7.57-7.62 (m, 1H), 7.49 (d, *J* = 8.6 Hz, 1H); ^{13}C-NMR (DMSO-d_6), δ: 170.69, 160.46 (d, *J* = 247.0 Hz), 159.78, 136.10, 133.84 (d, *J* = 9.1 Hz), 130.99 (dq, *J* = 3.6, 1.8 Hz), 129.25, 128.42 (qd, *J* = 30.9, 8.2 Hz), 127.25, 126.04, 124.59, 123.08, 122.95, 122.60 (qd, *J* = 274.3, 2.7 Hz), 120.34 (d, *J* = 21.8 Hz), 118.22, 114.23 (dq, *J* = 26.0, 5.5 Hz), 107.01; HR-MS: for $C_{18}H_{11}F_4NO_2$ $[M+H]^+$ calculated 350.079868 *m/z* found 350.08005 *m/z.*

N-[4-chloro-2-(trifluoromethyl)phenyl]-1-hydroxynaphthalene-2-carboxamide (**7h**). Yield 21%; Mp. 157-160°C; HPLC purity 100%; IR (cm^{-1}): 3480, 3371, 1620, 1613, 1591, 1520, 1498, 1464, 1412, 1392, 1306, 1288, 1236, 1210, 1165, 1147, 1118, 1090, 1053, 1033, 1022, 962, 942, 889, 837, 820, 791, 761, 721, 695, 658; ^{1}H-NMR (DMSO-d_6), δ: 13.69 (s, 1H), 10.72 (s, 1H), 8.31 (d, *J* = 8.3 Hz, 1H), 8.04 (d, *J* = 8.8 Hz, 1H), 7.87-7.95 (m, 3H), 7.75 (d, *J* = 8.3 Hz, 1H), 7.69 (t, *J* = 7.5 Hz, 1H), 7.57-7.62 (m, 1H), 7.49 (d, *J* = 8.8 Hz, 1H); ^{13}C-NMR (DMSO-d_6), δ: 170.32, 159.56, 136.12, 133.76 (q, *J* = 1.8 Hz), 133.34, 132.98, 132.28, 129.26, 127.93 (q, *J* = 30.9 Hz), 127.57, 126.67 (q, *J* = 5.5 Hz), 126.06, 124.60, 123.10, 123.07, 122.64 (q, *J* = 274.3 Hz), 118.32, 107.25; HR-MS: for $C_{18}H_{11}ClF_3NO_2$ $[M+H]^+$ calculated 366.050317 *m/z* found 366.05084 *m/z.*

N-[4-bromo-2-(trifluoromethyl)phenyl]-1-hydroxynaphthalene-2-carboxamide (**7i**). Yield 39%; Mp. 154-156°C; HPLC purity 100%; IR (cm^{-1}): 3476, 1641, 1631, 1606, 1572, 1523, 1504, 1484, 1461, 1410, 1392, 1323, 1311, 1288, 1268, 1238, 1166, 1151, 1131, 1104, 1085, 1052, 1022,

965, 890, 829, 812, 786, 763, 718, 680, 657; ^{1}H-NMR (DMSO-d_6), δ: 13.67 (s, 1H), 10.71 (s, 1H), 8.31 (d, *J* = 8.1 Hz, 1H), 7.99-8.06 (m, 3H), 7.93 (d, *J* = 8.1 Hz, 1H), 7.66-7.71 (m, 2H), 7.57-7.62 (m, 1H), 7.49 (d, *J* = 8.8 Hz, 1H); ^{13}C-NMR (DMSO-d_6), δ: 170.23, 159.52, 136.35, 136.12, 134.2 (q, *J* = 1.8 Hz), 133.10, 129.42 (q, *J* = 5.2 Hz), 129.26, 128.08 (q, *J* = 30.0 Hz), 127.58, 126.06, 124.60, 123.10, 123.08, 122.81 (q, *J* = 274.3 Hz), 120.32, 118.35, 107.29; HR-MS: for $C_{18}H_{11}BrF_3NO_2$ $[M-H]^+$ calculated 407.984145 *m/z* found 407.98526 *m/z.*

1-hydroxy-*N*-[4-nitro-2-(trifluoromethyl)phenyl]naphthalene-2-carboxamide (**7j**). Yield 36%; Mp. 193-197°C; HPLC purity 98.54%; IR (cm^{-1}): 3469, 1644, 1629, 1591, 1558, 1515, 1504, 1486, 1464, 1427, 1418, 1386, 1326, 1278, 1242, 1210, 1161, 1115, 1089, 1051, 941, 917, 869, 827, 800, 791, 767, 745, 721, 686; ^{1}H-NMR (DMSO-d_6), δ: 13.15 (br. s, 1H), 11.23 (s, 1H), 8.61 (dd, *J* = 8.8, 1.8 Hz, 1H), 8.55 (d, *J* = 1.8 Hz, 1H), 8.36 (d, *J* = 8.1 Hz, 1H), 8.31 (d, *J* = 9.1 Hz, 1H), 8.06 (d, *J* = 8.8 Hz, 1H), 7.94 (d, *J* = 8.1 Hz, 1H), 7.70 (t, *J* = 7.2 Hz, 1H), 7.59-7.64 (m, 1H), 7.54 (d, *J* = 8.8 Hz, 1H); ^{13}C-NMR (DMSO-d_6), δ: 168.49, 157.97, 144.69, 141.04 (q, *J* = 1.8 Hz), 136.30, 129.62, 129.24, 128.33, 127.68, 126.10, 124.75, 124.13 (q, *J* = 30.7 Hz), 123.94, 123.24, 122.53 (q, *J* = 273.7 Hz), 122.3 (q, *J* = 5.1 Hz), 119.09, 109.13; HR-MS: for $C_{18}H_{11}F_3N_2O_4$ $[M-H]^+$ calculated 375.058718 *m/z* found 375.05942 *m/z.*

1-hydroxy-*N*-[4-methoxy-3-(trifluoromethyl)phenyl]naphthalene-2-carboxamide (**7k**). Yield 74%; Mp. 183-186°C; HPLC purity 98.71%; IR (cm^{-1}): 3425, 1622, 1598, 1581, 1549, 1504, 1467, 1434, 1411, 1383, 1364, 1331, 1300, 1275, 1263, 1243, 1218, 1185, 1156, 1139, 1109, 1089, 1051, 1024, 966, 940, 921, 899, 874, 803, 789, 763, 717, 665; ^{1}H-NMR (DMSO-d_6), δ: 13.90 (s, 1H), 10.56 (s, 1H), 8.31 (d, *J* = 8.3 Hz, 1H), 8.09 (d, *J* = 8.8 Hz, 1H), 8.03 (s, 1H), 8.00 (d, *J* = 9.1 Hz, 1H), 7.92 (d, *J* = 8.1 Hz, 1H), 7.68 (t, *J* = 7.2 Hz, 1H), 7.56-7.61 (m, 1H), 7.48 (d, *J* = 8.8 Hz, 1H), 7.33 (d, *J* = 9.1 Hz, 1H), 3.91 (s, 3H); ^{13}C-NMR (DMSO-d_6), δ: 169.37, 159.94, 153.82 (q, *J* = 1.8 Hz), 135.99, 130.31, 129.16, 127.61, 127.48, 125.94, 124.64, 123.52 (q, *J* = 272.5 Hz), 123.08, 122.88, 120.59 (q, *J* = 5.5 Hz), 117.86, 116.59 (q, *J* = 30.9 Hz), 113.20, 107.30, 56.28; HR-MS: for $C_{19}H_{14}F_3NO_3$ $[M-H]^+$ calculated 360.084204 *m/z* found 360.08548 *m/z.*

1-hydroxy-*N*-[4-methyl-3-(trifluoromethyl)phenyl]naphthalene-2-carboxamide (**7l**). Yield 58%; Mp. 168-171°C; HPLC purity 98.80%; IR (cm^{-1}): 3429, 1629, 1594, 1575, 1537, 1504, 1469, 1422, 1410, 1388, 1358, 1326, 1286, 1267, 1246, 1225, 1207, 1162, 1146, 1138, 1106, 1086, 1054, 1033, 1023, 939, 924, 895, 873, 815, 803, 786, 759, 717, 668; ^{1}H-NMR (DMSO-d_6), δ: 13.79 (s, 1H), 10.62 (s, 1H), 8.32 (d, *J* = 8.4 Hz,

1H), 8.12 (s, 1H), 8.10 (d, *J* = 8.9 Hz, 1H), 7.96 (dd, *J* = 8.3, 2.0 Hz, 1H), 7.92 (d, *J* = 8.1 Hz, 1H), 7.68 (ddd, *J* = 8.2, 6.9, 1.2 Hz, 1H), 7.59 (ddd, *J* = 8.1, 6.9, 1.1 Hz, 1H), 7.46-7.50 (m, 2H), 2.44 (s, 3H); ^{13}C-NMR (DMSO-*d*$_6$), δ: 169.57, 159.96, 136.02, 135.97, 132.55, 131.76 (q, *J* = 1.8 Hz), 129.19, 127.46, 127.42 (q, *J* = 29.3 Hz), 125.93, 125.16, 124.61, 124.35 (q, *J* = 273.7 Hz), 123.07, 122.93, 118.79 (q, *J* = 6.2 Hz), 117.88, 107.37, 18.21; HR-MS: for $C_{19}H_{14}F_3NO_2$ $[M-H]^+$ calculated 344.08929 *m/z* found 344.09082 *m/z*.

N-[4-fluoro-3-(trifluoromethyl)phenyl]-1-hydroxynaphthalene-2-carboxamide (**7m**). Yield 53%; Mp. 164-166°C; HPLC purity 98.81%; IR (cm^{-1}): 3415, 1625, 1589, 1552, 1503, 1468, 1429, 1409, 1386, 1365, 1333, 1365, 1333, 1295, 1266, 1237, 1205, 1160, 1148, 1123, 1085, 1049, 1033, 939, 922, 893, 868, 852, 828, 803, 786, 757, 716, 664; ^{1}H-NMR (DMSO-*d*$_6$), δ: 13.65 (s, 1H), 10.71 (s, 1H), 8.31 (d, *J* = 8.3 Hz, 1H), 8.20 (dd, *J* = 6.3, 2.3 Hz, 1H), 8.06-8.13 (m, 2H), 7.92 (d, *J* = 8.1 Hz, 1H), 7.68 (t, *J* = 7.5 Hz, 1H), 7.55-7.61 (m, 2H), 7.49 (d, *J* = 9.1 Hz, 1H); ^{13}C-NMR (DMSO-*d*$_6$), δ: 169.60, 159.95, 155.26 (dq, *J* = 251.6, 2.7 Hz), 136.08, 134.53 (d, *J* = 2.7 Hz), 129.29, 128.01 (d, *J* = 9.1 Hz), 127.50, 126.02, 124.60, 123.10, 122.90, 122.48 (qd, *J* = 272.5, 1.8 Hz), 120.06 (q, *J* = 4.5 Hz), 118.01, 117.58 (d, *J* = 21.8 Hz), 116.42 (qd, *J* = 32.7, 12.7 Hz), 107.30; HR-MS: for $C_{18}H_{11}F_4NO_2$ $[M+H]^+$ calculated 350.079868 *m/z* found 350.08029 *m/z*.

N-[4-chloro-3-(trifluoromethyl)phenyl]-1-hydroxynaphthalene-2-carboxamide (**7n**). Yield 59%; Mp. 173-175°C; HPLC purity 98.70%; IR (cm^{-1}): 3447, 1632, 1597, 1532, 1504, 1485, 1468, 1393, 1316, 1300, 1269, 1232, 1207, 1143, 1132, 1103, 1089, 1032, 959. 888, 873, 829, 801, 787, 763, 750, 718, 683, 667; ^{1}H-NMR (DMSO-*d*$_6$), δ: 13.56 (s, 1H), 10.76 (s, 1H), 8.30-8.33 (m, 2H), 8.12 (dd, *J* = 8.8, 1.8 Hz, 1H), 8.08 (d, *J* = 9.1 Hz, 1H), 7.92 (d, *J* = 8.1 Hz, 1H), 7.77 (d, *J* = 8.8 Hz, 1H), 7.69 (t, *J* = 7.5 Hz, 1H), 7.57-7.62 (m, 1H), 7.50 (d, *J* = 8.8 Hz, 1H); ^{13}C-NMR (DMSO-*d*$_6$), δ: 169.70, 159.96, 137.37, 136.10, 131.99, 129.34, 127.50, 126.65 (q, *J* = 30.9 Hz), 126.38, 126.04, 125.36 (q, *J* = 1.8 Hz), 124.57, 123.12, 122.92, 122.71 (q, *J* = 273.4 Hz), 120.4 (q, *J* = 5.4 Hz), 118.04, 107.39; HR-MS: for $C_{18}H_{11}ClF_3NO_2$ $[M+H]^+$ calculated 366.050317 *m/z* found 366.05109 *m/z*.

N-[4-bromo-3-(trifluoromethyl)phenyl]-1-hydroxynaphthalene-2-carboxamide (**7o**). Yield 33%; Mp. 172-173°C; HPLC purity 98.22%; IR (cm^{-1}): 3440, 1626, 1591, 1575, 1532, 1502, 1468, 1411, 1389, 1360, 1326, 1287, 1270, 1259, 1242, 1228, 1208, 1174, 1126, 1100, 1020, 939, 902, 872, 856, 821, 802, 786, 763, 754, 722; ^{1}H-NMR (DMSO-*d*$_6$), δ: 13.55 (s, 1H), 10.75 (s, 1H), 8.32 (d, *J* = 8.1 Hz, 1H), 8.31 (s, 1H), 8.08 (d,

J = 9.1 Hz, 1H), 8.04 (dd, J = 8.8, 2.0 Hz, 1H), 7.92 (d, J = 8.1 Hz, 2H), 7.69 (t, J = 7.6 Hz, 1H), 7.57-7.62 (m, 1H), 7.50 (d, J = 8.8 Hz, 1H); ^{13}C-NMR (DMSO-d_6), δ: 169.70, 159.95, 137.83, 136.11, 135.40, 129.36, 128.47 (q, J = 30.9 Hz), 127.51, 126.41, 126.05, 124.57, 123.12, 122.94, 122.81 (q, J = 273.4 Hz), 120.69 (q, J = 5.5 Hz), 118.05, 113.10 (q, J = 1.8 Hz), 107.43; HR-MS: for $C_{18}H_{11}BrF_3NO_2$ $[M+H]^+$ calculated 409.999795 *m/z* found 410.00095 *m/z*.

1-hydroxy-*N*-[4-nitro-3-(trifluoromethyl)phenyl]naphthalene-2-carboxamide (**7p**). Yield 35%; Mp. 193-196°C; HPLC purity 99.55%; IR (cm^{-1}): 3389, 1634, 1615, 1544, 1520, 1505, 1463, 1413, 1391, 1326, 1305, 1268, 1247, 1208, 1179, 1154, 1134, 1119, 1090, 1039, 966, 952, 901, 890, 848, 822, 805, 792, 768, 751, 723, 695, 671; ^{1}H-NMR (DMSO-d_6), δ: 13.24 (br. s, 1H), 11.03 (s, 1H), 8.46 (d, J = 1.4 Hz, 1H), 8.37 (dd, J = 9.1, 1.6 Hz, 1H), 8.33 (d, J = 8.3 Hz, 1H), 8.28 (d, J = 8.8 Hz, 1H), 8.08 (d, J = 8.8 Hz, 1H), 7.93 (d, J = 8.1 Hz, 1H), 7.70 (t, J = 7.5 Hz, 1H), 7.58-7.63 (m, 1H), 7.52 (d, J = 8.8 Hz, 1H); ^{13}C-NMR (DMSO-d_6), δ: 169.97, 160.01, 142.75, 142.08, 136.22, 129.50, 127.51, 127.32, 126.10, 124.51, 124.45, 123.15, 122.98, 122.58 (q, J = 33.1 Hz), 122.05 (q, J = 273.4 Hz), 119.55 (q, J = 5.8 Hz), 118.19, 107.52; HR-MS: for $C_{18}H_{11}F_3N_2O_4$ $[M-H]^+$ calculated 375.058718 *m/z* found 375.06009 *m/z*.

1-hydroxy-*N*-[2-nitro-4-(trifluoromethyl)phenyl]naphthalene-2-carboxamide (**8d**). Yield 53%; Mp. 222-224°C; HPLC purity 98.39%; IR (cm^{-1}): 3324, 1651, 1627, 1589, 1530, 1505, 1472, 1406, 1394, 1350, 1320, 1297, 1263, 1206, 1148, 1115, 1087, 1070, 1021, 944, 908, 893, 844, 802, 790, 759, 723, 695, 672; ^{1}H-NMR (DMSO-d_6), δ: 13.01 (br.s, 1H), 11.57 (s, 1H), 8.43 (s, 1H), 8.35 (d, J = 8.1 Hz, 1H), 8.18-8.25 (m, 2H), 8.02 (d, J = 8.8 Hz, 1H), 7.95 (d, J = 8.1 Hz, 1H), 7.71 (t, J = 7.5 Hz, 1H), 7.59-7.64 (m, 1H), 7.55 (d, J = 8.8 Hz, 1H); ^{13}C-NMR (DMSO-d_6), δ: 168.77, 159.12, 142.10, 136.37, 134.55, 130.8 (q, J = 3.9 Hz), 129.56, 127.70, 127.18, 126.26, 125.64 (q, J = 33.7 Hz), 124.61, 123.44, 123.27, 123.06 (q, J = 272.6 Hz), 122.75 (q, J = 3.9 Hz), 118.87, 108.21; HR-MS: for $C_{18}H_{11}F_3N_2O_4$ $[M-H]^+$ calculated 375.058718 *m/z* found 375.06033 *m/z*.

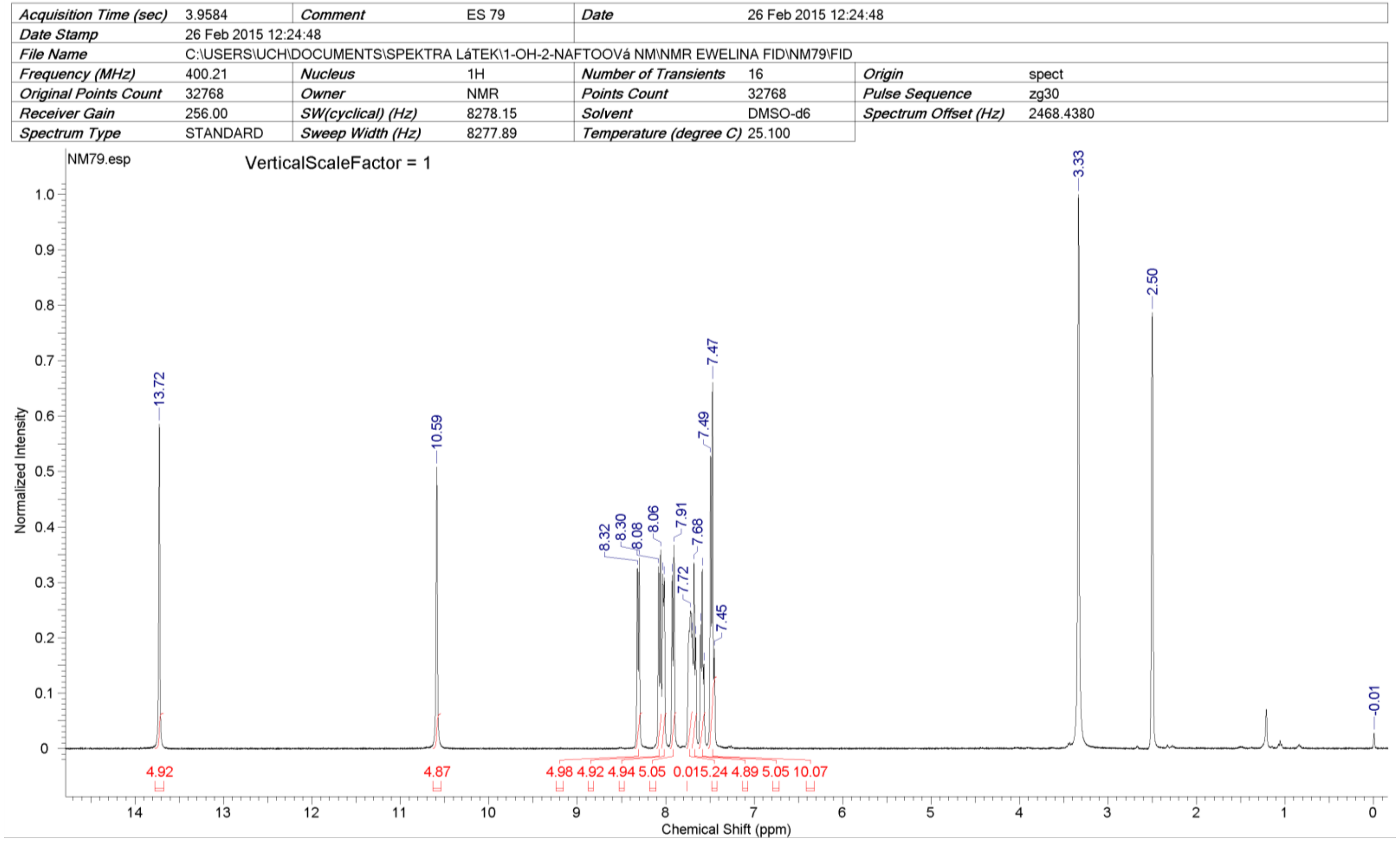

^{1}H NMR spectra of 5s

Acquisition Time (sec)	1.3664	*Comment*	ES 79 13C	*Date*	20 Jan 2016 10:27:28		
Date Stamp	20 Jan 2016 10:27:28						
File Name	C:\USERS\UCH\DOCUMENTS\SPEKTRA LáTEK\1-OH-2-NAFTOOVá NM\NMR EWELINA FID\ES79\FID					*Frequency (MHz)*	100.63
Nucleus	13C	*Number of Transients*	1024	*Origin*	spect	*Original Points Count*	32768
Owner	NMR	*Points Count*	32768	*Pulse Sequence*	zgpg30	*Receiver Gain*	20642.50
SW(cyclical) (Hz)	23980.81	*Solvent*	DMSO-d6	*Spectrum Offset (Hz)*	10010.8691	*Spectrum Type*	STANDARD
Sweep Width (Hz)	23980.08	*Temperature (degree C)*	25.000				

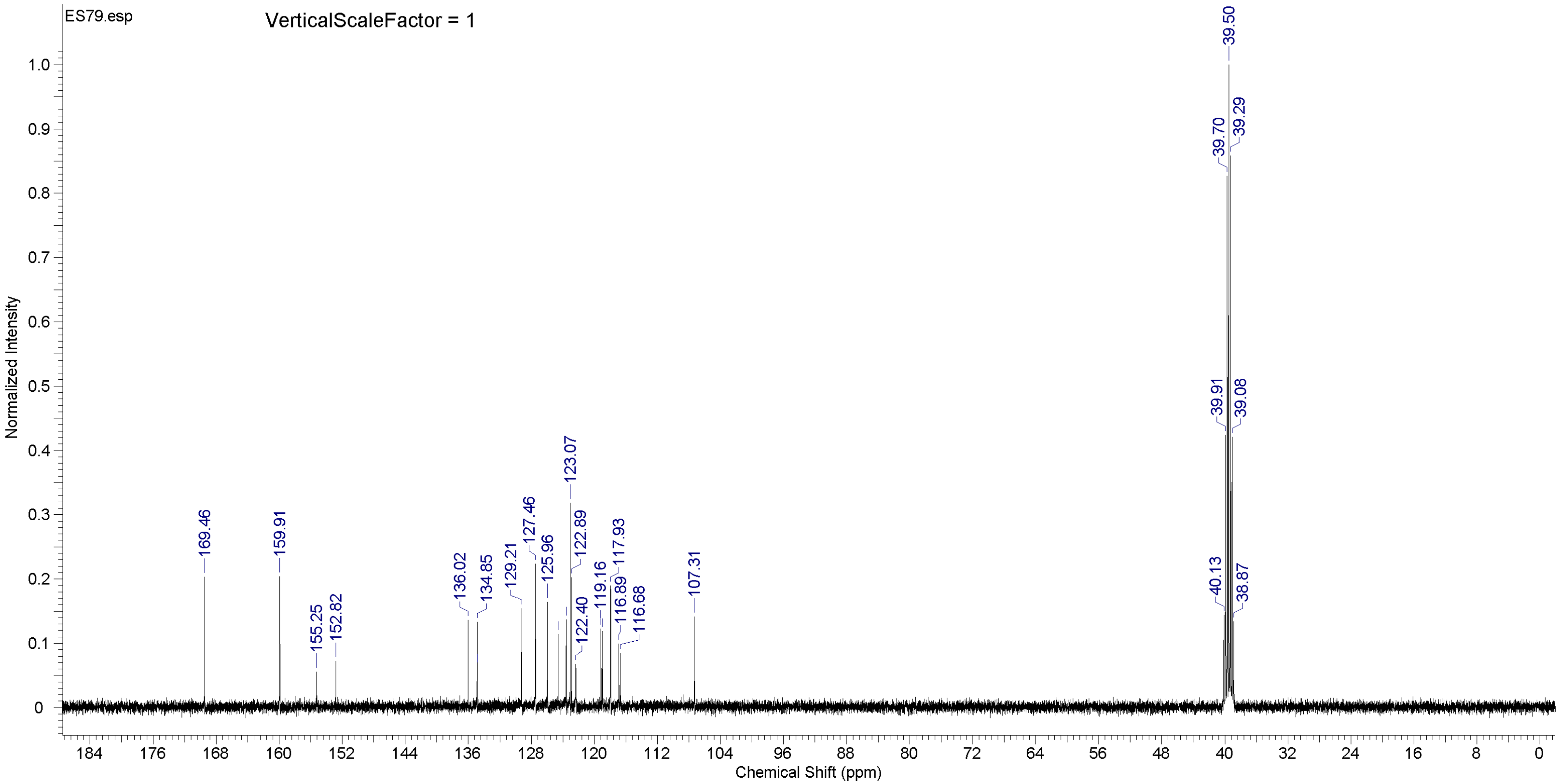

^{13}C NMR spectra of 5s

Acquisition Time (sec)	3.9584	*Comment*	ES 66	*Date*	19 Jan 2015 12:31:12		
Date Stamp	19 Jan 2015 12:31:12						
File Name	C:\USERS\UCH\DOCUMENTS\SPEKTRA LáTEK\1-OH-2-NAFTOOVá NM\NMR EWELINA FID\NM66\FID						
Frequency (MHz)	400.21	*Nucleus*	1H	*Number of Transients*	16	*Origin*	spect
Original Points Count	32768	*Owner*	NMR	*Points Count*	32768	*Pulse Sequence*	zg30
Receiver Gain	362.00	*SW(cyclical) (Hz)*	8278.15	*Solvent*	DMSO-d6	*Spectrum Offset (Hz)*	2468.6907
Spectrum Type	STANDARD	*Sweep Width (Hz)*	8277.89	*Temperature (degree C)*	25.100		

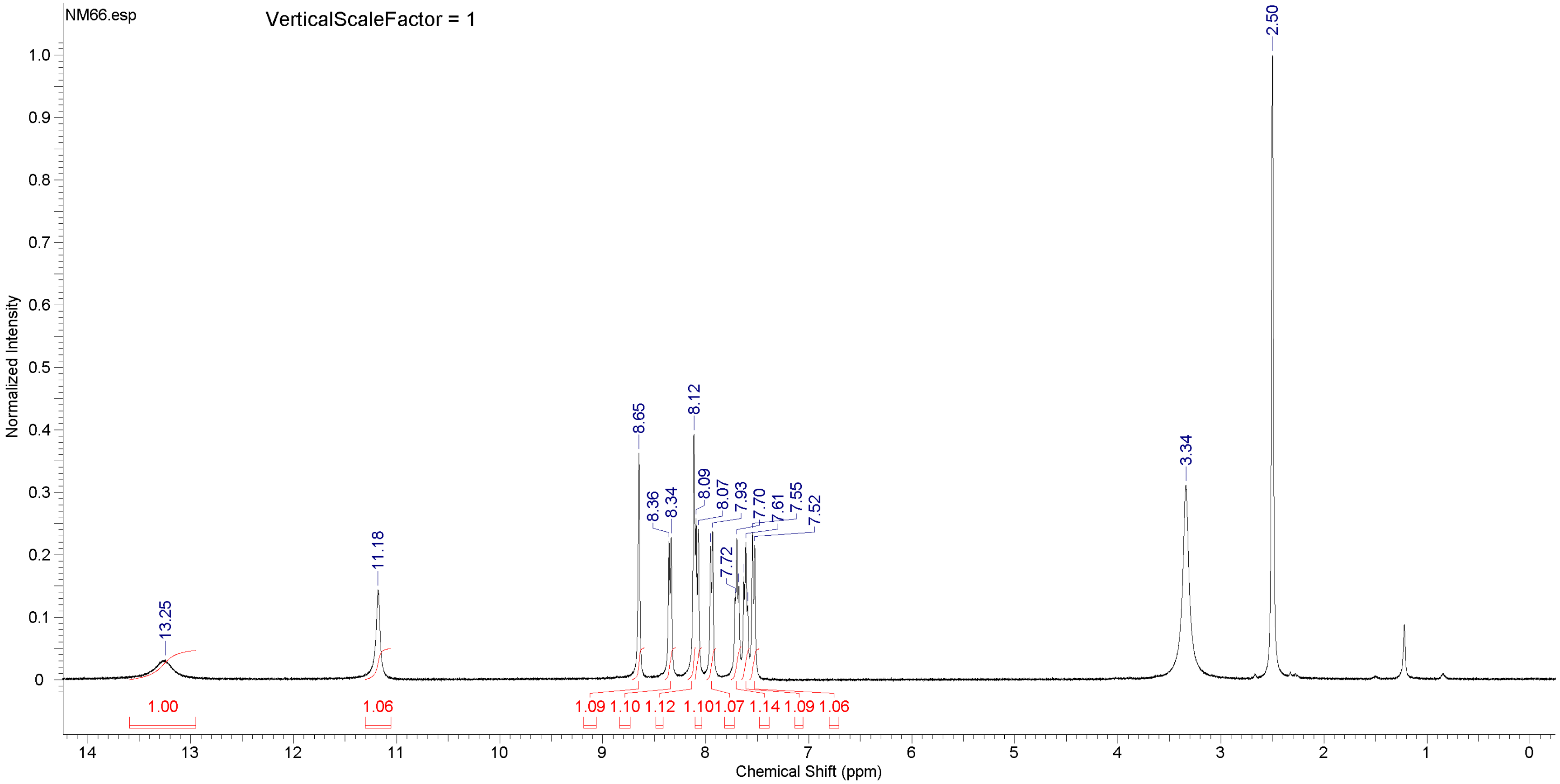

^{1}H NMR spectra of 5u

Acquisition Time (sec)	1.1010	*Comment*	ES 66 13C	*Date*	05 Apr 2016 13:37:36		
Date Stamp	05 Apr 2016 13:37:36						
File Name	C:\USERS\UCH\DOCUMENTS\SPEKTRA LáTEK\1-OH-2-NAFTOOVá NM\NMR EWELINA FID\ES66\FID					*Frequency (MHz)*	125.77
Nucleus	13C	*Number of Transients*	1024	*Origin*	spect	*Original Points Count*	32768
Owner	nmrsu	*Points Count*	32768	*Pulse Sequence*	zgpg30	*Receiver Gain*	1820.00
SW(cyclical) (Hz)	29761.90	*Solvent*	DMSO-d6	*Spectrum Offset (Hz)*	14403.4014	*Spectrum Type*	STANDARD
Sweep Width (Hz)	29761.00	*Temperature (degree C)*	24.998				

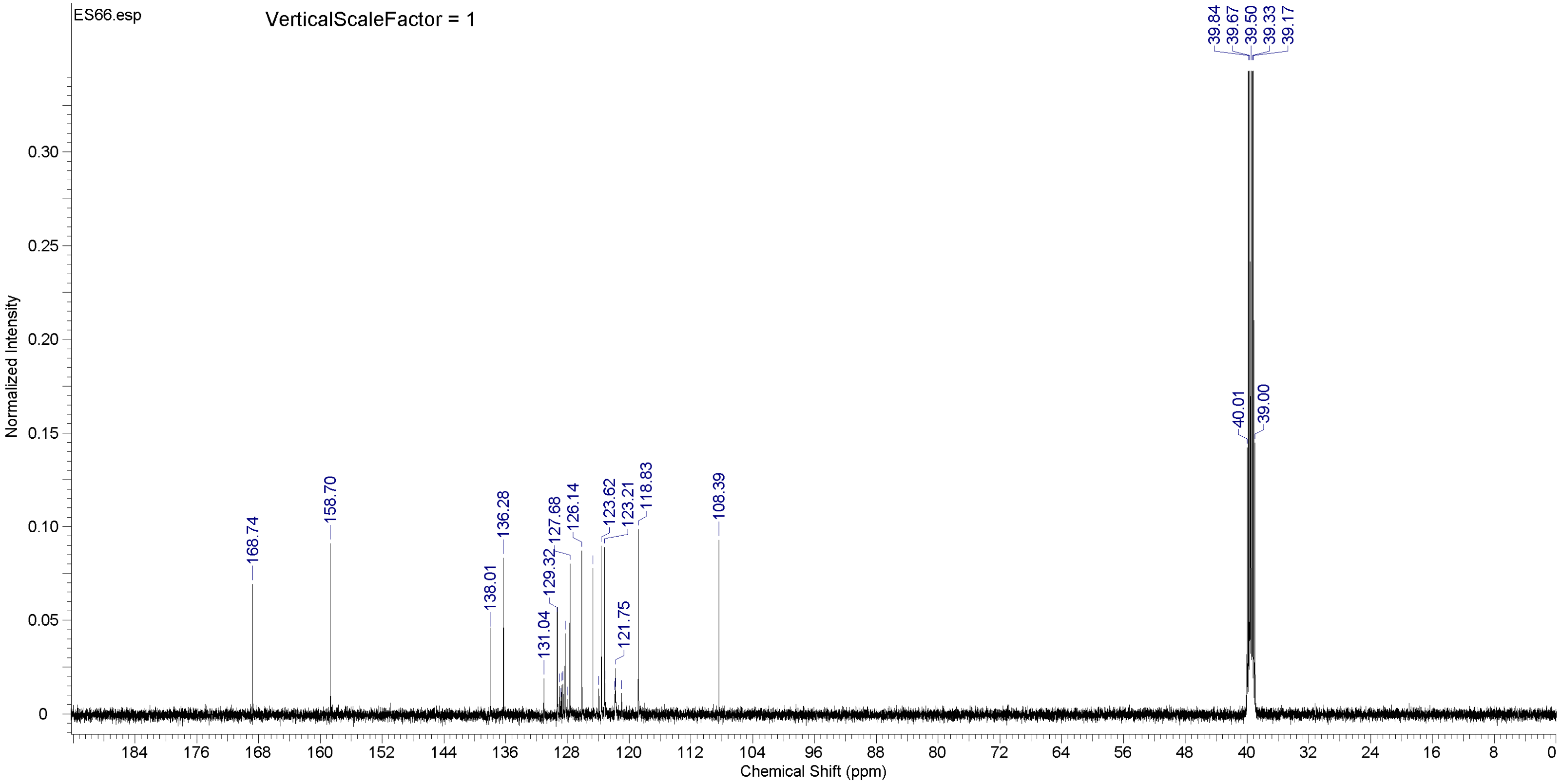

^{13}C NMR spectra of 5u

Table S1. Descriptors generated during the study

L.p.	Polar surface area	Polar volume	Surface area	Volume	Acceptor count Lipinski	Acceptor count Sybyl	Atom count	Bond count	clogP	Molecular weight	Hydrophobe count	Lipinski violations	Rotatable bond count
**1**	72.375	64.685	492.964	780.697	3	2	33	35	4.4462	263.2906	3	0	2
**2a**	75.247	83.936	537.719	852.648	4	3	37	39	4.8468	281.2811	3	0	3
**2b**	84.752	95.837	539.449	865.476	4	3	37	39	2.4880	376.2861	3	0	3
**2c**	84.759	112.486	539.430	884.195	4	3	37	39	5.2372	361.3146	3	0	3
**3a**	68.310	65.782	512.343	825.931	3	2	36	38	5.8272	361.3146	3	0	2
**3b**	72.383	65.252	524.826	835.400	3	2	36	38	4.5668	297.7357	3	0	2
**3c**	72.405	67.988	525.372	836.989	3	2	36	38	5.4168	297.7357	3	0	2
**4a**	71.408	65.633	498.585	795.973	3	2	33	35	5.4168	297.7357	3	0	2
**4b**	72.458	63.874	501.158	796.600	3	2	33	35	4.6868	342.1867	3	0	2
**4c**	72.397	62.590	502.668	795.190	3	2	33	35	5.5668	342.1867	3	0	2
**5a**	72.146	56.347	506.774	816.208	3	2	33	35	5.5668	342.1867	3	0	2
**5b**	72.427	67.848	515.053	819.984	3	2	33	35	4.3300	331.2886	3	1	2
**5c**	72.380	61.492	516.481	822.076	3	2	33	35	5.7800	331.2886	3	1	2
**6a**	72.531	58.315	508.989	827.084	3	2	33	35	5.7800	331.2886	3	0	2
**6b**	72.406	66.058	519.865	829.793	3	2	33	35	3.9316	293.3166	3	1	2
**6c**	72.419	60.605	520.270	827.912	3	2	33	35	1.1542	308.2882	3	1	2
**7a**	65.804	58.484	528.321	845.305	3	2	36	38	1.1542	308.2882	4	0	2
**7b**	72.386	63.925	548.085	875.698	3	2	36	38	1.1542	308.2882	4	1	2
**7c**	72.366	68.510	548.287	883.504	3	2	36	38	5.3700	332.1807	4	1	2
**8a**	136.587	124.365	515.897	823.297	6	4	35	37	4.5200	332.1807	3	0	3
**8b**	159.021	136.843	531.238	839.539	6	4	35	37	6.1000	332.1807	3	0	3
**8c**	158.425	135.937	529.774	847.723	6	4	35	37	6.2200	332.1807	3	0	3
**5f**	72.115	63.156	530.158	858.741	3	2	33	35	3.8800	299.2715	3	1	2
**5g**	69.360	71.846	523.897	859.127	3	2	33	35	5.0800	299.2715	3	0	2
**5h**	72.437	58.225	536.154	850.208	3	2	33	35	4.7600	421.0827	3	1	2
**5i**	72.395	69.851	539.541	869.350	3	2	33	35	4.5216	293.3166	3	1	2
**4f**	68.400	72.062	502.308	818.582	3	2	33	35	6.8208	399.2866	3	0	2
**4g**	72.390	65.609	511.110	815.727	3	2	33	35	5.7332	365.7336	3	1	2
**6f**	70.143	75.450	531.146	879.736	3	2	33	35	4.7942	291.3438	3	0	2
**7f**	71.373	66.866	600.826	965.776	3	2	39	41	4.1442	291.3438	5	1	2
**5r**	70.965	67.244	560.312	922.864	3	2	36	38	5.4442	291.3438	4	1	2
**3d**	67.624	66.370	544.313	874.813	3	2	39	41	3.9563	323.3426	3	0	2

**3e**	56.453	80.435	532.986	885.349	3	2	39	41	4.5463	323.3426	3	0	2
**3f**	71.644	73.288	556.720	890.388	3	2	39	41	4.4800	299.2715	3	1	2
**2d**	87.516	98.267	585.511	937.054	5	4	41	43	5.0100	299.2715	3	0	4
**2e**	96.300	121.749	588.299	949.143	5	4	41	43	4.4800	299.2715	3	0	4
**4d**	71.380	60.023	510.028	811.609	3	2	33	35	4.5216	293.3166	3	0	2
**4e**	72.438	62.296	510.828	809.024	3	2	33	35	4.0545	317.2620	3	1	2
**4h**	71.346	68.907	508.758	815.434	3	2	33	35	4.5145	317.2620	3	0	2
**4i**	68.485	66.932	512.010	828.906	3	2	33	35	4.5845	317.2620	3	0	2
**4j**	71.410	63.831	516.278	823.748	3	2	33	35	5.1145	317.2620	3	0	2
**4k**	71.330	64.739	519.209	830.024	3	2	33	35	4.1386	335.2525	3	0	2
**4i**	72.381	60.378	519.965	827.196	3	2	33	35	4.2854	353.2429	3	1	2
**4m**	68.536	72.601	520.886	844.499	3	2	33	35	5.2500	332.1807	3	0	2
**4n**	68.471	70.826	529.446	855.819	3	2	33	35	5.3700	332.1807	3	0	2
**5d**	72.192	64.390	528.042	853.638	3	2	33	35	5.9945	366.6258	3	1	2
**5e**	72.135	57.799	530.176	851.217	3	2	33	35	5.2645	366.6258	3	1	2
**5j**	72.086	61.073	551.356	896.195	3	2	33	35	4.2952	277.3172	3	1	2
**5k**	69.333	74.297	547.246	908.670	3	2	33	35	6.7245	366.6258	3	1	2
**5l**	72.380	60.940	558.017	891.694	3	2	33	35	5.6400	421.0827	3	1	2
**6d**	72.503	52.103	537.766	869.383	3	2	33	35	5.6400	421.0827	3	1	2
**6e**	72.490	64.099	537.671	875.949	3	2	33	35	5.6545	499.9788	3	1	2
**6g**	70.164	74.358	559.451	936.748	3	2	33	35	5.3708	399.2866	3	1	2
**7d**	65.797	52.958	582.930	939.251	3	2	39	41	5.3708	399.2866	5	1	2
**7e**	64.649	60.284	581.130	955.277	3	2	39	41	6.5153	433.7316	5	1	2
**5u**	71.673	78.278	606.476	1008.981	3	2	39	41	3.8064	353.3686	5	1	2
**2f**	96.229	161.602	626.310	1025.587	6	5	45	47	4.6432	305.3704	3	0	5
**3g**	56.449	90.329	565.340	944.437	3	2	42	44	4.3706	307.3432	3	0	2
**3h**	80.648	83.457	560.362	911.990	4	3	40	42	4.9452	277.3172	3	0	3
**2j**	74.417	83.358	568.853	899.040	4	3	40	42	4.4306	307.3432	3	0	3
**2k**	67.627	96.380	554.806	910.089	4	3	40	42	3.7806	307.3432	3	0	3
**2g**	161.715	153.492	575.430	913.246	7	5	39	41	0.6396	338.3142	3	0	4
**2h**	162.082	166.913	574.883	923.262	7	5	39	41	0.6396	338.3142	3	0	4
**5p**	84.041	80.258	554.004	902.299	4	3	37	39	4.5825	327.7617	3	0	3
**5m**	72.104	58.648	516.484	829.740	3	2	33	35	4.8000	315.7261	3	0	2
**4o**	71.419	65.252	520.942	831.559	3	2	33	35	5.0500	315.7261	3	1	2
**5n**	72.405	63.502	523.949	830.097	3	2	33	35	5.6500	315.7261	3	1	2
**4q**	71.391	58.144	523.958	837.964	3	2	33	35	5.0500	315.7261	3	1	2
**4s**	71.362	69.179	522.692	836.978	3	2	33	35	5.0500	315.7261	3	1	2
**6j**	72.450	61.747	518.905	842.116	3	2	33	35	4.9452	277.3172	3	0	2
**4r**	71.403	63.237	527.003	841.091	3	2	33	35	4.9200	360.1771	3	1	2

**4v**	72.448	58.180	528.772	838.049	3	2	33	35	5.2000	360.1771	3	1	2
**4t**	71.387	68.097	527.597	845.605	3	2	33	35	5.8000	360.1771	3	1	2
**5t**	72.412	53.472	540.649	855.979	3	2	33	35	5.2000	360.1771	3	1	2
**6h**	72.575	56.224	533.556	860.696	3	2	33	35	6.2300	376.6317	3	1	2
**5s**	72.146	55.361	535.203	861.259	3	2	33	35	5.4900	376.6317	3	1	2
**5q**	72.120	61.188	535.040	865.053	3	2	33	35	5.5200	376.6317	3	1	2
**6m**	70.197	64.189	560.191	913.618	3	2	33	35	5.5200	376.6317	3	1	2
**4y**	68.479	70.655	546.488	891.225	3	2	33	35	5.5186	473.5183	3	1	2
**5o**	72.092	69.730	561.455	915.207	3	2	36	38	5.0054	414.1485	4	1	2
**7h**	65.792	57.704	551.907	887.337	3	2	36	38	4.2468	281.2811	4	1	2
**7n**	72.359	59.541	561.807	906.713	3	2	36	38	5.7332	365.7336	4	1	2
**5v**	69.341	87.003	579.449	958.230	3	2	36	38	5.1332	365.7336	4	1	2
**4w**	72.440	67.127	551.797	887.095	3	2	36	38	6.3832	365.7336	4	1	2
**4x**	71.257	71.452	555.007	899.932	3	2	36	38	5.6277	400.1787	4	1	2
**7g**	65.772	53.991	538.021	856.797	3	2	36	38	6.0132	349.2791	4	0	2
**7m**	72.396	65.329	552.393	890.084	3	2	36	38	6.0132	349.2791	4	1	2
**4p**	71.403	64.083	548.987	876.233	3	2	36	38	4.5632	349.2791	4	1	2
**4u**	70.158	65.664	550.883	899.249	3	2	36	38	6.0132	349.2791	4	1	2
**4z**	71.370	73.018	559.354	903.472	3	2	36	38	5.4132	349.2791	4	1	2
**7i**	65.795	60.447	556.724	895.881	3	2	36	38	5.4132	349.2791	4	1	2
**6k**	71.344	69.389	562.712	936.624	3	2	36	38	4.8468	281.2811	4	1	2
**6i**	72.492	66.914	564.352	922.786	3	2	36	38	5.5877	367.2695	4	1	2
**7o**	72.357	59.124	565.442	916.532	3	2	36	38	5.2832	410.1846	4	1	2
**6l**	70.177	89.580	586.765	974.676	3	2	36	38	5.8532	410.1846	4	1	2
**3i**	67.197	71.649	566.084	915.286	3	2	39	41	5.8532	410.1846	4	1	2
**7l**	72.359	61.097	567.543	915.328	3	2	39	41	6.7332	410.1846	4	1	2
**8d**	136.602	134.134	571.046	924.915	6	4	38	40	5.8677	489.0807	4	0	3
**7j**	151.829	133.775	565.199	911.063	6	4	38	40	5.6290	345.3152	4	0	3
**7p**	142.920	134.701	574.548	925.302	6	4	38	40	6.2790	345.3152	4	0	3
**2i**	74.053	80.037	590.548	943.031	4	3	40	42	2.4880	376.2861	4	1	3
**7k**	72.806	91.368	590.336	941.290	4	3	40	42	1.0380	376.2861	4	1	3

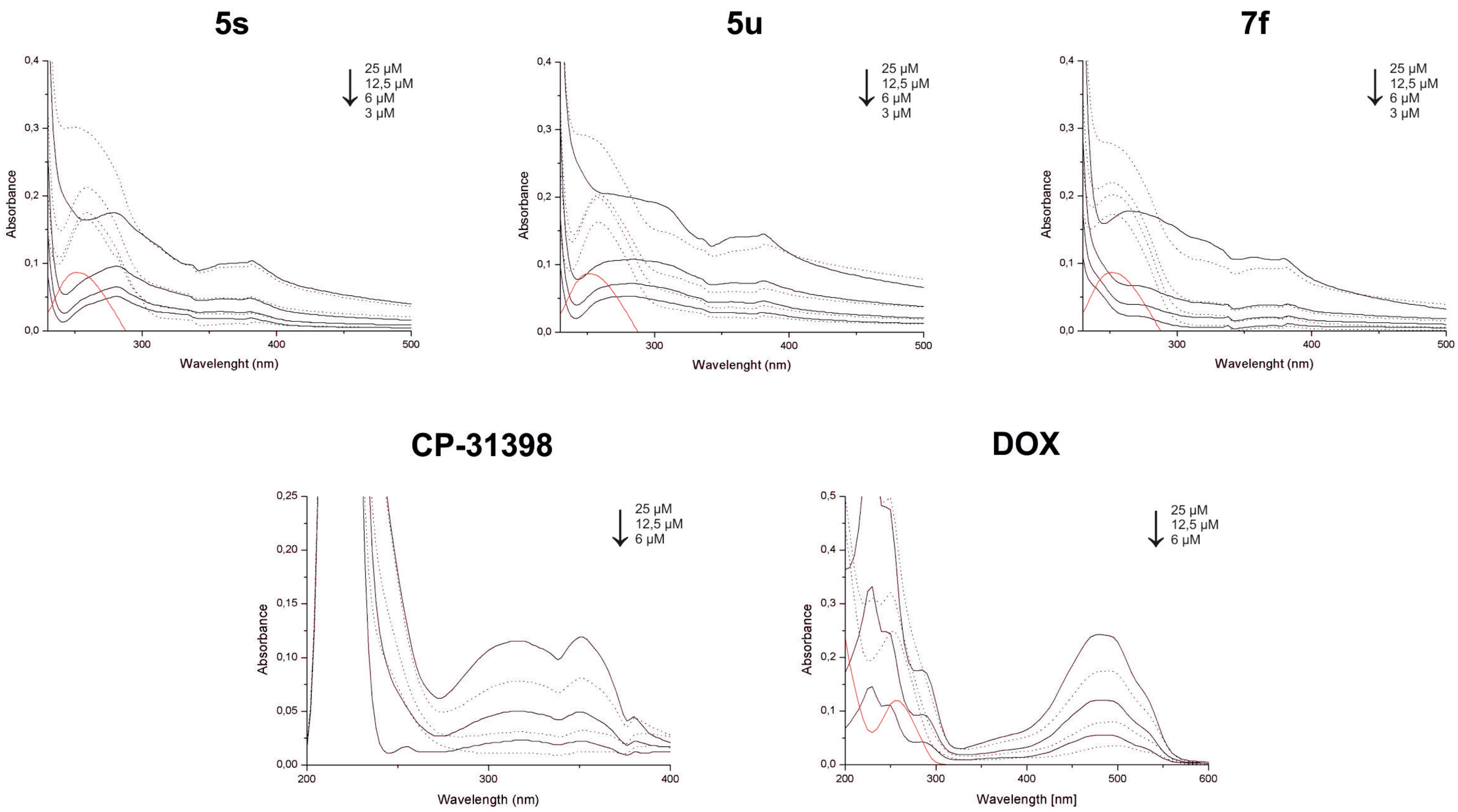

Figure S1. Spectroscopic analysis of the DNA interaction with the tested compounds. Spectra of the tested compounds without CT-DNA (solid line), with CT-DNA (dotted line) and CT-DNA (red line) in PBS.

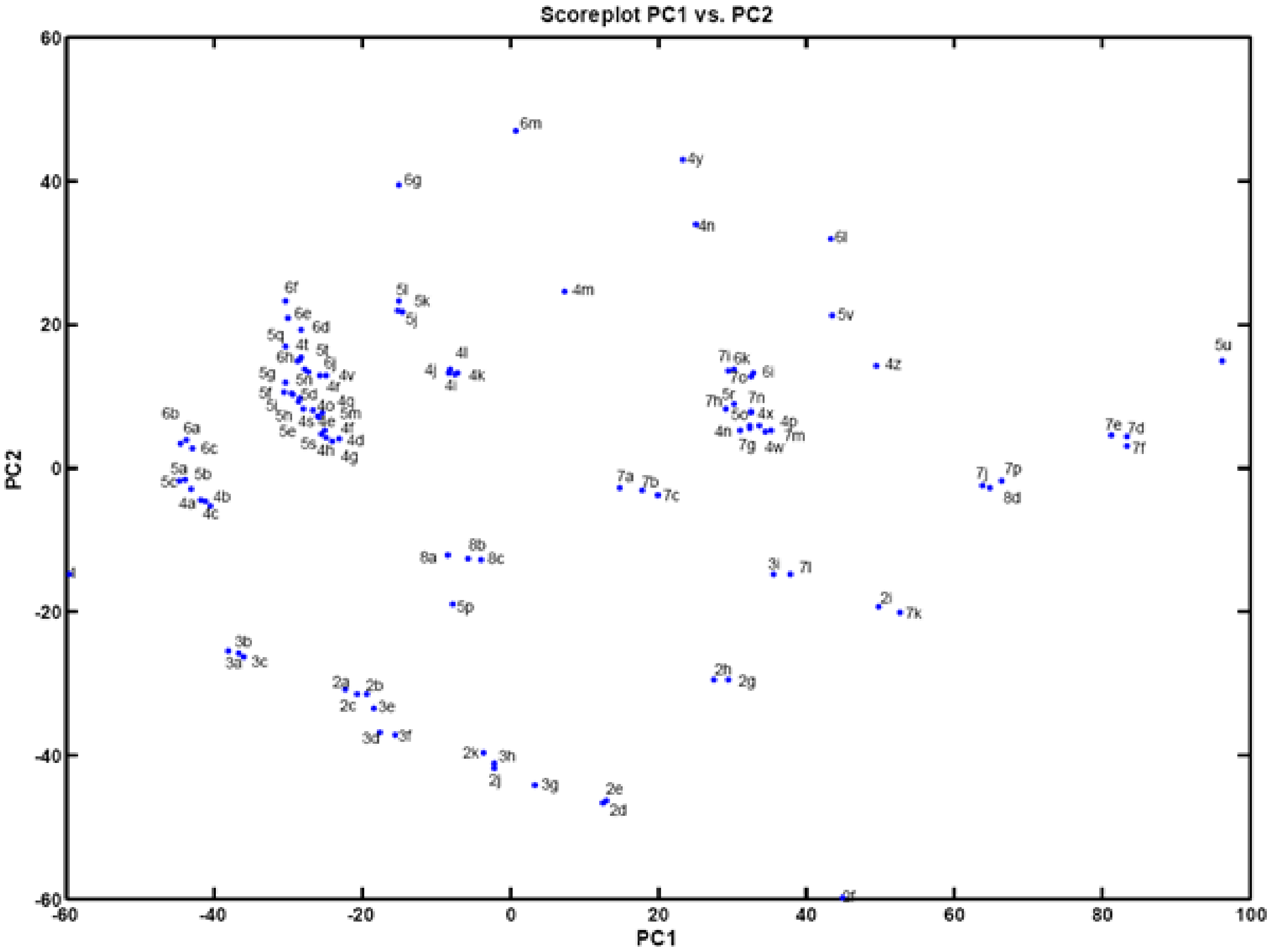

Figure S2. Projection of hydroxynaphthanilides **1-8d** on the plane that was defined by the first vs. second principal components for the Dragon descriptors.

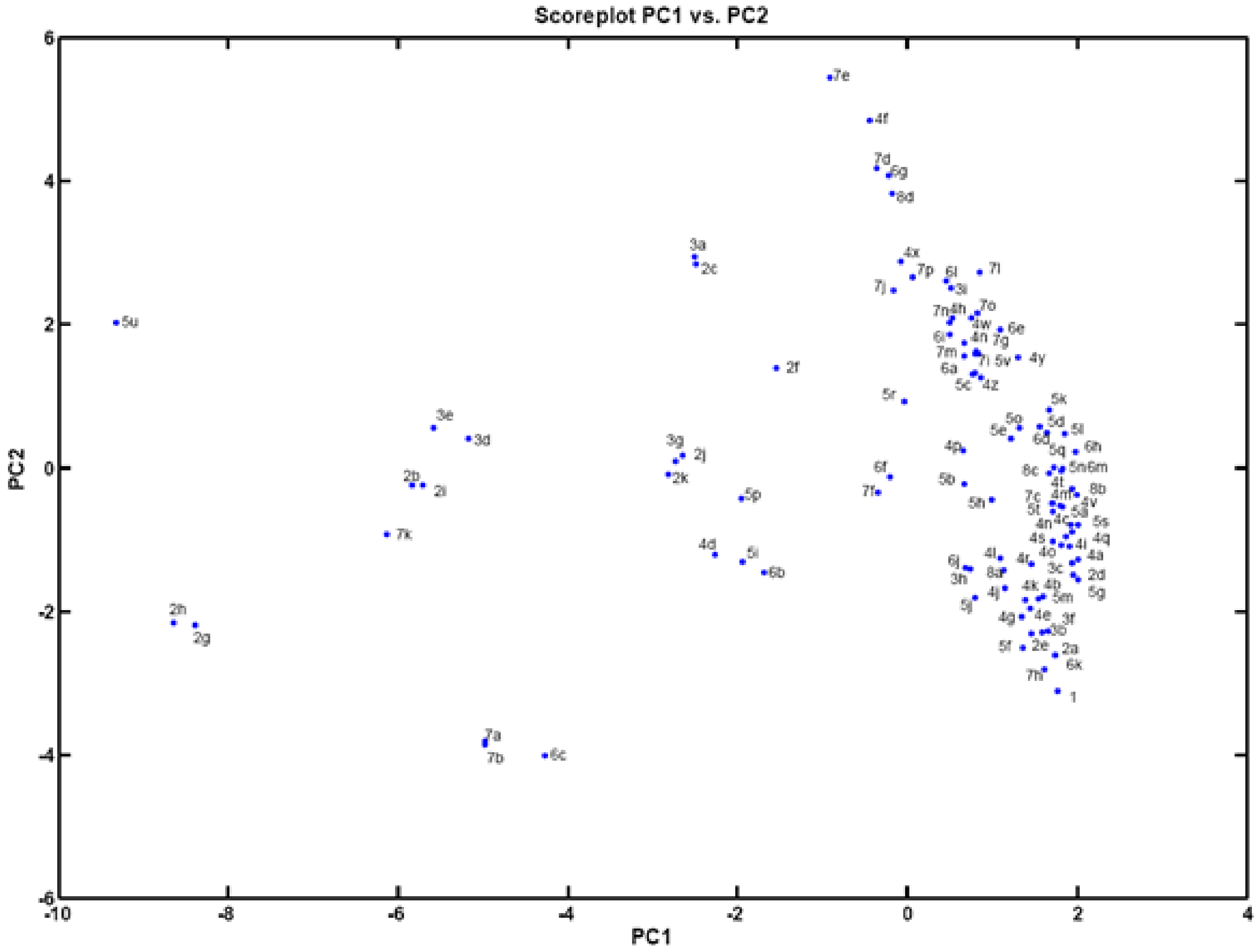

Figure S3. Projection of hydroxynaphthanilides **1-8d** on the plane that was defined by the first vs. second principal components for the Sybyl descriptors.

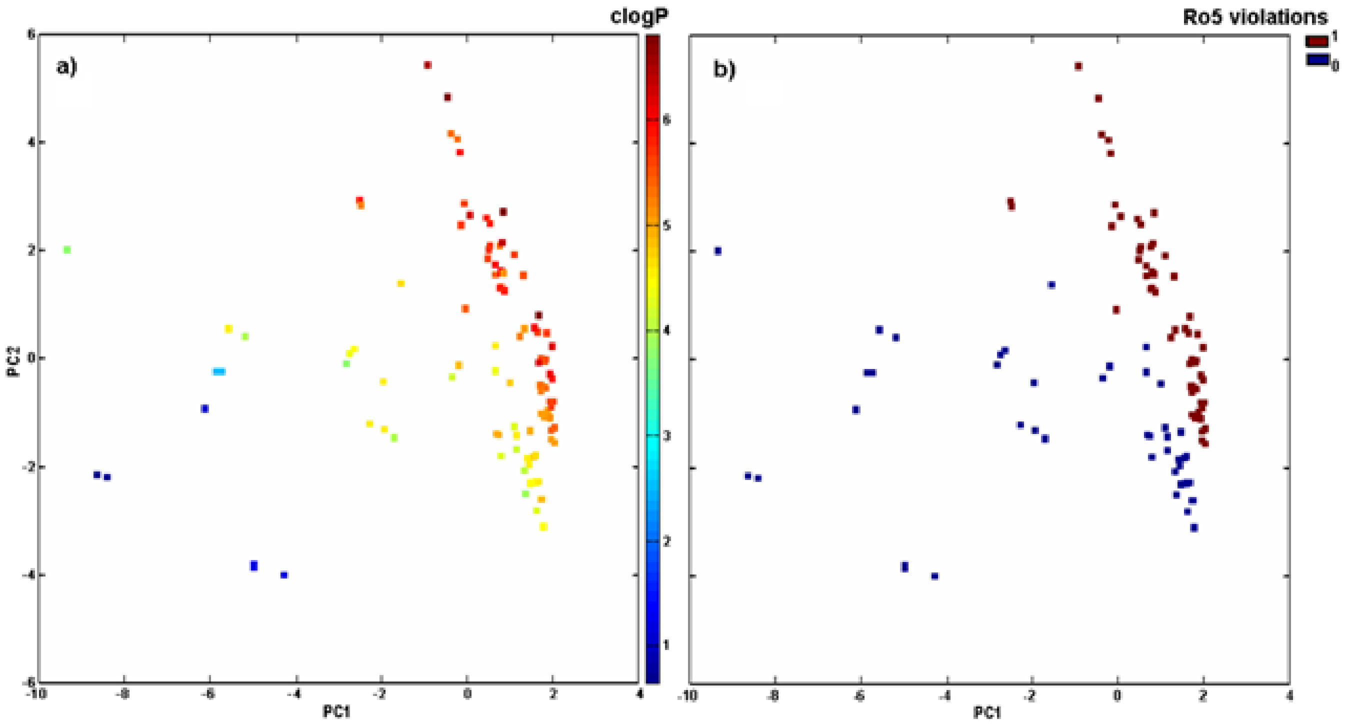

Figure S4. Projection of hydroxynaphthanilides **1-8d** on the plane that was defined by the first vs. second principal component with its molecule lipophilicity profile **(a)** and the number of Ro5 rule violations **(b)**. Color code the value of the lipophilicity that was calculated and the number of Ro5 violations.

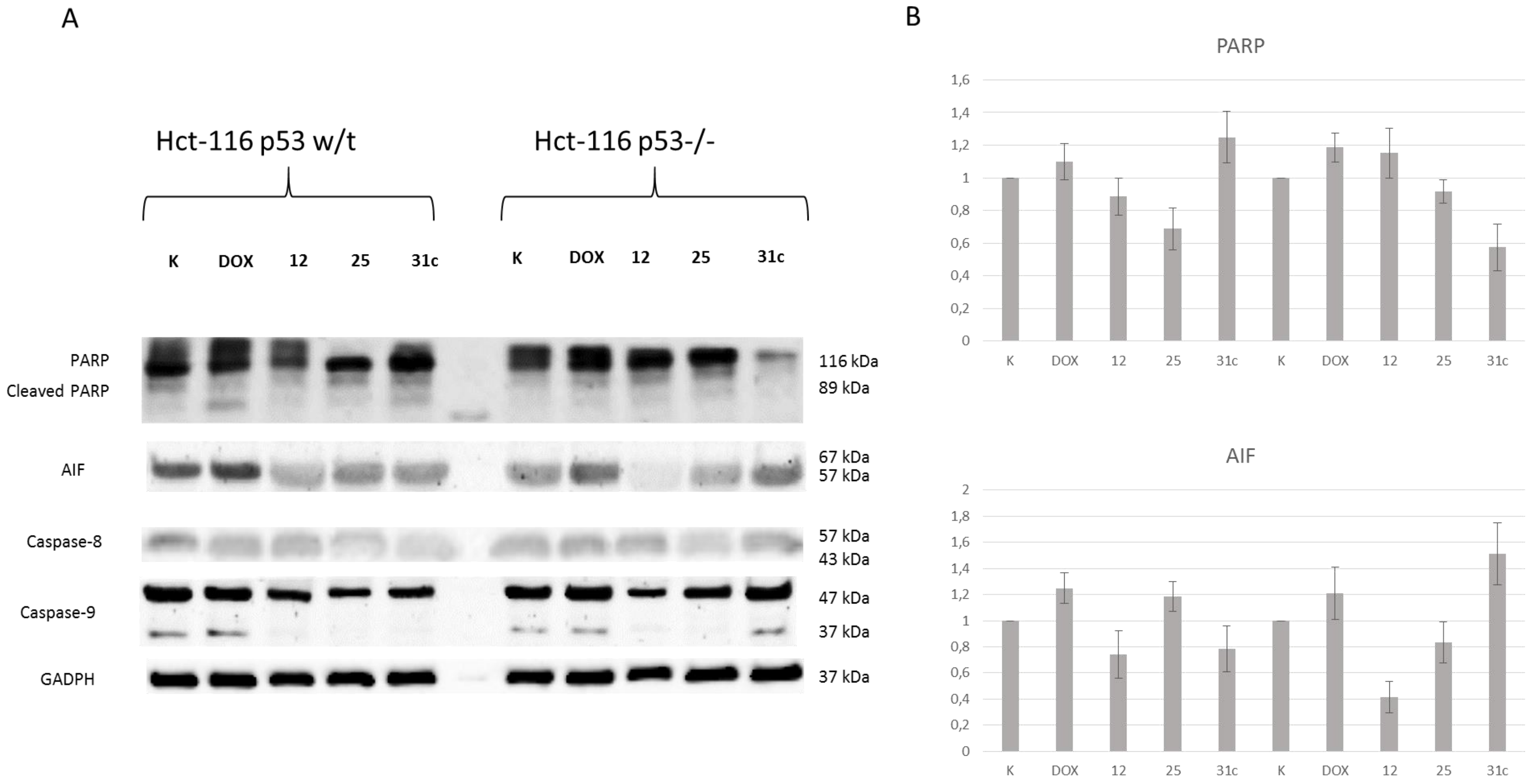

Figure S5. Effect of the tested anilides on PARP cleavage and the expression of the proteins that are involved in cell death. (**A**). A densitometric analysis of the expression of the PARP cleavage and AIF proteins normalized to GAPDH. The results are the mean ± SD of three independent experiments (**B**).).

Figure S6. Images of the gels prepared during this study. All gels uncropped and unmodified. Proteins relevant to this study are marked in boxes along with reference proteins. Colors used to distinguish appropriate protein pairs.

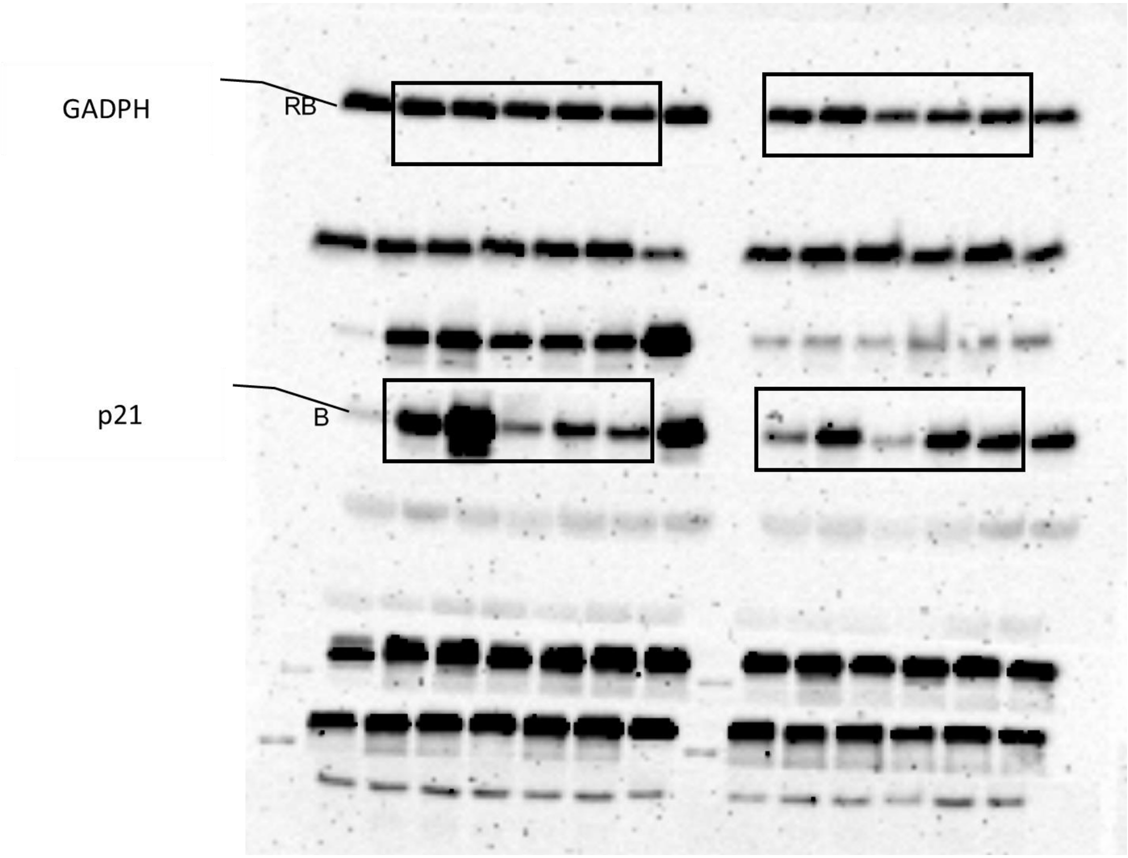

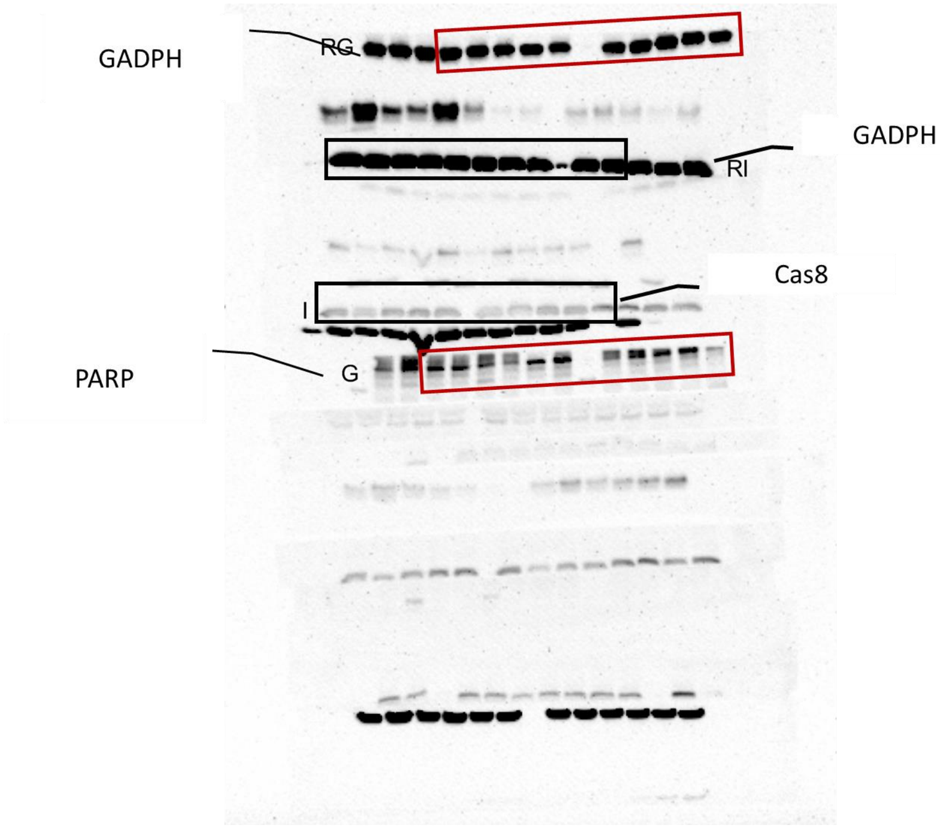
GADPH
RG
GADPH
RI
Cas8
I
PARP
G

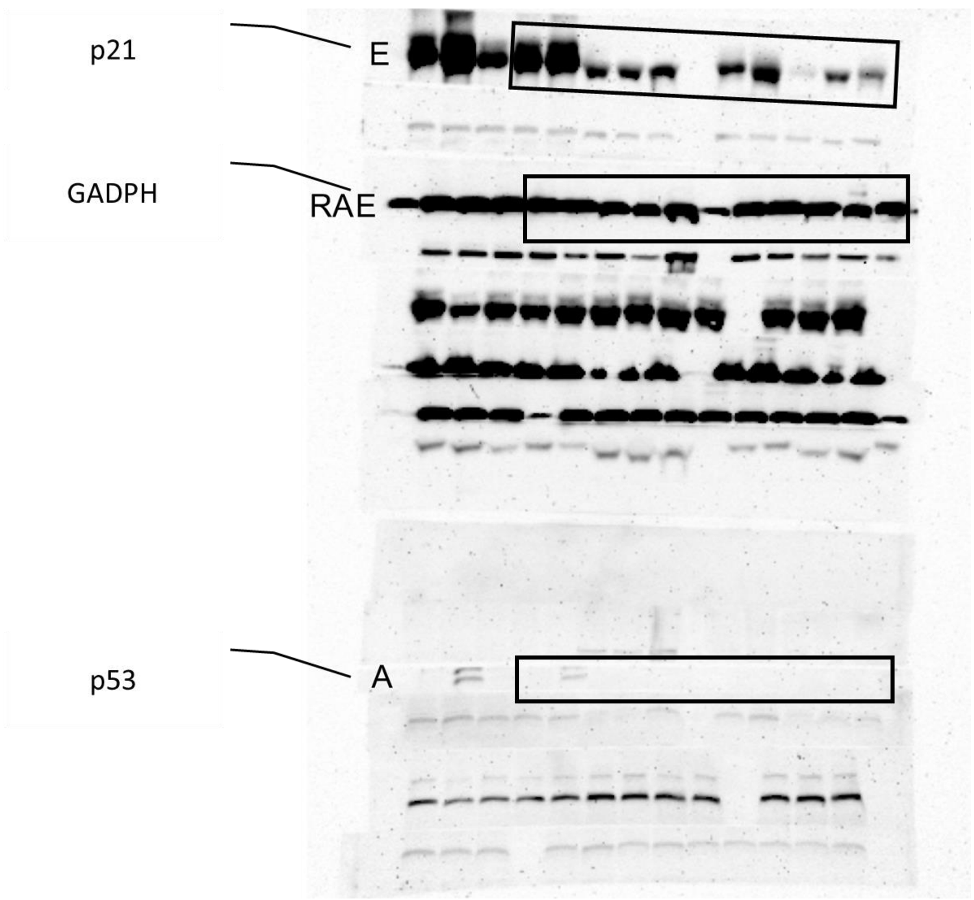
p21
E
GADPH
RAE
p53
A

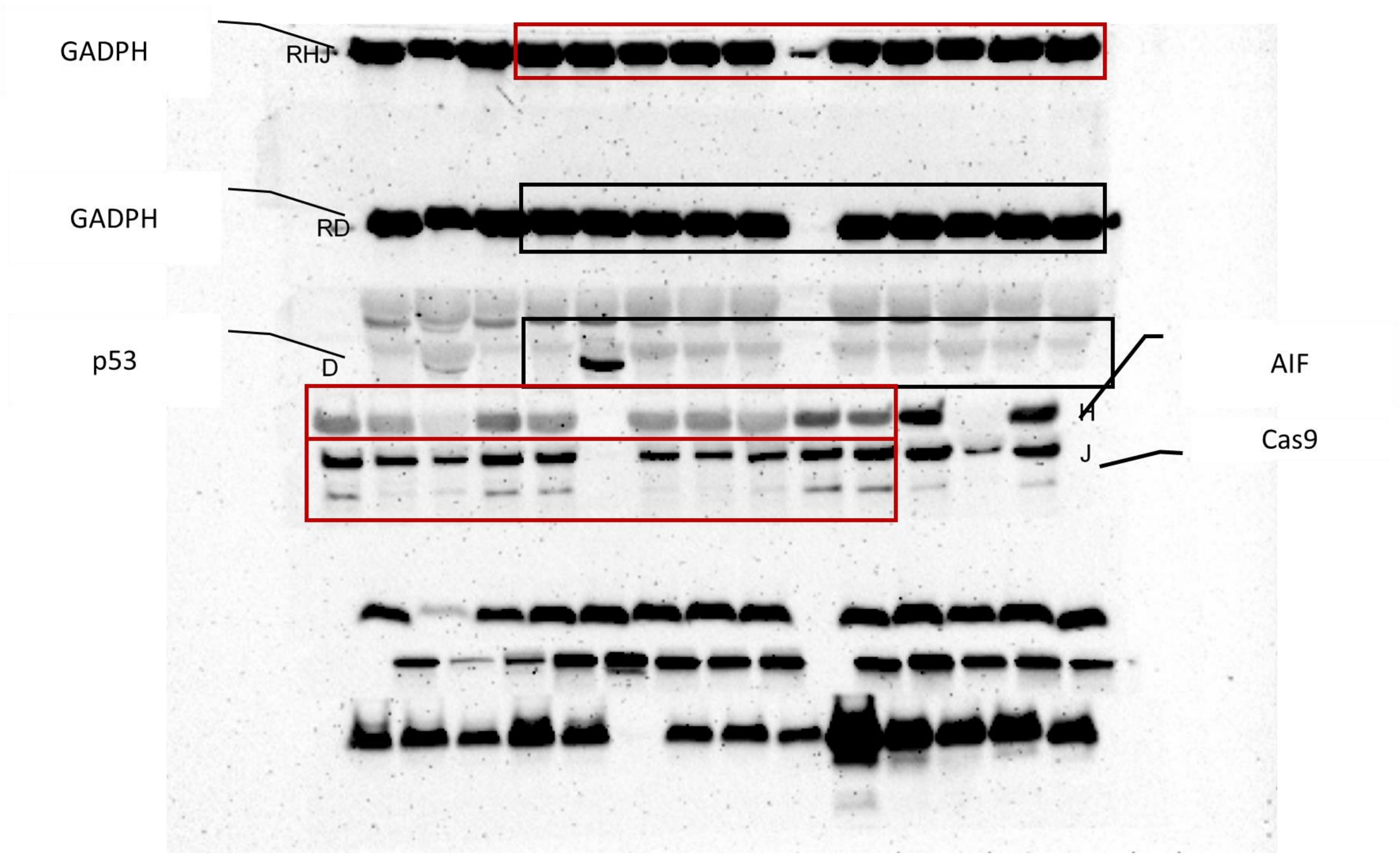
GADPH
RHJ
GADPH
RD
p53
D
AIF
H
Cas9
J
